# Identification of repurposing therapeutics toward SARS-CoV-2 main protease by virtual screening

**DOI:** 10.1371/journal.pone.0269563

**Published:** 2022-06-30

**Authors:** Kamonpan Sanachai, Tuanjai Somboon, Patcharin Wilasluck, Peerapon Deetanya, Peter Wolschann, Thierry Langer, Vannajan Sanghiran Lee, Kittikhun Wangkanont, Thanyada Rungrotmongkol, Supot Hannongbua

**Affiliations:** 1 Center of Excellence in Computational Chemistry (CECC), Department of Chemistry, Chulalongkorn University, Bangkok, Thailand; 2 Center of Excellence in Biocatalyst and Sustainable Biotechnology, Department of Biochemistry, Chulalongkorn University, Bangkok, Thailand; 3 Center of Excellence for Molecular Biology and Genomics of Shrimp, Department of Biochemistry, Chulalongkorn University, Bangkok, Thailand; 4 Molecular Crop Research Unit, Department of Biochemistry, Chulalongkorn University, Bangkok, Thailand; 5 Department of Pharmaceutical Chemistry, Faculty of Life Sciences, University of Vienna, Vienna, Austria; 6 Institute of Theoretical Chemistry, University of Vienna, Vienna, Austria; 7 Department of Chemistry, University of Malaya, Kuala Lumpur, Malaysia; 8 Program in Bioinformatics and Computational Biology, Graduate School, Chulalongkorn University, Bangkok, Thailand; Alagappa University, INDIA

## Abstract

SARS-CoV-2 causes the current global pandemic coronavirus disease 2019. Widely-available effective drugs could be a critical factor in halting the pandemic. The main protease (3CL^pro^) plays a vital role in viral replication; therefore, it is of great interest to find inhibitors for this enzyme. We applied the combination of virtual screening based on molecular docking derived from the crystal structure of the peptidomimetic inhibitors (N3, 13b, and 11a), and experimental verification revealed FDA-approved drugs that could inhibit the 3CL^pro^ of SARS-CoV-2. Three drugs were selected using the binding energy criteria and subsequently performed the 3CL^pro^ inhibition by enzyme-based assay. In addition, six common drugs were also chosen to study the 3CL^pro^ inhibition. Among these compounds, lapatinib showed high efficiency of 3CL^pro^ inhibition (IC_50_ value of 35 ± 1 μM and K_i_ of 23 ± 1 μM). The binding behavior of lapatinib against 3CL^pro^ was elucidated by molecular dynamics simulations. This drug could well bind with 3CL^pro^ residues in the five subsites S1’, S1, S2, S3, and S4. Moreover, lapatinib’s key chemical pharmacophore features toward SAR-CoV-2 3CL^pro^ shared important HBD and HBA with potent peptidomimetic inhibitors. The rational design of lapatinib was subsequently carried out using the obtained results. Our discovery provides an effective repurposed drug and its newly designed analogs to inhibit SARS-CoV-2 3CL^pro^.

## Introduction

The coronavirus disease 2019 (COVID-19) has become pandemic [1, 2] and has been spreading rapidly around the world [3]. This highly contagious virus is caused by coronaviruses responsible for the severe acute respiratory syndrome (SARS). Remarkably, infected people with COVID-19 can be asymptomatic or symptomatic with a high fever, difficulty in breathing, pneumonia, and multi-organ failure, which can be fatal [4–6]. This leads to strong motivation for global computational and experimental researchers to develop anti-SARS agents. The SARS-CoV-2 chymotrypsin-like cysteine protease (3CL^pro^), also called the main protease (M^pro^), has become a potential therapeutic target for antiviral therapy due to its critical role in viral replication and infection process [7]. The virus’s life cycle begins with the virus’s spike protein attaching to the ACE2 receptor on host cells. The viral envelope fuses with the host cell membrane, and the viral DNA is released into the cytoplasm. The viral genome (+ssRNA) is translated into a large polypeptide (PP) chain. The newly formed PP chain is autoproteolytically cleaved by 3CL^pro^ encoded by the viral genome, to produce several non-structural proteins (NSPs) necessary for viral replication. 3CL^pro^ cleaves the PP chain into 11 NSPs making this protease one of the major targets for drug development against SARS-CoV-2. The 3CL^pro^ is active in a homodimer form consisting of the A and B protomers [8]. The monomeric structure reveals three domains: I (residues 8–101) and II (residues 102–184) are mainly β-barrels, while domain III contains α-helices (residues 201–306). The active site is the cleft between domains I and II. The H41/C145 catalytic dyad found in the active site of SAR-CoV-2 3CL^pro^ is similar to other 3CL^pro^ in that C145 functions as the nucleophile in the proteolytic process with the recognition sequence Leu-Gln↓Ser-Ala-Gly, where the arrow is the cleavage site [9, 10].

Several inhibitors which target SAR-CoV-2 3CL^pro^ have been developed. The peptidomimetic inhibitors N3 [11], 13b [12], and 11a [13] bind to the 3CL^pro^ active site and interact with the catalytic dyad [14]. Besides, masitinib (IC_50_ of 2.5 μM and K_i_ of 2.6 μM) and boceprevir (IC_50_ of 8.0 μM and antiviral activity in Vero E6 cells with EC_50_ of 15.57 μM) are found to exhibit the 3CL^pro^ activity effectively [15, 16]. For binding patterns at the molecular level, C145, H163, and H164 residues are essential for masitinib binding [15], while H41, G143, C145, H164, and E166 residues are involved for boceprevir binding [16]. Two approved drugs (disulfiram and carmofur) and four clinical trials compounds (ebselen, tideglusib, shikonin, and PX-12) inhibit the SARS-CoV-2 3CL^pro^ with IC_50_ of 0.67–21.4 μM have been reported [11]. Baicalin and baicalein showed potent antiviral activities in the Vero E6 cells with the IC_50_ values of 6.41 ± 0.95 μM and 0.94 ± 0.20 μM, respectively [17]. Some preclinical compounds, GC-376 (IC_50_ of 0.03 μM and EC_50_ of 2.07 μM), calpain inhibitor II (IC_50_ of 0.97 μM and EC_50_ of 0.49 μM), and calpain inhibitor XII (IC_50_ of 0.45 μM and EC_50_ of 3.37 μM) also showed 3CL^pro^ inhibition as well as SARS-CoV-2 antiviral activity [18]. Additionally, Pfizer’s inhibitors PF-00835231 (K_i_ of 0.27 nM) [19] and PF-07304814 (K_i_ of 174 nM) [20] continue to be evaluated in the clinical trials phase 2/3. The PF-00835231 is well stabilized within the 3CL^pro^ active site by forming hydrogen bonds with H41, C145, H164, E166, and Q189 residues [19]. In November 2021, Pfizer announced the SARS-CoV-2 3CL^pro^ inhibitor, paxlovid (PF-07321332; ritonavir), in phase 2/3. In non-hospitalized high-risk adults with COVID-19, this drug was found to lower the probability of hospitalization or death by 89% compared to the placebo studied [21, 22]. This PF-07321332 can inhibit the 3CL^pro^ function by covalently bound to catalytic residue C145 [23]. To enhance the bloodstream levels of paxlovid, it is administered in combination with a low dose of ritonavir as a pharmacokinetic enhancer [21].

Virtual screening is one of the strategies to quickly develop new drugs using the existing database for drug repurposing therapeutics. This strategy has been successfully applied in different diseases, such as hypertensive (captopril and aliskiren), liver cancer (nolatrexed, phase III clinical trial), and glaucoma (dorzolamide); and it allows to discover of new therapeutic agents in a fast way [24]. Likewise, virtual screening based on molecular docking strategy is generally used as this method incorporates protein flexibility. Numerous virtual screening investigations of SAR-CoV-2 3CL^pro^ inhibitors have been reported. For example, binifirate and bamifylline were identified from the SuperDRUG2 database through energy-optimized pharmacophore hypothesis (E-pharmacophore) based virtual screening and Glide docking by using X77 inhibitor as a template [25]. Additionally, the phytochemical compounds retrieved from the PubChem database were screened considering the PHASE screen score, by which six lead compounds, 44256891, 44256921, 102452140, 131751762, 131831710, and 139031086, were obtained [26]. Five natural compounds with pharmacokinetic characteristics (daidzin, phloretin, rosmarinic acid, higenamine hydrochloride, and naringenin chalcone) were screened from the ZINC database using the LUDI-based pharmacophore model of N3, followed by a molecular docking study with MolDock [27]. The small-molecule inhibitors of 3CL^pro,^ including rottlerin (37 μM), amentoflavone (143 μM), and baicalein (208 μM) with IC_50_ in the micromolar range were identified using molecular docking and ligand-based screening [28]. The important residues involved in these compounds binding are E166, T190, and Q189, whereas the catalytic residues H41 and C145 are crucial for amentoflavone and baicalein, respectively. Kuzikov et al. screened 8,702 compounds from the Drugs and Probes database, clinical and preclinical compounds using combined structure-based virtual screening and molecular docking [29]. They found that the thioguanosine (antimetabolite, IC_50_ of 6.3 μM), MG-132 (proteasome inhibitor, IC_50_ of 7.4 μM), bronopol (food biocide, IC_50_ of 0.4 μM), and myricetin (JAK1 inhibitor, IC_50_ of 0.22 μM) can inhibit the 3CL^pro^ activity. In addition, myricetin inhibits the 3CL^pro^ by covalently bound to the catalytic Cys145 residue. Furthermore, telaprevir, a hepatitis C virus (HCV) protease inhibitor, forms hydrogen bonds with H163 and E166 residues at the S1 pocket of SAR-CoV-2 3CL^pro^ with enzyme-based assay (IC_50_ = 11.47 μM) [30].

**Fig 1:**
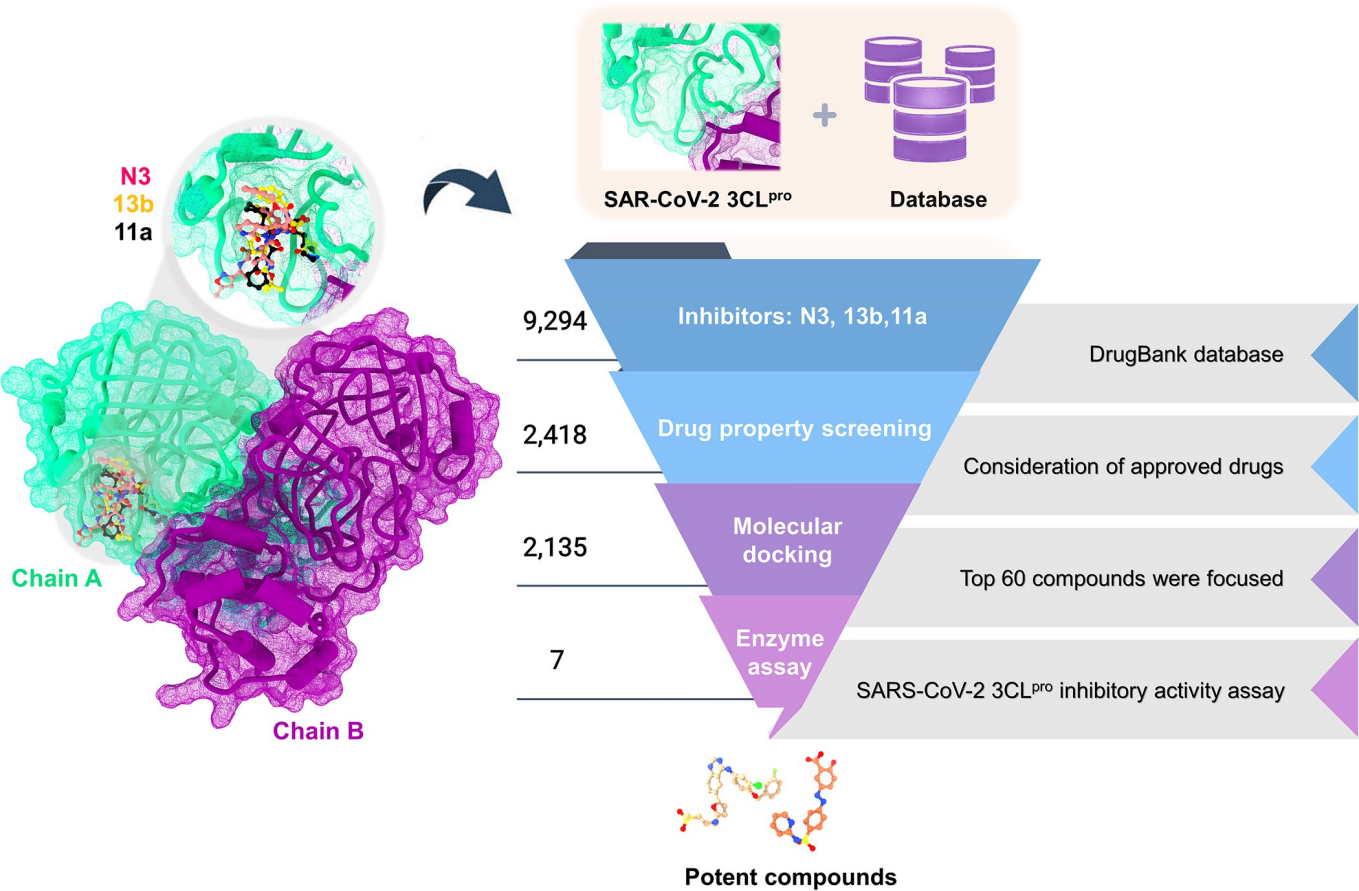
**The virtual screening scheme of molecular docking of SAR-CoV-2 3CL**^**pro**^ **inhibitors from the crystal structures of potent peptidomimetic inhibitors (N3, 13b, and 11a) using the DrugBank database.** The screened compounds were selected, and the common drugs were later included for testing the SAR-CoV-2 3CL^pro^ inhibitory activity by enzyme-based assay.

In this work, the combination of molecular docking of FDA-approved drugs was used to find a new potent anti-SARS-CoV-2 3CL^pro^ (Fig 1) from DrugBank [31]. The three crystal structures of 3CL^pro^ in complex with N3, 13b, and 11a were used as templates. The screened compounds and common drugs were then selected to investigate the 3CL^pro^ inhibition by enzyme-based assay. Finally, the binding patterns, intermolecular interactions, and binding affinities of the most potent compounds with 3CL^pro^ were studied by all-atom molecular dynamics (MD) simulations for 500 ns and the solvated interaction energy (SIE) method. Furthermore, the binding of potent peptidomimetic inhibitors in previous work and the most potent compounds within the active site of SAR-CoV-2 3CL^pro^ derived from MD simulations were investigated using the pharmacophore model. Based on the protein-ligand design, the most potent compound was used as a model to design and improve binding efficiency against 3CL^pro^. The information obtained could be helpful in the development of new anti-SARS-CoV-2 3CL^pro^ drug candidates.

## Materials and methods

### Computational details

#### Molecular docking

The three crystal structures of SARS-CoV-2 3CL^pro^ with the peptidomimetic inhibitors bound, *i*.*e*., N3 (6LU7 [11]), 13b (6Y2F [12]), and 11a (6LZE [13]) were used to find the drugs repurposed for treatment of COVID-19 by molecular docking study. The validation of the docking study was carried out by re-docking all inhibitors (N3, 13b, and 11a) into the substrate-binding cleft of the three SAR-CoV-2 3CL^pro^ structures. The 2,418 FDA-approved drugs from the DrugBank database [31] were considered. Their protonation states were automatically altered by the FlexX software LeadIT version 2.3 [32]. Each known inhibitor N3, 11a, or 13b was selected as the docking center, and the sphere of a 10-Å radius around the ligand was created for docking compounds with 100 docking poses. The resulted docking pose with the lowest binding energy was selected for analysis. The results from docking were visualized by Accelrys Discovery Studio 2.5 [33] and UCSF Chimera 1.15 [34].

#### Molecular Dynamics (MD) simulations

According to the experimental study, the potent inhibitor lapatinib against SARS-CoV-2 3CL^pro^ from the 6LU7 model was investigated by all-atom MD simulations for 500 ns in the periodic boundary condition using AMBER20 [35]. Subsequently, the ligand was optimized at the HF/6–31(d) level of theory using the Gaussian09 program. The ligand’s restrained ESP (RESP) charges converted from the electrostatic potential (ESP) charges were generated using the parmchk module. The protein and ligand were treated with the AMBER ff14SB force field [36] and generalized AMBER force field version 2 (GAFF2) [37], respectively. All missing hydrogen atoms were added using the tleap module and then were minimized by the 1000 iterations of steepest descent (SD) followed by 4,000 iterations of conjugated gradient (CG). The TIP3P model was used to soak the system in the cubic box (12 Å from the protein surface). The water molecules were minimized using the 500 SD iterations followed by 1000 CG iterations, while the remaining system was restrained using a 500 kcal/mol^2^·A^2^ force constant. Subsequently, the whole complex was fully minimized without any restraint using 1000 iterations of SD followed by 2500 iterations of CG.

The short-range cutoff of 12 Å was used to consider non-bonded interactions, whereas Ewald’s method was adopted for long-range electrostatic interactions [38]. The pressure was controlled using the Berendsen method [39]. The SHAKE method was applied to constrain all covalent bonds involving hydrogen atoms [40]. The simulated models were heated to 310 K for a relaxation duration of 100 ps. A Langevin thermostat controlled the temperature with a collision frequency of 2.0 ps. The time step was set as 2 fs [41–44], while the MD trajectories were saved every 10 ps. Finally, a 500-ns unconstrained NPT simulation of lapatinib/SARS-CoV-2 3CL^pro^ complex at 310 K was carried out. The structural dynamics properties, including the distance between the center of mass (C_m_) of the drug and the C_m_ of active site residues, intermolecular hydrogen bonding, the number of contact atoms, root mean square deviation (RMSD), interaction energy including electrostatic and van der Waals interactions, radius of gyration (Rg), principal component analysis (PCA) and root mean square fluctuation (RMSF) of the lapatinib/3CL^pro^ complex were calculated by the CPPTRAJ module [45]. With a set of 100 snapshots derived from the last 100-ns, the protein-ligand binding pattern was characterized by the MM-GBSA per-residue decomposition free energy (${\Delta G}_{bind}$) calculation with the MMPBSA.py [46], while the binding free energy of the complex was predicted by solvated interaction energy (SIE) approach [47] implemented in AMBER20.

#### Protein-ligand pharmacophores

The MD trajectories at equilibrium state (2,000 frames) of lapatinib/SARS-CoV-2 3CL^pro^ complex from the last 100 ns (401–500 ns) and the SARS-CoV-2 3CL^pro^ complexes with three known inhibitors (N3, 11a, and 13b) from the last 20 ns (81–100 ns) in our previous work [14] were used to create pharmacophore features using LigandScout 4.4.2 program combined with the KNIME analysis platform [48, 49]. Note that all solvated waters and counterions were removed from MD trajectories. First, the information on complex structure and trajectory were loaded into the “PDB reader” and “DCD trajectory reader”, respectively. The pharmacophore features between inhibitor(s)/SARS-CoV-2 3CL^pro^ were then generated using the "Pharmacophore creator” node in the KNIME program with default parameters. Subsequently, the obtained pharmacophore models were clustered and aligned by chemical features using “Pharmacophore clustering”. The similar pharmacophore models of each system were removed, and subsequently, unique pharmacophore models were clustered to a representative pharmacophore model (RPMs). These RPMs in each system were attained from the “Pharmacophore writer” node.

### Experimental details

#### 3CL^pro^ inhibition assay

The activity assay for 3CL^pro^ was carried out as previously described [50]. SARS-CoV-2 3CL^pro^ was expressed and purified using a method as previously reported for SARS-CoV-1 3CL^pro^ [51]. 3CL^pro^ was used at 0.2 μM for all experiments. Enzymatic activity was measured as the initial rate of cleavage of the fluorogenic substrate E(EDANS)TSAVLQSGFRK(DABCYL), which measured the excitation and emission wavelength at 340 and 490 nm, respectively. For the initial screen of inhibitory activity, enzymatic activity was measured in the presence and absence of a 100 μM inhibitor. The initial rate in the absence of an inhibitor was used for normalization. For IC_50_ determination, the initial rate of substrate (25 μM) cleavage was measured when lapatinib was present at various concentrations. The IC_50_ value was fitted with GraphPad Prism 8. The K_i_ value was calculated using the Cheng-Prusoff equation [52] with the previously reported K_m_ value (51 μM) [50].

## Results and discussion

### Virtual screening

**Fig 2:**
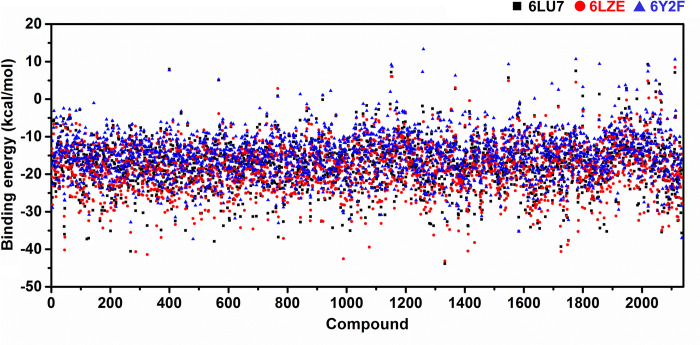
Binding energy (kcal/mol) of approved drugs against three X-ray structures of SARS-CoV-2 3CL^pro^ (PDB entry codes: 6LU7, 6LZE, and 6Y2F) derived from molecular docking.

The strategy of therapeutic repurposing is currently widely employed to find possible COVID-19 treatments. The practice of repurposing drugs reduces the cost, time, and risk of drug development. To search for repurposing drugs against SARS-CoV-2 3CL^pro^, molecular docking was applied on the 2,418 approved drugs from 9,294 Drugbank compounds [31] using the three X-ray structures of this enzyme in complex with the peptidomimetic inhibitors N3, 13b, and 11a initially reported for SARS-CoV-2 3CL^pro^ in according to our previous work [14]. Only 2,135 compounds were docked successfully into the binding pocket of 3CL^pro^, while their binding energies were plotted and compared in Fig 2 and S1 Table. The predicted binding affinity of screened compounds showed a consistency phenomenon in all three protein structures. Note that some screened compounds against SARS-CoV-2 3CL^pro^ from our studies, such as masitinib (DB11526), conivaptan (DB00872), imatinib (DB00619), flupenthixol (DB00875), pentoxyverine (DB11186), and boceprevir (DB08873), were found to inhibit SARS-CoV-2 infection in A549 human lung cells and also 3CL^pro^ activity [15, 30, 53].

**Fig 3:**
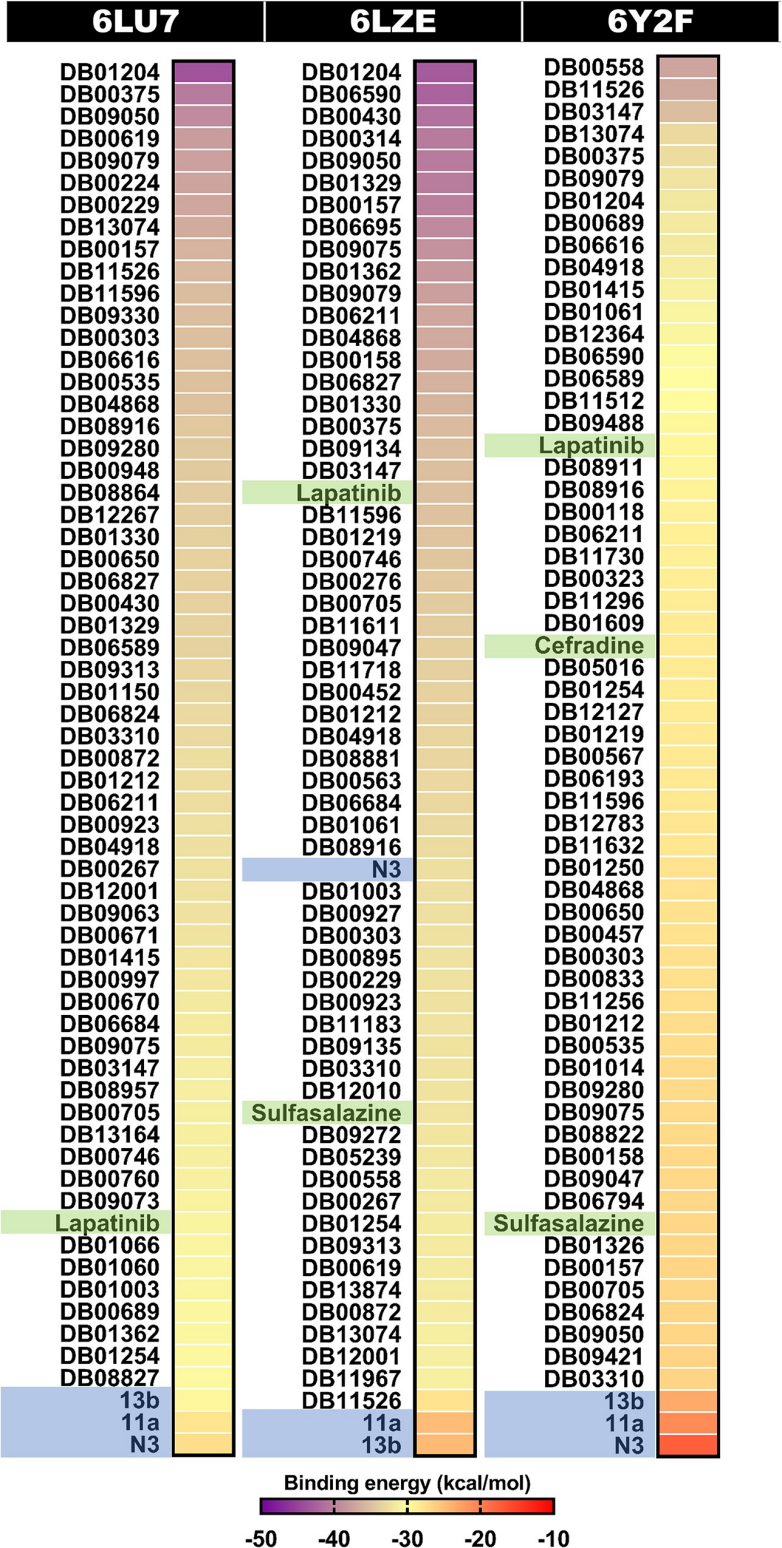
Binding energy heatmap (kcal/mol) of top 60 screened compounds against three X-ray structures of SARS-CoV-2 3CL^pro^ (PDB entry codes: 6LU7, 6LZE, and 6Y2F) resulted from molecular docking. The compounds in the blue and green highlight are the reference compounds used for screening and the selected compounds for enzyme-based assay, respectively.

**Fig 4:**
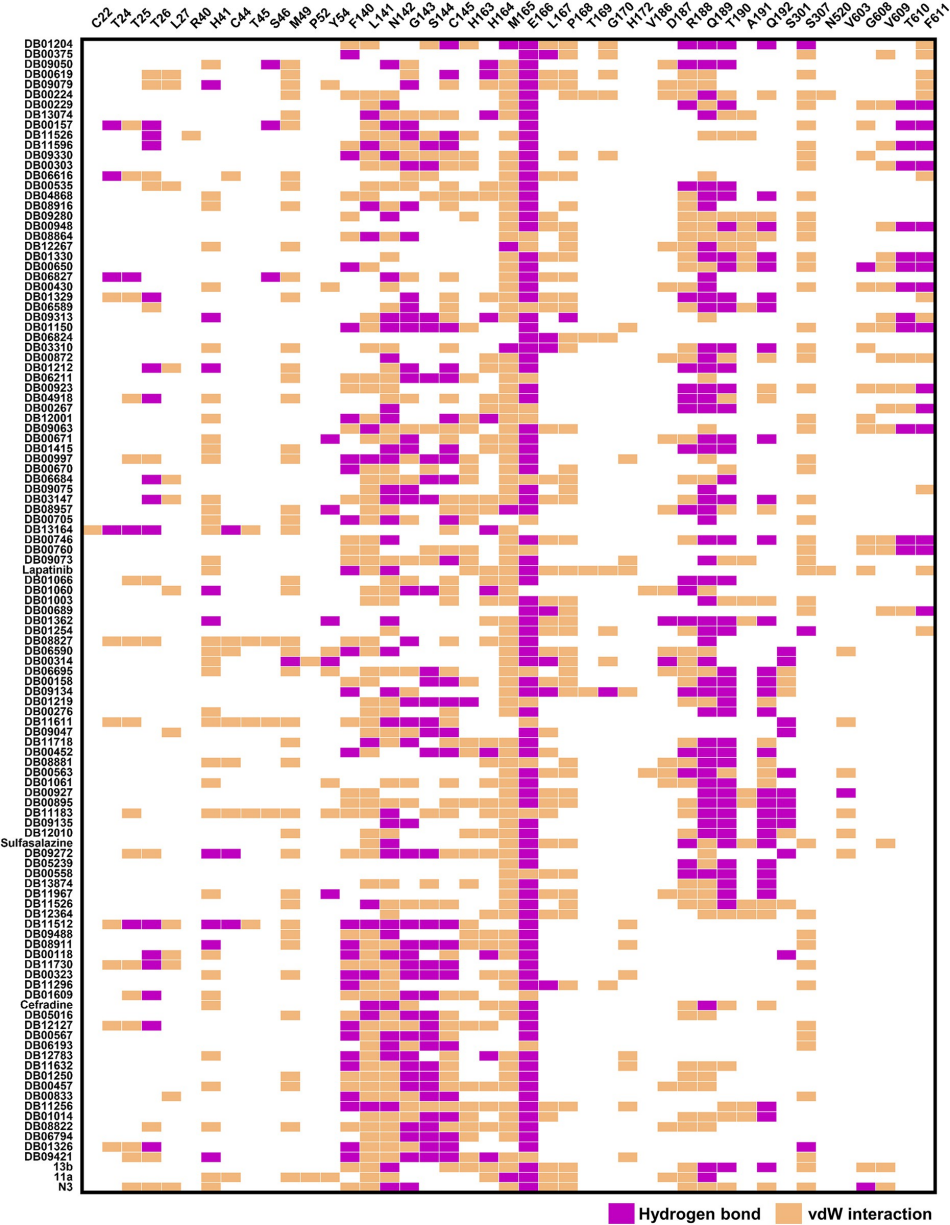
Heat map of the top compounds interacting with SARS-CoV-2 3CL^pro^ derived from Fig 3 relative to the known inhibitors derived from LigPlot version 2.2.

By considering the binding energy relative to its template, the top 60 compounds ranked with reference compounds or known inhibitors (N3, 13b, and 11a) are shown in Fig 3. The binding energies of the top 60 compounds were in the range of -30.53 to -43.85 kcal/mol for the 6LU7 model, -27.81 to -43.14 kcal/mol for the 6LZE model, and -26.64 to -37.30 kcal/mol for the 6Y2F model. The key binding residues of these compounds against the SARS-CoV-2 3CL^pro^ compared to the three inhibitors are shown in Fig 4. It was found that most screened drugs interacted with the residues H41, M49, L141, C145, M165, L167, P168, and R188 via van der Waals (vdW) interaction and formed hydrogen bonds with N142, G143, E166, Q189 residues. Since some compounds are not readily commercially available, a randomly available selection of compounds with higher binding affinity than the reference compounds from the three docking results was made further to investigate the SARS-CoV-2 3CL^pro^ inhibition *in vitro* enzyme-based assay. Our selected drugs/inhibitors and descriptions are summarized in Table 1. These were an anti-cancer agent (lapatinib), anti-inflammatory drug (sulfasalazine), and antibiotic (cefradine). In addition, common drugs include two anti-cancer agents (AZD-7762, and GSK-690693), diuretics drug ((S)-indapamide), coenzyme ((6S)-5,6,7,8-tetrahydrofolic acid), and two HIV-1 protease inhibitors (ritonavir and lopinavir) were also used to perform SARS-CoV-2 3CL^pro^ inhibition.

**Table 1:** The selected compounds and common drugs for SAR-CoV-2 3CL^pro^ inhibitory activity assay.

Name	Drug activity
**Selected compounds**	
Lapatinib (DB01259)	• Breast cancer • Lung cancer
Cefradine (DB01333)	• A semi-synthetic cephalosporin antibiotic
Sulfasalazine (DB00795)	• Treatment of inflammatory bowel diseases
**Common drugs**	
AZD-7762 (DB12242)	• Treatment of cancer, solid tumors, and advanced solid malignancies.
GSK-690693 (DB12745)	• Treatment of tumor, cancer, and lymphoma
(S)-Indapamide (DB07467)	• Diuretics
(6S)-5,6,7,8-tetrahydrofolic acid (DB02031)	• Parent compound of a variety of coenzymes that serve as carriers of one-carbon groups in metabolic reactions
Ritonavir (DB00503)	• HIV protease inhibitor
Lopinavir (DB01601)	• HIV-1 protease inhibitor used in combination with ritonavir to treat HIV infection

### *In vitro* testing for 3CL^pro^ inhibition

**Fig 5:**
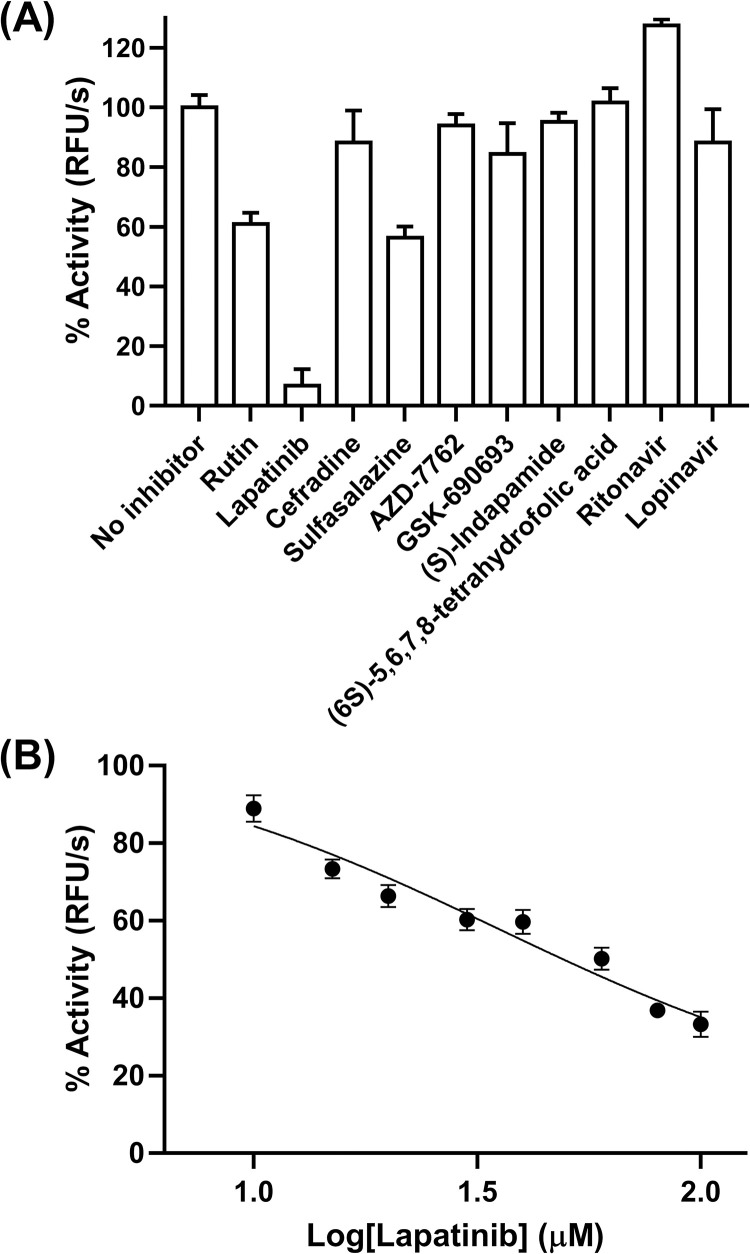
*In vitro* enzymatic studies. (A) Relative activity of 3CL^pro^ in the presence of 100 μM of compounds and (B) inhibition of 3CL^pro^ by lapatinib at various concentrations.

The compounds (100 μM) were screened for 3CL^pro^ inhibitory activity using rutin at the same concentration as a positive control [50] (Fig 5A). For selected compounds, cefradine did not show inhibitory activity against 3CL^pro^, while sulfasalazine inhibited 3CL^pro^ to a similar range to rutin. The AZD-7762, GSK-690693, (S)-indapamide, and (6S)-5,6,7,8-tetrahydrofolic acid from common drugs did not inhibit the 3CL^pro^. In addition, ritonavir and lopinavir also did not show inhibitory activity as previously reported [54]. Co-administration of ritonavir helps to slow down PF-07321332 metabolism by cytochrome enzymes, providing for increased circulating concentrations of the main drug has been reported [55]. Intriguingly among tested compounds, lapatinib almost wholly abolishes the 3CL^pro^ activity at 100 μM. Thus, lapatinib was further investigated (Fig 5B). The IC_50_ value of lapatinib was 35 ± 1 μM. Furthermore, the calculated inhibitory (K_i_) constant value was 23 ± 1 μM. Lapatinib was previously used to treat SARS-CoV-2 infected Vero cells [56] and A549 human lung cells [15]. It was found that lapatinib can inhibit SARS-CoV-2 infected cell viability with the value of 31.1 μM for Vero cells [56] and 1.6 μM for A549 cells [15]. Our results suggested that lapatinib could inhibit 3CL^pro^, resulting in a reduction of viral replication. Moreover, the selected compounds (lapatinib, cefradine, and sulfasalazine) and common drugs (AZD-7762, GSK-690693, (S)-indapamide, (6S)-5,6,7,8-tetrahydrofolic, ritonavir, and lopinavir) interacted with the substrate-binding residue M165 of SARS-CoV-2 3CL^pro^ via vdW interaction in correspondence to rutin binding (S1 Fig). Hydrogen bonds with the residues N142 and E166 stabilized the binding of lapatinib, cefradine, and sulfasalazine.

### Mechanism of action of lapatinib

**Fig 6:**
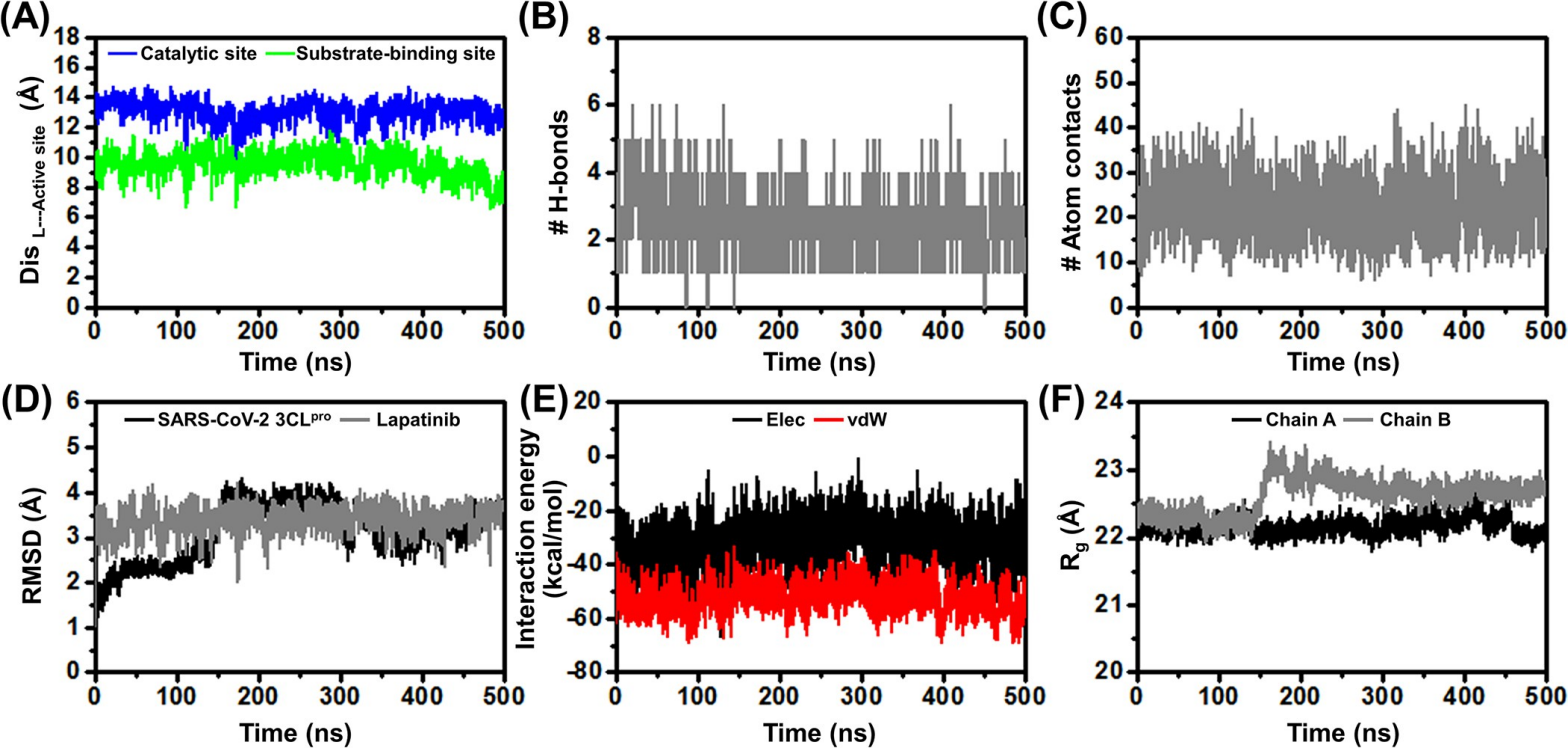
Dynamics analysis of lapatinib with SARS-CoV-2 3CL^pro^. (A) Distance between the C_m_ of lapatinib and the C_m_ of SARS-CoV-2 3CL^pro^ active site residues, (B) # H-bonds, (C) # atom contacts, (D) RMSD plot for protein backbone (CA, C, O, and N atoms) and lapatinib, (E) interaction energy, and (F) radius of gyration (R_g_) of 3CL^pro^ in each chain plotted along with the 500-ns MD simulation.

**Fig 7:**
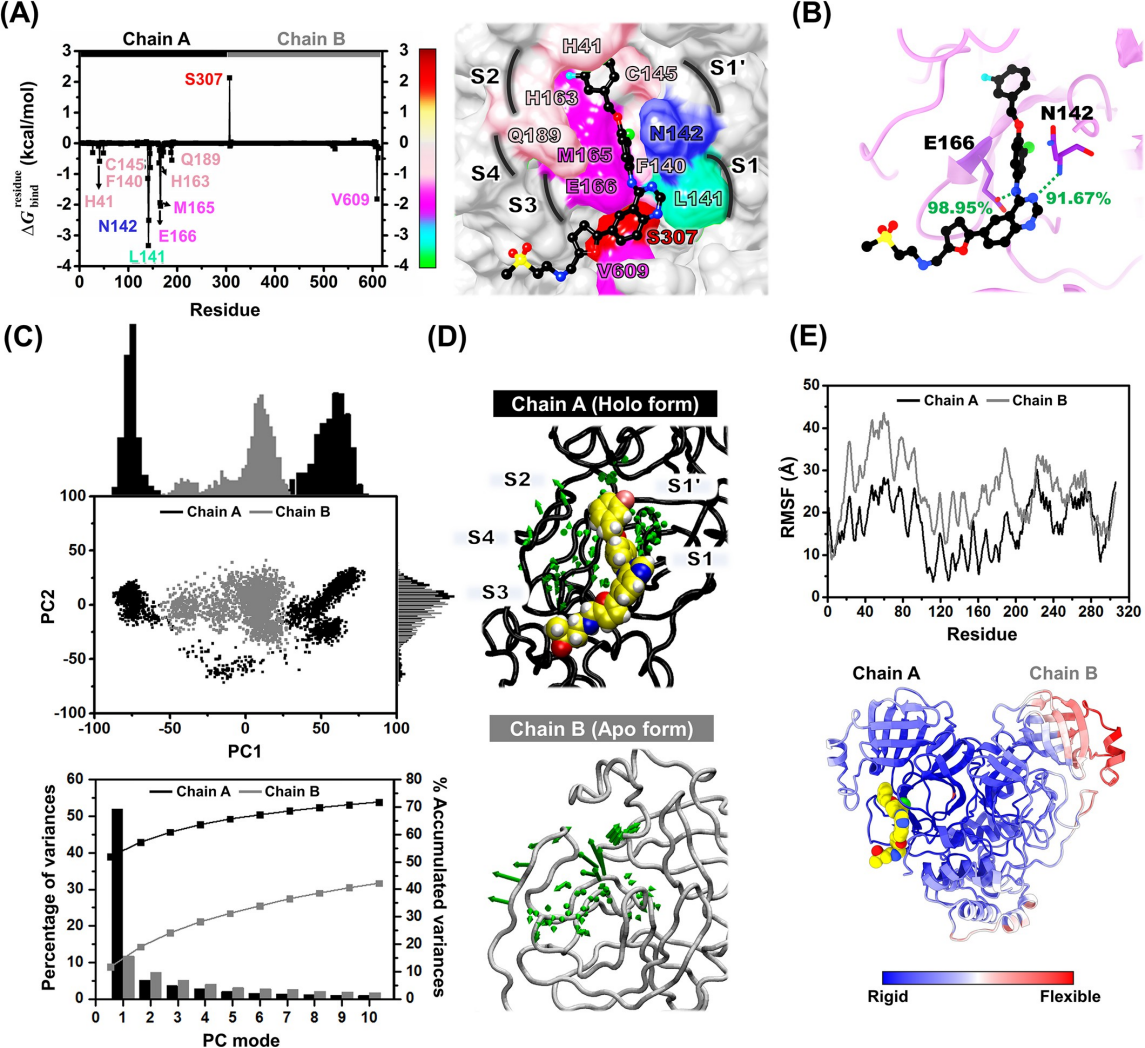
Key binding residues and protein motions of SARS-CoV-2 3CL^pro^/lapatinib complex. (A) Binding free energy contribution per residue (${\Delta G}_{bind}^{residue}$) for lapatinib binding derived from the last 100 ns, colored from dark red to green according to the highest to lowest free energies. The residues with ${\Delta G}_{bind}^{residue}$ ≤ −0.5 kcal/mol and ≥ 0.5 kcal/mol are labeled. The key residues are colored according to their ${\Delta G}_{bind}^{residue}$ values. The representative structure was taken from the last MD snapshot. Noted that the quinazoline scaffold of lapatinib highly interacted with L141 at the S1 site (${\Delta G}_{bind}^{residue}$ of -3.33 kcal/mol). Their percentages of hydrogen bond occupation are shown in (B). (C) 2D projection of MD trajectories on the first two PC modes and PCA scree plot, (D) PC1 porcupine plot of the holo and apo forms, where the arrowhead and length represent the direction and amplitude of motion, respectively, and (E) RMSF plot of 3CL^pro^ in each chain.

To investigate how lapatinib showed potential SAR-CoV-2 3CL^pro^ inhibition at the molecular level, the binding of this repurposed drug at the 3CL^pro^ active site was investigated by 500-ns MD simulation (Fig 6). The distance between the center of mass (C_m_) of the drug and the C_m_ of active site residues, number of intermolecular H-bonds (# Hbonds), and number of atom contacts (# atom contacts) with the drug molecule along the simulation time was analyzed and plotted in Figs 6A–6C. It was found that the distance between the C_m_ of lapatinib and the C_m_ of active site residues (S2 Fig) was relatively stable. In addition, the #Hbonds (2.43 ± 0.80) and #atom contacts (22.07 ± 6.03) of the lapatinib within the binding site were detected along with the simulation. Furthermore, the RMSD analysis of SAR-CoV-2 3CL^pro^ and lapatinib was also performed (Fig 6D). Lapatinib was quite stable at the active site from the beginning to the end of the simulation, supported by the MD snapshots of the lapatinib/SARS-CoV-2 3CL^pro^ complex along with the simulation (S3 Fig). In Fig 6E, the vdW interaction (-52.28 ± 5.36 kcal/mol) seems to be more crucial than electrostatic (Elec) interaction (-32.24 ± 7.96 kcal/mol). In addition, the radius of gyration (R_g_, Fig 6F) of the protein in chain A with lapatinib binding was more compact than the protein in chain B (without lapatinib bound). In this study, the last 100 ns (from 401 to 500 ns) of the simulation was extracted to investigate the essential binding residues for lapatinib binding, using MM/GBSA per-residue decomposition energy calculation. The residue contributions in terms of ${\Delta G}_{bind}^{residue}$ are plotted in Fig 7A.

Lapatinib was likely stabilized by the SAR-CoV-2 3CL^pro^ residues in the five essential pockets: (i) N142 and C145 residues at S1’ site, (ii) F140 and L141 residues at the S1 site, (iii) H41 and H163 residues at the S2 site, (iv) M165 and E166 residues at the S3 site, and (v) Q189 residue at the S4 site. The 3CL^pro^ subsites S1, S2, and S4 are shaped into well-formed binding pockets, whereas S1ʹ and S3 are located on the protein surface with no defined shape (9). In addition, the quinazoline scaffold at the P1’ position (S4 Fig) hydrophobically interacted with V609 at the C-terminal of chain B, while this drug was destabilized by S307. The chlorophenol ring at the P2’ position and the quinazoline scaffold at the P1’ position highly interacted with L141 and N142 (${\Delta G}_{bind}^{residue}$ of -3.33 and -2.51 kcal/mol). In contrast, a previous report showed little interactions from MD simulations of these residues with the peptidomimetic inhibitors 13b and 11a (14). In addition, the 1-(3-fluorobenzyloxy)-2-chlorobenzene at P2’ position was feasibly inserted into the S3 pocket, resulting in strong occupancy within the binding site by showing the high binding with M165 and E166 (${\Delta G}_{bind}^{residue}$ of -1.93 and -2.05 kcal/mol). In correspondence with previous reports [14, 16, 57], these residues provided high hydrophobic interactions with N3, 13b, and 11a, boceprevir, and telaprevir. Importantly, lapatinib inhibits 3CL^pro^ activity by interacting with the catalytic dyad H41 and C145 (${\Delta G}_{bind}^{residue}$ of -0.58 and -0.79 kcal/mol). Both residues play a vital role in the hydrolytic process, in which C145 functions as a nucleophile and H41 acts as a base catalyst. The partial negative charge produced at the substrate peptide bond is stabilized by an oxyanion hole formed by the backbone of C145 (18). The inhibition by interaction with the catalytic residues are commonly found in SAR-CoV-2 3CL^pro^ inhibitors, for example, PF-07304814 and PF-07321332, which are currently in phase 1 and 2/3 clinical trials, respectively [21, 58], masitinib [53], baicalein [59], and rutin [50]. In addition, hydrogen bond formation is essential for biological systems. The hydrogen bond occupation in Fig 7B demonstrates that the quinazoline scaffold at the P1’ position forms the hydrogen bonds with the N142 residue (N^3^H—N142@H) at 91.67%. The amine group in P1’ positions of lapatinib showed a strong hydrogen bond with E166 residue (N^4^H—E166@OE) at 98.95%, in agreement with the peptidomimetic inhibitors N3, 13b, and 11a from MD study [14]; and other inhibitors PF-07321332 [21], boceprevir [16], herbacetin and morin [60] in previous reports. Moreover, the last 100 ns trajectories were used to study the protein motion by PCA and RMSF analysis. The first ten PC mode values revealed the accumulated variances of chain A (holo form) and chain B (apo form) in Fig 7C. With a higher distribution in 2D projection on the first PC, lapatinib binding at the active site of SAR-CoV-2 3CL^pro^ could enhance the percentage of variances of PC1 from 11.70% in chain B to 52.01% in chain A. This finding supported how the active site in chain B flipped away to the upper site with a high amplitude (Fig 7D), resulting in the open conformation. In chain A, the active site conformation changed to accommodate and stabilize the lapatinib binding, *i*.*e*., the mobility of the active site residues was relatively lower. The reduction of protein motion upon the ligand binding was supported by RMSF analysis (Fig 7E).

The binding affinity of the lapatinib/SAR-CoV-2 3CL^pro^ complex was predicted by the solvated interaction energy (SIE) approach, using the 100 snapshots of the last 100-ns. It was found that the vdW interaction (-57.47 ± 0.49 kcal/mol) plays an important role for molecular complexation rather than Elec interaction (-13.61 ± 0.30 kcal/mol). The vdW interaction was the main force found in this work, consistent with other previously reported inhibitors, such as PF-07321332 [21], bamifylline [61], saquinavir, aclarubicin, and GRL-142 [62]. Although the binding affinity of lapatinib (-9.20 ± 0.06 kcal/mol) is to some extent overestimated in comparison with experimental data (${\Delta G}_{exp}$ of -6.32 kcal/mol converted from IC_50_ value), it is in the same range as the peptidomimetic inhibitors (${\Delta G}_{bind}$ of -9.92, -9.68, and -10.35 kcal/mol for N3, 11a, and 13b) [14]. Our finding suggests that lapatinib has the potential to be used to combat COVID-19.

### Pharmacophore models of potent inhibitors

**Fig 8:**
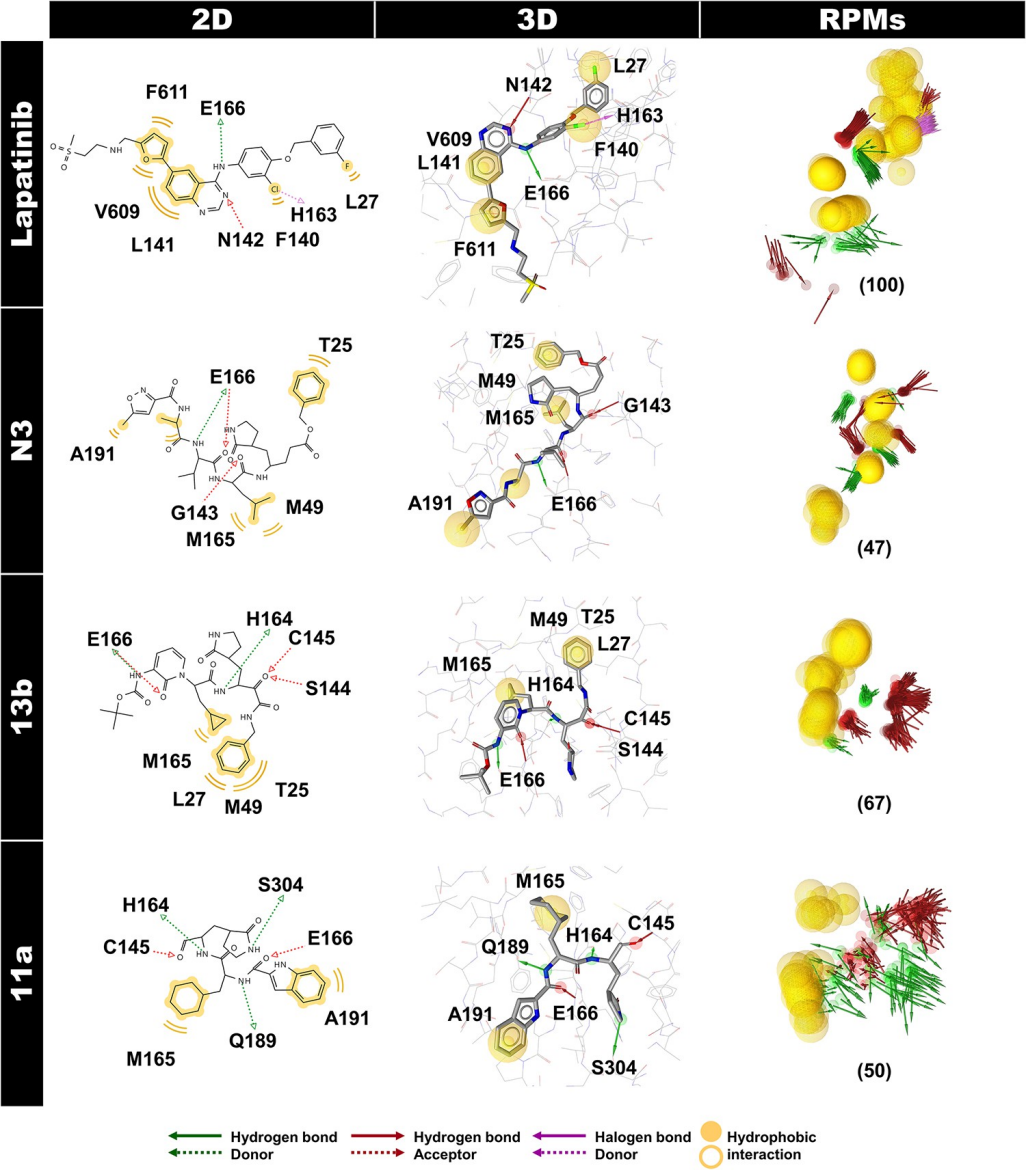
The pharmacophore models of lapatinib and peptidomimetic inhibitor(s) in complex with SAR-CoV-2 3CL^pro^. The 2D and 3D pharmacophore models of the first representative frame of lapatinib at 401 ns, and peptidomimetic inhibitors N3, 13b, and 11a at 81 ns, where RPMs derived from MD trajectories of lapatinib-3CL^pro^ complex (401–500 ns), and peptidomimetic inhibitor(s)-3CL^pro^ complex (81–100 ns) are illustrated on the right column. The green arrow, red arrow, purple arrow, and yellow color sphere (or circle in 2D) are pharmacophore features of hydrogen bond donor (HBD) and acceptor (HBA), halogen bond donor (XBD), and hydrophobic interaction properties, respectively.

**Fig 9:**
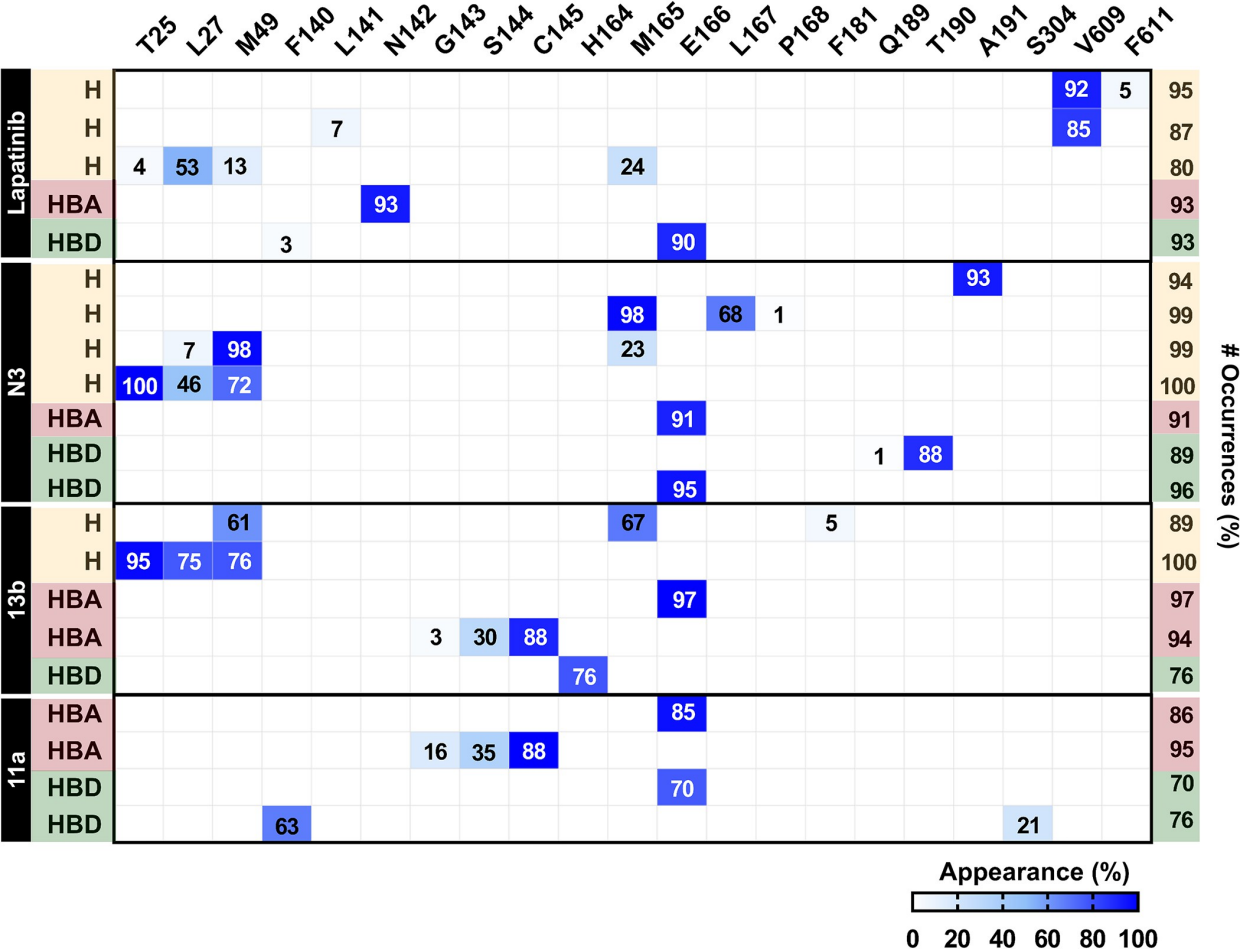
**Interaction map of four inhibitors against SAR-CoV-2 3CL**^**pro**^ **derived from 401–500 ns MD trajectories for lapatinib 81–100 ns, and MD trajectories for peptidomimetic inhibitors N3, 13b, and 11a.** The abbreviations of H, HBA, and HBD represent the pharmacophore features of hydrophobic interaction, hydrogen bond acceptor, and hydrogen bond donor properties. The numbers in the blue box are the percentage of appearance in each interaction per residue.

Pharmacophore models are a set of steric and electronic features common to a series of active compounds with a specific biological target. The hydrogen bond donor (HBD), hydrogen bond acceptor (HBA) abilities, positively and negatively charged groups, and hydrophobic and aromatic regions are typical features [63–65]. In this work, the structure-based pharmacophore, which is specialized to detect ligand-protein interactions [66], was applied to the MD trajectories of the repurposing drug lapatinib within SAR-CoV-2 3CL^pro^. The 2D and 3D pharmacophore models of the first representative frame of lapatinib at 401 ns, and peptidomimetic inhibitors (N3, 13b, and 11a from previous work [14]) at 81 ns, and RPMs derived from MD trajectories of lapatinib-3CL^pro^ complex (401–500 ns) and peptidomimetic inhibitor(s)-3CL^pro^ complex (81–100 ns) are depicted in Fig 8. In addition, the ratio of pharmacophore occurrences with > 70% is shown and further discussed in Fig 9.

Hydrogen bonding, HBD or HBA, and hydrophobic interactions were the critical chemical pharmacophore features of all inhibitors binding to SAR-CoV-2 3CL^pro^, while the halogen bond donor (XBD) feature was only detected in the lapatinib system. There were 100, 47, 67, and 50 RPMs for lapatinib, N3, 13b, and 11a, respectively. In the case of the repurposing drug, lapatinib showed hydrophobic interactions with T25, L27, M49, and M165, similar to the N3 and 13b. This drug revealed a high appearance of hydrogen bonding with the substrate-binding residues N142 (93%) and E166 (90%) and high hydrophobic interaction with V609 (92% and 85%) in chain B of 3CL^pro^. The strong hydrogen bonding with E166 residue was also observed in the three peptidomimetic inhibitors (N3, 13b, and 11a) and the previously reported potent inhibitor X77 against SAR-CoV-2 3CL^pro^ (6W63.pdb) [67]. In contrast, a hydrogen bond with N142 was found only for the lapatinib system. Interestingly, lapatinib could form XBD with H163 substrate-binding residue (66%).

For peptidomimetic inhibitors, the chemical pharmacophores of N3 consisted of HBD with E166, Q189, and T190, HBA with E166, and hydrophobic interactions with T25, M49, M165, and A191, in correspondence to the previous reports of pharmacophore generated from the co-crystal structure (6LU7.pdb) [27, 68]. Similar to N3, the 13b also had HBA with E166 and hydrophobic interactions with T25, L27, M49, and M165. Other key features of 13b were HBD with H164 and HBA with G143, S144, and C145. In 11a, a high HBD was found with E166, and there were two HBAs with C145 and E166. No strong hydrophobic interaction of 11a in the active site was obtained. Moreover, both N3 and 11a could bind to the active site comparable to 13b by binding to the E166 substrate-binding residue at the P3 site corresponding to the crystal structures [11–13]. The H41 and C145 catalytic dyad residues are located between domains I and II in the cleft. The residue C145 functions as a nucleophile in the first step of the hydrolysis process, assisted by the catalytic base H41 [10]. Inhibitors or drugs that can interact with these residues will inhibit the activity of SAR-CoV-2 3CL^pro^. The 13b and 11a showed a high HBA appearance with the catalytic residue C145 (88%); however, no hydrophobic interaction of 11a was detected. Therefore, among peptidomimetic inhibitors (N3, 13b, and 11a), 13b showed the highest binding efficiency against SAR-CoV-2 3CL^pro^ as described previously [14]. Pharmacophore modeling is widely used in virtual screening to identify compounds with the desired biological effect [69]. The pharmacophore models obtained from these four systems can be further utilized for antiviral drug screening to combat COVID-19 infection disease.

### Rational drug design against SAR-CoV-2 3CL^pro^

**Fig 10:**
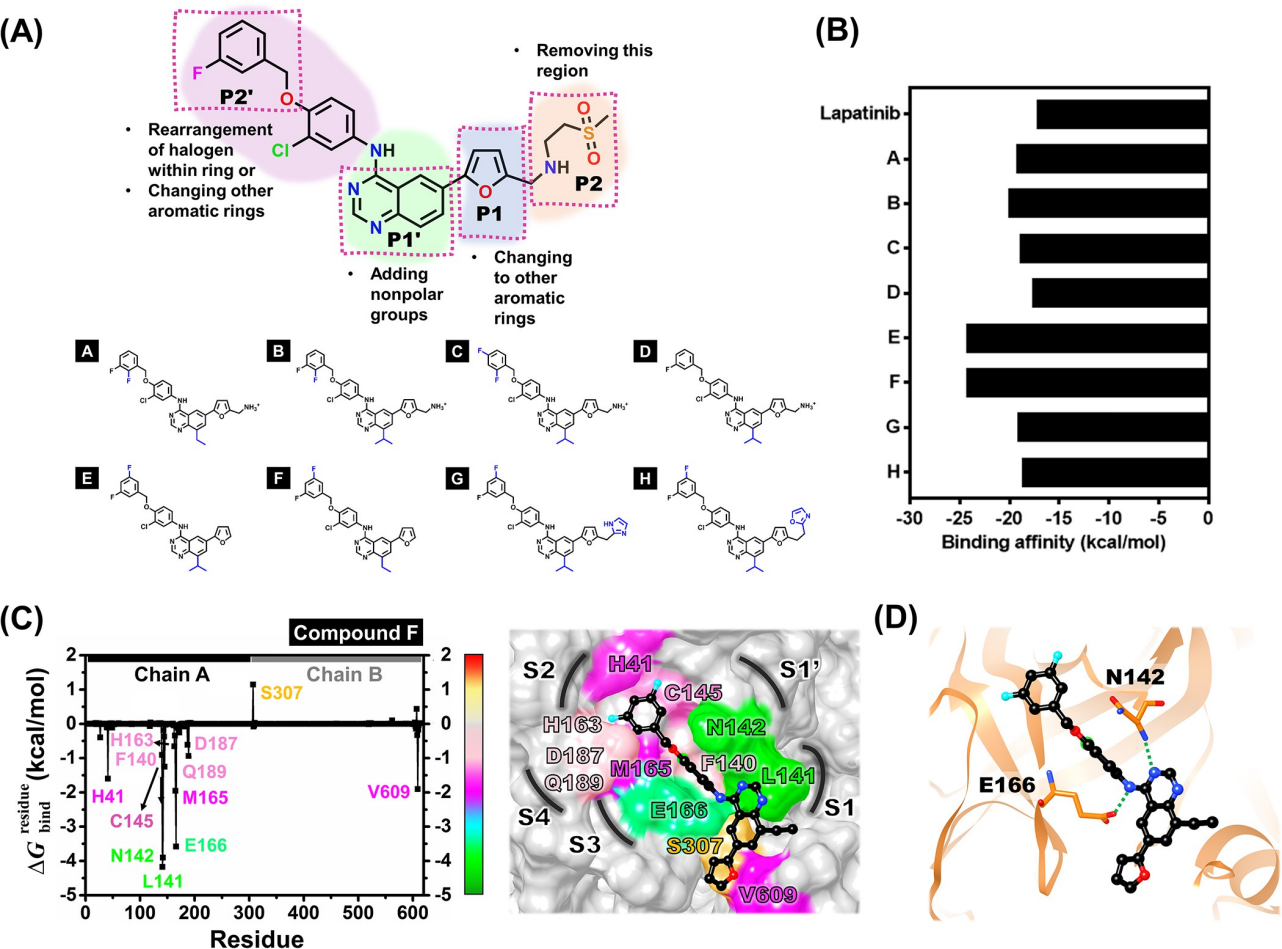
Rational design of lapatinib against the SARS-CoV-2 3CL^pro^. (A) 2D structure of lapatinib and designed lapatinibs, (B) predicted binding affinity of the designed compounds A-H against SARS-CoV-2 3CL^pro^ in comparison with lapatinib using LigandScout 4.4.2 program, (C) the binding free energy per residue of modified lapatinib, compound F/SARS-CoV-2 3CL^pro^ complex, and its hydrogen bond interactions (D). The results were obtained from one snapshot of the complex after system minimization.

The rational drug design based on lapatinib structure derived from MD simulation and pharmacophore model results was conducted to enhance the ligand-binding ability toward SAR-CoV-2 3CL^pro^. Some functional groups of lapatinib should be modified as given in Fig 10A: (i) rearrangement of halogen within the aromatic ring (e.g., *ortho*, *meta*, or *para* position) or changing other types of the aromatic ring (e.g., aniline or pyridine) in the P2’ site to increase the hydrophobic interaction with catalytic residues H41 and C145, (ii) enhancing the nonpolar moieties (e.g., methyl, ethyl or propyl group) at the quinazoline core of lapatinib in the P1’ site, which interacts with L141 and V609 residues, and (iii) changing the furan ring in the P1 site (e.g., imidazole, pyrrole, or oxazole) to increase the hydrophobic interaction with V609 residue. However, the core quinazoline (P1’ site) and benzene ring with Cl atom side chain (P2’ site), which forms hydrogen bond and halogen bond interactions, should be retained (Figs 5E and 6). Besides, the (methylsulfonyl)ethanamine in the P2 site located at the solvent-exposed region can be removed (Fig 7A and 7B).

The representative structure (one snapshot) of lapatinib derived from the last 100 ns MD simulation was used as a model for modification. The lapatinib derivative(s)/SAR-CoV-2 3CL^pro^ complex was structurally minimized based on the MMFF94 force field by LigandScout 4.4.2 program. Subsequently, these complexes’ binding affinities were obtained and compared to lapatinib. We found that the binding affinities of the newly designed compounds A to H showed binding (−17.51 to -24.22 kcal/mol) with 3CL^pro^ stronger than lapatinib (−17.09 kcal/mol) (Fig 10B). Among novel lapatinib derivatives, the compounds E (−24.22 kcal/mol) and F (−24.21 kcal/mol) gave binding affinities against 3CL^pro^ at a similar level. Compound F was selected to study the binding pattern by MM/GBSA decomposition free energy calculation (Fig 10C). The hydrophobic interactions of compound F with catalytic residues H41 (-1.60 kcal/mol, deep pink), and C145 (-1.25 kcal/mol, light pink), and L141 (-4.17 kcal/mol, green) were significantly enhanced as proposed. Furthermore, hydrogen bond formation of the quinazoline core (N atom) with both residues N142 and E166 remained (Fig 10D).

The physicochemical properties and drug-likeness properties (S2 Table) of lapatinib and potent designed lapatinibs, compounds E and F were predicted using the SwissADME (www.swissadme.ch/) [70]. The obtained result revealed that lapatinib was not acceptable for Lipinski [71] and Ghose [72] rules in similar to compounds E and F. Lapatinib complies with the criteria of the MDDR’s rule [73], while both potent designed lapatinibs are found to accept Veber’s [74] rule. Additionally, this drug and potent designed lapatinibs were not classified as Pan-assay interference compounds (PAINS) [75]. Therefore, these derivatives could be further developed as SARS-CoV-2 3CL^pro^ inhibitors.

## Conclusions

In this work, the combination of virtual screening based on molecular docking and experimental testing of repurposed drugs was successfully applied to discover SAR-CoV-2 3CL^pro^ inhibitors. The 2,135 compounds were obtained from *in silico* screening. Subsequently, three screened compounds and four common drugs were selected to test for 3CL^pro^ inhibition. Among these compounds, lapatinib showed the highest 3CL^pro^ inhibition with the IC_50_ and inhibitory (K_i_) constant of 35 μM and 23 μM, respectively. In addition, our results revealed how lapatinib inhibits 3CL^pro^ at the molecular level by molecular dynamic simulations. The binding affinity of lapatinib against 3CL^pro^ was predicted by SIE calculation, showing a good agreement with the IC_50_ value. The van der Waals interactions were the major contributor to lapatinib binding to 3CL^pro^. The residues in five pockets of 3CL^pro^ that are important for lapatinib binding include (i) N142 and C145 residues at S1’ site, (ii) F140 and L141 residues at the S1 site, (iii) H41 and H163 residues at the S2 site, (iv) M165 and E166 residues at the S3 site, and (v) Q189 residue at the S4 site. In addition, this drug was also stabilized by hydrogen bond formations with N142 and E166 residues. The critical chemical pharmacophore features of lapatinib binding within SAR-CoV-2 3CL^pro^ were found to be HBD, HBA, XBD, and hydrophobic interactions. Lapatinib’s rational design was also performed. To improve lapatinib binding ability with S1’ and S2 sites of 3CL^pro^, the halogen inside the aromatic ring (*meta* position) at the P2’ site of the drug molecule should be rearranged. Enlarging the nonpolar moiety (ethyl or propyl group) in the quinazoline core of lapatinib should be enhanced hydrophobic interactions with the S1 site. Our theoretical findings will lead to the syntheses of a series of new compounds together with experimental testing in the future. These findings suggest that a combination process of *in silico* screening and experimental studies are beneficial for identifying candidate drugs for the development of potent SAR-CoV-2 3CL^pro^ inhibitors.

## Supporting information

S1 Fig Heat map of the selected compounds and common drugs interacting with SARS-CoV-2 3CL^pro^ in the 6LU7 model relative to rutin derived from LigPlot version 2.2. (TIF)

S2 Fig 3D structure SARS-CoV-2 3CL^pro^ active site residues including the catalytic site residues (H41 and C145) and the substrate-binding site residues (M49, G143, S144, H163, H164, M165, E166, L167, D187, R188, Q189, T190, A191, and Q192). (TIF)

S3 Fig MD snapshots of lapatinib/SARS-CoV-2 3CL^pro^ complex with the time interval of 10 ns derived from the 500 ns of simulation. (TIF)

S4 Fig 2D structure of lapatinib. (TIF)

S1 Table List of 2,135 FDA-approved drugs from DrugBank database and binding energies against three X-ray structures of SARS-CoV-2 3CL^pro^. (DOCX)

S2 Table Physicochemical property and drug-likeness predictions of lapatinib and its designed analogs. (DOCX)

S1 Graphical abstract (TIF)
