# Supplementary figures and images for: Identification of repurposing therapeutics toward SARS-CoV-2 main protease by virtual screening
Source: PLoS One. 2022 Jun 30;17(6):e0269563. doi: 10.1371/journal.pone.0269563 (PMC9246117; doi:10.1371/journal.pone.0269563)

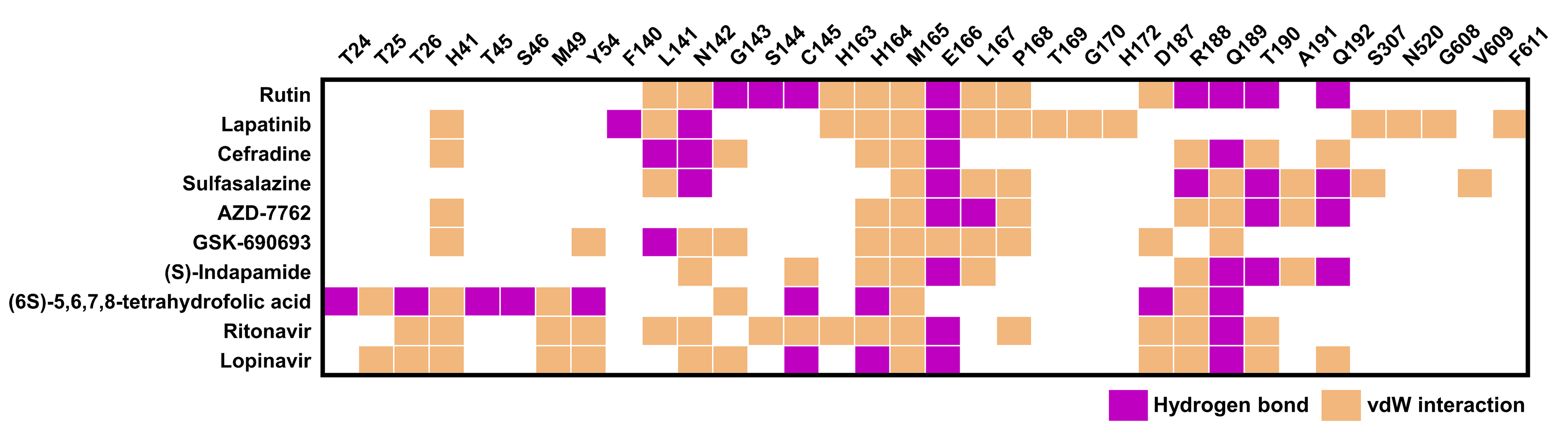

Supplement: S1 Fig — (TIF) [file pone.0269563.s001.tif]

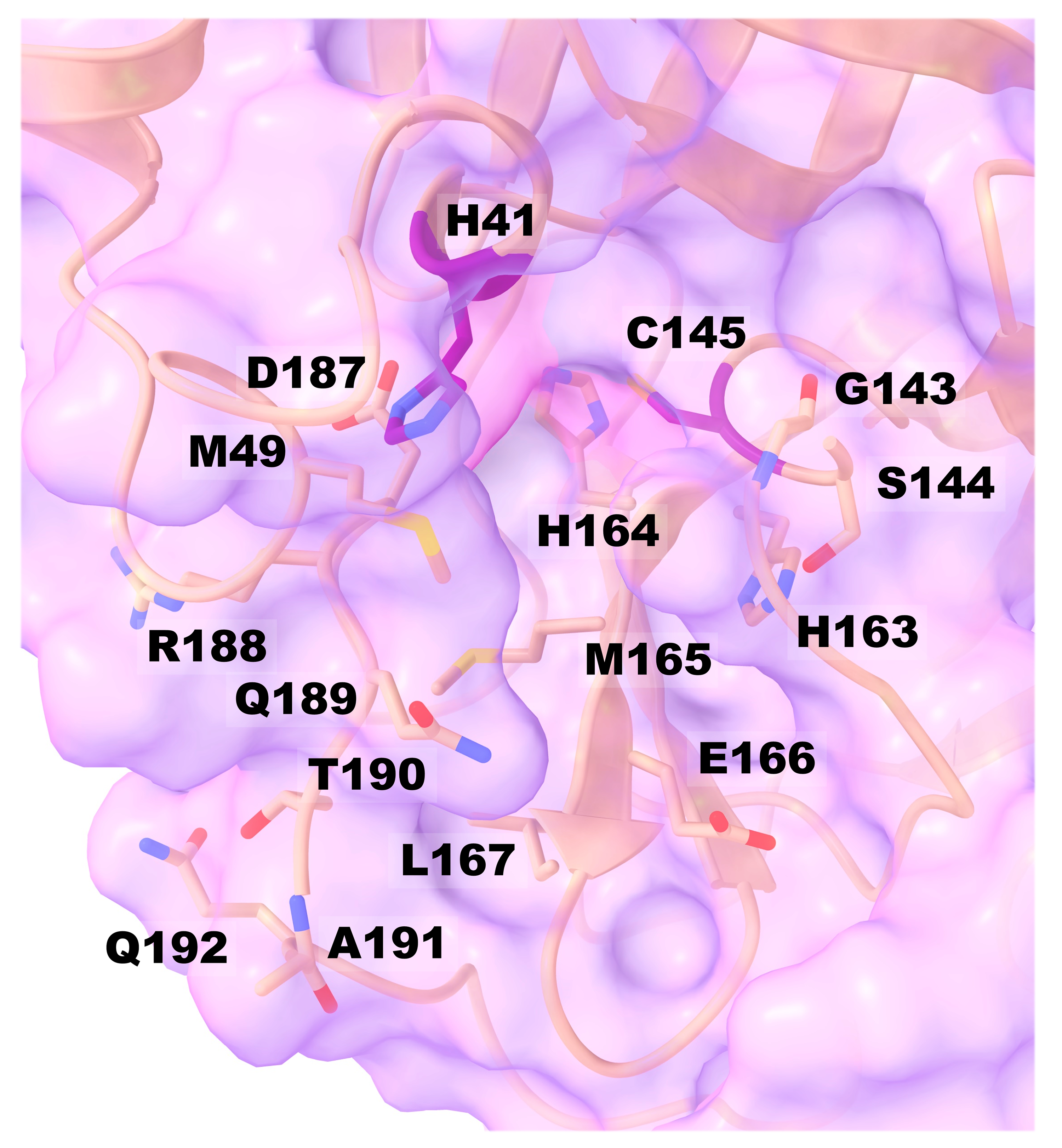

Supplement: S2 Fig — (TIF) [file pone.0269563.s002.tif]

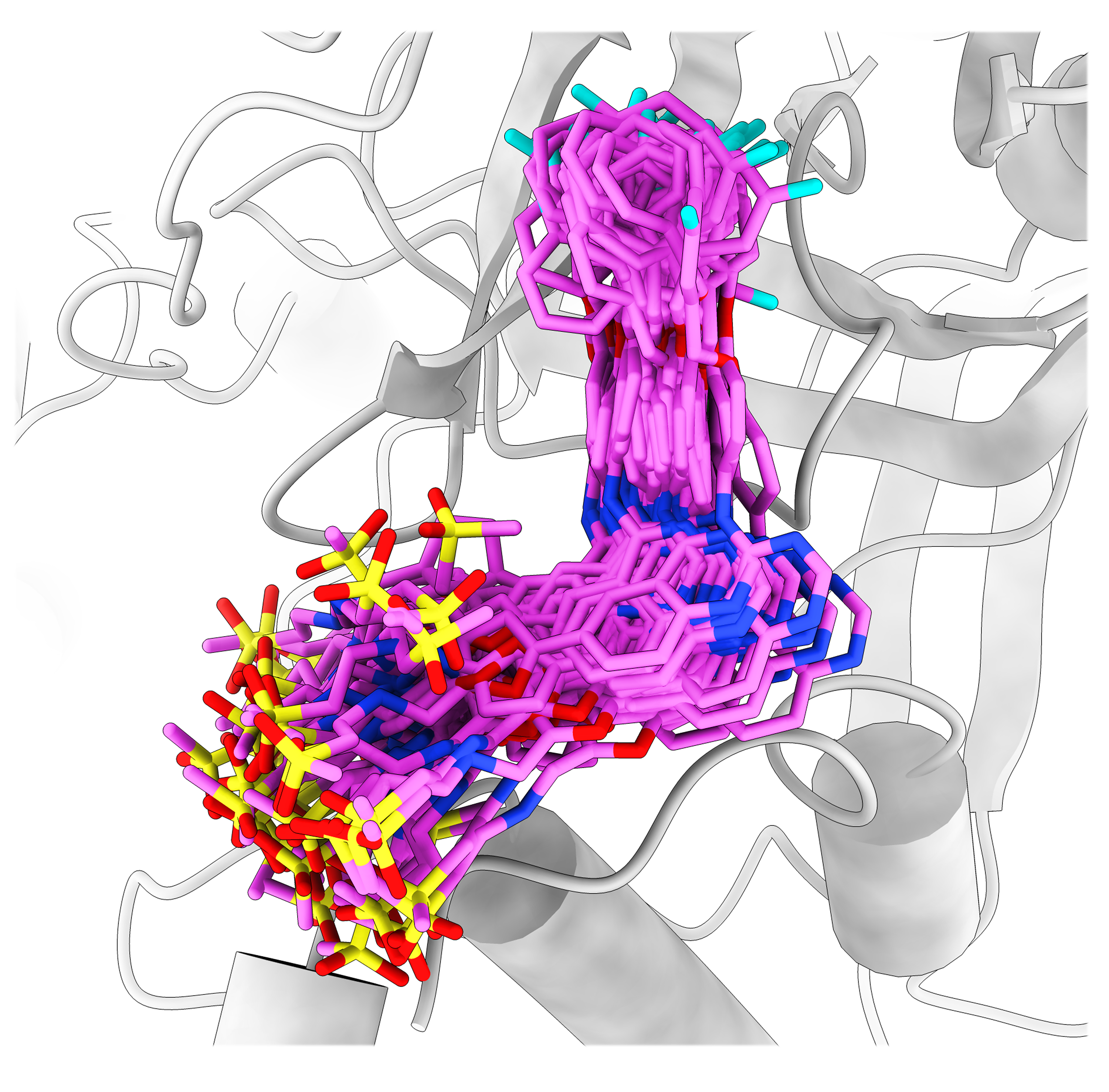

Supplement: S3 Fig — (TIF) [file pone.0269563.s003.tif]

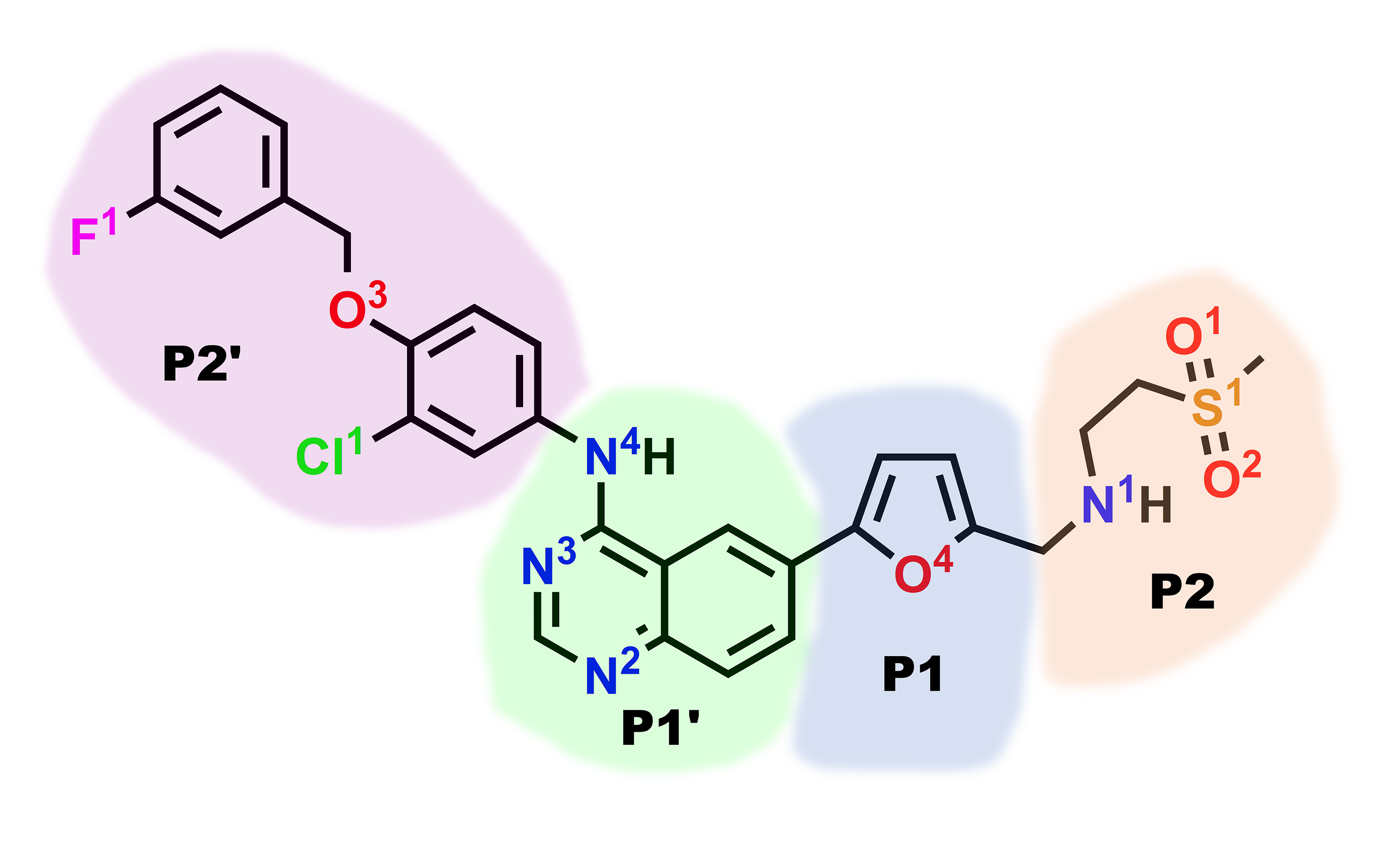

Supplement: S4 Fig — (TIF) [file pone.0269563.s004.tif]

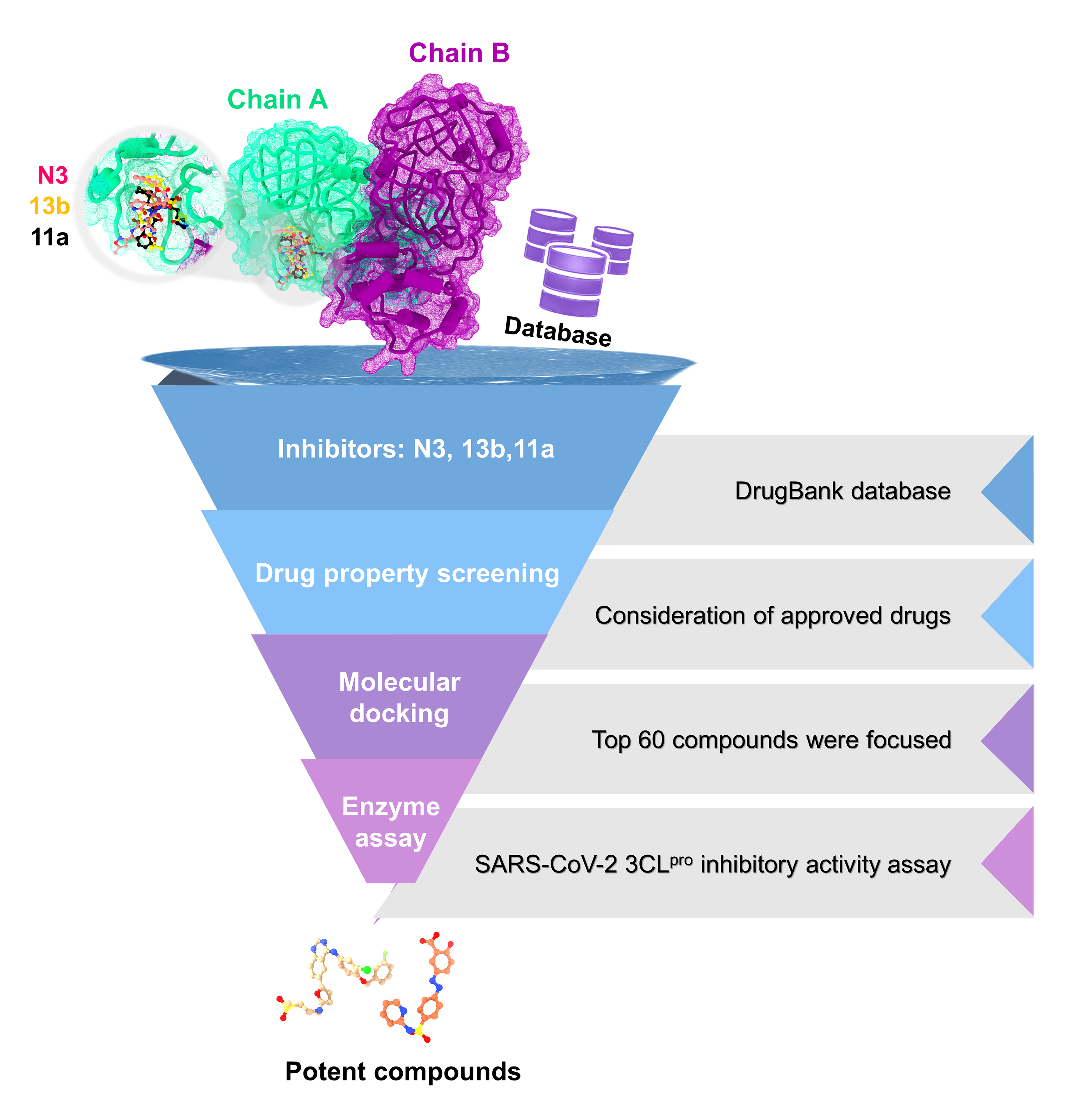

Supplement: S1 Graphical abstract — (TIF) [file pone.0269563.s007.tif]
