# Supplementary material for: Identification of repurposing therapeutics toward SARS-CoV-2 main protease by virtual screening
Source: PLoS One. 2022 Jun 30;17(6):e0269563. doi: 10.1371/journal.pone.0269563 (PMC9246117; doi:10.1371/journal.pone.0269563)
Supplement: S1 Table — (DOCX) [file pone.0269563.s005.docx]

S1 Table. List of 2,135 FDA-approved drugs from DrugBank database and binding energies against three X-ray structures of SARS-CoV-2 3CL^pro^.

| Compound no. | Compound ID | **Binding energy (kcal/mol)** | | |
| --- | --- | --- | --- | --- |
|  |  | **6LU7** | **6LZE** | **6Y2F** |
| 1 | DB00114 | -17.07 | -18.42 | -16.86 |
| 2 | DB00117 | -22.14 | -20.71 | -17.29 |
| 3 | DB00119 | -11.15 | -13.25 | -11.03 |
| 4 | DB00118 | -26.21 | -28.15 | -28.95 |
| 5 | DB00120 | -14.46 | -14.34 | -15.33 |
| 6 | DB00121 | -16.45 | -23.08 | -22.63 |
| 7 | DB00123 | -22.44 | -14.68 | -15.15 |
| 8 | DB04519 | -5.87 | -9.26 | -7.60 |
| 9 | DB00122 | -5.53 | -6.69 | -3.15 |
| 10 | DB00125 | -17.91 | -20.99 | -17.48 |
| 11 | DB00126 | -15.61 | -18.82 | -16.62 |
| 12 | DB00127 | -23.18 | -19.12 | -14.69 |
| 13 | DB00128 | -14.99 | -18.65 | -14.27 |
| 14 | DB00129 | -22.55 | -17.08 | -18.22 |
| 15 | DB00130 | -20.10 | -19.41 | -16.50 |
| 16 | DB00131 | -24.68 | -26.03 | -21.47 |
| 17 | DB00132 | -6.57 | -7.50 | -4.91 |
| 18 | DB00133 | -21.00 | -16.57 | -14.21 |
| 19 | DB00134 | -12.92 | -12.82 | -12.91 |
| 20 | DB00135 | -19.45 | -18.01 | -17.93 |
| 21 | DB12243 | -12.11 | -15.77 | -11.61 |
| 22 | DB00136 | -12.29 | -16.89 | -6.60 |
| 23 | DB00138 | -21.58 | -19.50 | -17.97 |
| 24 | DB00139 | -11.31 | -10.20 | -10.39 |
| 25 | DB00141 | -17.10 | -23.77 | -13.64 |
| 26 | DB00140 | -21.73 | -22.14 | -21.58 |
| 27 | DB00142 | -22.28 | -20.84 | -14.71 |
| 28 | DB00143 | -23.89 | -23.24 | -23.32 |
| 29 | DB00145 | -13.93 | -13.61 | -12.21 |
| 30 | DB00144 | -10.01 | -15.11 | -12.96 |
| 31 | DB00147 | -18.10 | -15.83 | -16.70 |
| 32 | DB04540 | -7.10 | -11.43 | -4.61 |
| 33 | DB00148 | -16.20 | -20.19 | -15.61 |
| 34 | DB00146 | -11.96 | -17.70 | -8.69 |
| 35 | DB00149 | -13.67 | -13.58 | -12.47 |
| 36 | DB00150 | -18.32 | -20.49 | -14.23 |
| 37 | DB00151 | -18.89 | -15.54 | -15.04 |
| 38 | DB00152 | -13.70 | -15.12 | -14.38 |
| 39 | DB00153 | -8.50 | -14.11 | -5.94 |
| 40 | DB04552 | -19.39 | -23.27 | -21.54 |
| 41 | DB00155 | -19.39 | -21.72 | -20.31 |
| 42 | DB00154 | -5.22 | -6.73 | -2.46 |
| 43 | DB00156 | -20.48 | -17.70 | -14.12 |
| 44 | DB12267 | -33.96 | -31.23 | -25.29 |
| 45 | DB00157 | -36.05 | -40.21 | -26.84 |
| 46 | DB00158 | -29.15 | -36.81 | -27.03 |
| 47 | DB00160 | -15.62 | -14.01 | -12.70 |
| 48 | DB00161 | -14.65 | -13.19 | -12.62 |
| 49 | DB00159 | -7.53 | -7.71 | -6.54 |
| 50 | DB00165 | -16.93 | -14.87 | -14.12 |
| 51 | DB00162 | -12.68 | -15.38 | -9.58 |
| 52 | DB04564 | -14.85 | -18.19 | -13.11 |
| 53 | DB12278 | -16.69 | -11.89 | -12.34 |
| 54 | DB00167 | -11.54 | -13.66 | -10.90 |
| 55 | DB00166 | -7.64 | -12.39 | -9.53 |
| 56 | DB00169 | -8.02 | -13.74 | -5.42 |
| 57 | DB00168 | -21.16 | -23.36 | -20.80 |
| 58 | DB00170 | -16.40 | -13.82 | -12.30 |
| 59 | DB00173 | -18.78 | -20.75 | -16.70 |
| 60 | DB04571 | -15.29 | -15.38 | -14.46 |
| 61 | DB00172 | -14.34 | -18.14 | -15.46 |
| 62 | DB04572 | -4.92 | -4.19 | -2.71 |
| 63 | DB00174 | -17.32 | -18.59 | -15.96 |
| 64 | DB04570 | -26.06 | -25.72 | -23.01 |
| 65 | DB00163 | -5.21 | -8.52 | -4.82 |
| 66 | DB04573 | -15.83 | -21.00 | -16.13 |
| 67 | DB12293 | -18.60 | -13.78 | -12.41 |
| 68 | DB00175 | -13.62 | -17.81 | -12.74 |
| 69 | DB04574 | -18.09 | -21.91 | -13.60 |
| 70 | DB00176 | -12.54 | -19.34 | -6.81 |
| 71 | DB04576 | -22.08 | -26.04 | -22.41 |
| 72 | DB00179 | -19.95 | -17.56 | -16.99 |
| 73 | DB00177 | -20.37 | -20.02 | -22.86 |
| 74 | DB04575 | -11.78 | -12.25 | -9.60 |
| 75 | DB00181 | -12.60 | -15.09 | -15.70 |
| 76 | DB12300 | -16.04 | -15.58 | -14.32 |
| 77 | DB00178 | -16.48 | -20.43 | -16.05 |
| 78 | DB00182 | -11.50 | -11.84 | -11.41 |
| 79 | DB00180 | -20.87 | -20.25 | -13.35 |
| 80 | DB00184 | -15.07 | -15.78 | -10.56 |
| 81 | DB00185 | -11.90 | -11.36 | -11.99 |
| 82 | DB00186 | -16.41 | -26.11 | -16.34 |
| 83 | DB00187 | -15.95 | -20.29 | -10.47 |
| 84 | DB00189 | -8.34 | -6.46 | -3.08 |
| 85 | DB00188 | -23.28 | -22.21 | -21.76 |
| 86 | DB00191 | -11.92 | -10.47 | -9.79 |
| 87 | DB00190 | -17.02 | -24.98 | -15.97 |
| 88 | DB00192 | -17.65 | -16.68 | -15.58 |
| 89 | DB00193 | -13.31 | -12.67 | -11.98 |
| 90 | DB12313 | -22.98 | -23.46 | -14.29 |
| 91 | DB00194 | -22.85 | -23.03 | -17.69 |
| 92 | DB00195 | -14.95 | -16.61 | -8.85 |
| 93 | DB00196 | -15.81 | -17.93 | -10.40 |
| 94 | DB00183 | -26.95 | -30.28 | -22.59 |
| 95 | DB00198 | -13.88 | -16.08 | -16.43 |
| 96 | DB00201 | -12.19 | -11.68 | -8.50 |
| 97 | DB00202 | -2.85 | -6.86 | -2.66 |
| 98 | DB00203 | -21.46 | -24.26 | -18.91 |
| 99 | DB00204 | -27.66 | -29.80 | -23.47 |
| 100 | DB00205 | -20.04 | -18.75 | -15.87 |
| 101 | DB12328 | -17.50 | -20.16 | -13.90 |
| 102 | DB12332 | -23.90 | -26.94 | -22.19 |
| 103 | DB00208 | -12.19 | -13.57 | -11.13 |
| 104 | DB00209 | -13.81 | -10.86 | -15.89 |
| 105 | DB00210 | -18.41 | -20.04 | -17.38 |
| 106 | DB00211 | -25.55 | -26.94 | -18.64 |
| 107 | DB00206 | -20.41 | -11.83 | -11.94 |
| 108 | DB00213 | -16.71 | -23.99 | -15.82 |
| 109 | DB00214 | -24.80 | -27.88 | -22.26 |
| 110 | DB00215 | -14.72 | -17.66 | -12.82 |
| 111 | DB12343 | -22.56 | -21.44 | -19.09 |
| 112 | DB00216 | -20.29 | -23.47 | -19.77 |
| 113 | DB00217 | -12.38 | -11.26 | -9.21 |
| 114 | DB00218 | -28.57 | -25.48 | -26.13 |
| 115 | DB00212 | -15.66 | -20.48 | -15.15 |
| 116 | DB00219 | -14.91 | -9.12 | -7.62 |
| 117 | DB00221 | -15.11 | -16.16 | -16.99 |
| 118 | DB00222 | -17.03 | -25.28 | -18.87 |
| 119 | DB00220 | -23.16 | -15.96 | -18.59 |
| 120 | DB00223 | -14.88 | -17.20 | -15.94 |
| 121 | DB00224 | -37.31 | -27.26 | -17.99 |
| 122 | DB00226 | -16.86 | -18.56 | -19.17 |
| 123 | DB04626 | -18.11 | -25.80 | -16.21 |
| 124 | DB00227 | -16.74 | -16.09 | -12.12 |
| 125 | DB12362 | -16.78 | -15.61 | -17.25 |
| 126 | DB00230 | -12.90 | -15.36 | -13.54 |
| 127 | DB00229 | -37.07 | -32.41 | -25.08 |
| 128 | DB12364 | -26.53 | -30.00 | -30.80 |
| 129 | DB00231 | -18.20 | -21.02 | -17.26 |
| 130 | DB00233 | -17.80 | -18.60 | -18.32 |
| 131 | DB00232 | -16.49 | -19.78 | -15.60 |
| 132 | DB00235 | -14.67 | -18.04 | -13.10 |
| 133 | DB00234 | -19.22 | -18.94 | -20.19 |
| 134 | DB00237 | -12.19 | -15.01 | -9.67 |
| 135 | DB00239 | -15.70 | -17.77 | -14.88 |
| 136 | DB00238 | -13.45 | -16.28 | -13.88 |
| 137 | DB00241 | -9.25 | -14.56 | -9.53 |
| 138 | DB00242 | -19.61 | -19.06 | -17.92 |
| 139 | DB00240 | -15.82 | -17.36 | -15.40 |
| 140 | DB00244 | -21.51 | -20.91 | -20.53 |
| 141 | DB00243 | -29.62 | -26.81 | -20.55 |
| 142 | DB00245 | -16.52 | -12.07 | -11.63 |
| 143 | DB00246 | -22.84 | -27.18 | -17.04 |
| 144 | DB00207 | -9.57 | -13.57 | -1.04 |
| 145 | DB00249 | -19.09 | -21.38 | -22.20 |
| 146 | DB00250 | -15.51 | -17.36 | -15.40 |
| 147 | DB00247 | -20.81 | -19.54 | -13.12 |
| 148 | DB00252 | -14.02 | -20.35 | -15.85 |
| 149 | DB00251 | -17.61 | -19.92 | -12.52 |
| 150 | DB00248 | -20.01 | -22.10 | -20.70 |
| 151 | DB04657 | -19.52 | -23.27 | -15.97 |
| 152 | DB00255 | -15.20 | -15.99 | -11.55 |
| 153 | DB00253 | -16.89 | -18.14 | -14.53 |
| 154 | DB00257 | -12.49 | -9.63 | -14.40 |
| 155 | DB00258 | -9.17 | -10.12 | -8.92 |
| 156 | DB00259 | -17.55 | -19.98 | -19.34 |
| 157 | DB00260 | -16.09 | -20.51 | -15.72 |
| 158 | DB00261 | -14.60 | -17.85 | -13.30 |
| 159 | DB00262 | -10.53 | -15.34 | -13.13 |
| 160 | DB00263 | -18.15 | -20.82 | -21.19 |
| 161 | DB12401 | -20.33 | -22.70 | -14.99 |
| 162 | DB00265 | -12.07 | -11.81 | -12.43 |
| 163 | DB00266 | -23.42 | -23.23 | -21.96 |
| 164 | DB00264 | -15.07 | -15.10 | -12.74 |
| 165 | DB00267 | -32.46 | -31.77 | -23.77 |
| 166 | DB00268 | -13.62 | -12.88 | -10.43 |
| 167 | DB12407 | -22.32 | -27.84 | -17.42 |
| 168 | DB00271 | -20.27 | -24.85 | -18.90 |
| 169 | DB00272 | -23.05 | -19.42 | -18.27 |
| 170 | DB00270 | -18.53 | -18.90 | -19.22 |
| 171 | DB00274 | -29.66 | -30.55 | -21.20 |
| 172 | DB00273 | -17.77 | -20.36 | -18.03 |
| 173 | DB00275 | -23.64 | -22.66 | -18.99 |
| 174 | DB00277 | -13.72 | -16.70 | -11.67 |
| 175 | DB00276 | -28.81 | -34.34 | -25.63 |
| 176 | DB00279 | -16.38 | -17.13 | -14.17 |
| 177 | DB00280 | -14.65 | -11.66 | -10.24 |
| 178 | DB00281 | -15.92 | -11.53 | -12.63 |
| 179 | DB00282 | -15.21 | -17.61 | -16.51 |
| 180 | DB00283 | -15.68 | -13.33 | -12.14 |
| 181 | DB00278 | -29.71 | -27.65 | -20.77 |
| 182 | DB00285 | -12.02 | -11.72 | -8.96 |
| 183 | DB00287 | -12.29 | -19.80 | -15.66 |
| 184 | DB00284 | -16.53 | -18.24 | -10.87 |
| 185 | DB00289 | -13.71 | -16.00 | -13.71 |
| 186 | DB00254 | -17.27 | -20.54 | -20.89 |
| 187 | DB00288 | -13.01 | -18.87 | -5.40 |
| 188 | DB00291 | -7.04 | -10.95 | -6.89 |
| 189 | DB00292 | -12.27 | -16.50 | -10.58 |
| 190 | DB00293 | -25.75 | -29.93 | -23.99 |
| 191 | DB00295 | -18.05 | -16.13 | -16.44 |
| 192 | DB00294 | -12.51 | -12.77 | -8.03 |
| 193 | DB00296 | -19.40 | -16.13 | -14.26 |
| 194 | DB00298 | -23.19 | -19.91 | -19.14 |
| 195 | DB00299 | -18.03 | -24.06 | -17.65 |
| 196 | DB00300 | -13.30 | -18.80 | -15.45 |
| 197 | DB00297 | -19.24 | -15.31 | -13.87 |
| 198 | DB00301 | -24.36 | -22.79 | -21.92 |
| 199 | DB00302 | -17.58 | -14.04 | -23.44 |
| 200 | DB12434 | -17.62 | -19.64 | -17.43 |
| 201 | DB00303 | -35.15 | -32.46 | -27.71 |
| 202 | DB00305 | -23.97 | -28.95 | -19.84 |
| 203 | DB00306 | -9.73 | -13.39 | -9.28 |
| 204 | DB00307 | -14.85 | -22.79 | -18.41 |
| 205 | DB04711 | -26.45 | -27.55 | -14.12 |
| 206 | DB00308 | -16.36 | -21.34 | -14.11 |
| 207 | DB00304 | -7.74 | -5.81 | -5.80 |
| 208 | DB00310 | -24.12 | -23.57 | -21.96 |
| 209 | DB00312 | -11.64 | -13.32 | -8.55 |
| 210 | DB00313 | -7.04 | -7.57 | -7.78 |
| 211 | DB00256 | -22.97 | -29.60 | -23.87 |
| 212 | DB00315 | -18.49 | -23.03 | -22.15 |
| 213 | DB00316 | -16.89 | -14.73 | -14.78 |
| 214 | DB00317 | -26.99 | -29.67 | -21.77 |
| 215 | DB00318 | -17.31 | -14.00 | -13.77 |
| 216 | DB00319 | -24.01 | -28.42 | -26.00 |
| 217 | DB00321 | -12.44 | -12.89 | -11.82 |
| 218 | DB00322 | -18.33 | -25.37 | -19.14 |
| 219 | DB00323 | -28.05 | -26.72 | -28.72 |
| 220 | DB00320 | -27.53 | -22.12 | -18.36 |
| 221 | DB00324 | -16.14 | -16.21 | -15.11 |
| 222 | DB12473 | -16.11 | -21.55 | -20.69 |
| 223 | DB00326 | -19.24 | -23.14 | -14.78 |
| 224 | DB12474 | -9.16 | -9.03 | -7.03 |
| 225 | DB00328 | -25.59 | -21.17 | -19.30 |
| 226 | DB00330 | -19.02 | -16.56 | -14.84 |
| 227 | DB00327 | -17.96 | -16.89 | -14.38 |
| 228 | DB00333 | -10.44 | -9.89 | -8.95 |
| 229 | DB00332 | -10.72 | -16.87 | -7.87 |
| 230 | DB00335 | -17.91 | -20.93 | -16.78 |
| 231 | DB00336 | -23.51 | -21.98 | -22.07 |
| 232 | DB00334 | -21.04 | -23.00 | -19.46 |
| 233 | DB00338 | -18.36 | -21.61 | -15.13 |
| 234 | DB00339 | -14.82 | -17.87 | -12.10 |
| 235 | DB04743 | -21.30 | -24.48 | -20.86 |
| 236 | DB00340 | -18.81 | -14.73 | -13.61 |
| 237 | DB12483 | -26.41 | -25.47 | -17.78 |
| 238 | DB00341 | -23.18 | -18.09 | -16.34 |
| 239 | DB00342 | -18.61 | -22.42 | -11.12 |
| 240 | DB00343 | -14.43 | -13.15 | -10.41 |
| 241 | DB12492 | -17.97 | -16.65 | -17.92 |
| 242 | DB00344 | -13.97 | -12.13 | -11.40 |
| 243 | DB00345 | -20.31 | -21.06 | -19.68 |
| 244 | DB00346 | -20.99 | -27.70 | -18.35 |
| 245 | DB00347 | -11.31 | -11.30 | -11.27 |
| 246 | DB00348 | -18.68 | -22.28 | -17.67 |
| 247 | DB00349 | -16.55 | -17.52 | -14.24 |
| 248 | DB00350 | -21.02 | -21.82 | -17.45 |
| 249 | DB00351 | -12.77 | -12.66 | -8.98 |
| 250 | DB00352 | -18.11 | -16.91 | -20.78 |
| 251 | DB00353 | -19.78 | -21.32 | -13.54 |
| 252 | DB00354 | -19.62 | -10.96 | -11.46 |
| 253 | DB00355 | -27.44 | -26.21 | -19.17 |
| 254 | DB00356 | -12.34 | -16.30 | -11.87 |
| 255 | DB00357 | -16.44 | -22.44 | -15.61 |
| 256 | DB00358 | -18.02 | -20.46 | -12.68 |
| 257 | DB00359 | -21.07 | -18.85 | -17.18 |
| 258 | DB00360 | -20.40 | -28.49 | -22.37 |
| 259 | DB00363 | -20.14 | -19.39 | -17.87 |
| 260 | DB00365 | -22.79 | -24.37 | -21.31 |
| 261 | DB00366 | -13.74 | -11.93 | -10.52 |
| 262 | DB00368 | -23.57 | -20.09 | -17.72 |
| 263 | DB00369 | -15.86 | -16.56 | -17.04 |
| 264 | DB00367 | -12.14 | -14.62 | -10.44 |
| 265 | DB00371 | -7.15 | -14.10 | -10.79 |
| 266 | DB00370 | -17.72 | -14.10 | -14.13 |
| 267 | DB12529 | -9.76 | -10.32 | -7.55 |
| 268 | DB00373 | -21.77 | -22.95 | -15.14 |
| 269 | DB00375 | -40.57 | -35.53 | -32.82 |
| 270 | DB00376 | -13.97 | -13.33 | -12.39 |
| 271 | DB12532 | -8.19 | -11.84 | -6.91 |
| 272 | DB00377 | -16.47 | -16.70 | -15.83 |
| 273 | DB00374 | -18.42 | -18.33 | -15.29 |
| 274 | DB00379 | -11.06 | -9.88 | -6.66 |
| 275 | DB00380 | -17.06 | -24.09 | -20.10 |
| 276 | DB12537 | -14.54 | -15.78 | -13.06 |
| 277 | DB00381 | -21.78 | -19.55 | -14.37 |
| 278 | DB00384 | -23.35 | -23.91 | -25.19 |
| 279 | DB00383 | -16.68 | -15.61 | -15.30 |
| 280 | DB00387 | -11.87 | -12.69 | -12.15 |
| 281 | DB00388 | -17.51 | -20.35 | -15.64 |
| 282 | DB00389 | -11.39 | -12.76 | -11.55 |
| 283 | DB04794 | -18.81 | -17.35 | -17.09 |
| 284 | DB00391 | -27.81 | -24.63 | -20.56 |
| 285 | DB00392 | -13.27 | -7.18 | -9.53 |
| 286 | DB00314 | -29.74 | -40.66 | -26.27 |
| 287 | DB00393 | -21.86 | -18.98 | -17.47 |
| 288 | DB12554 | -12.42 | -10.16 | -9.22 |
| 289 | DB00395 | -13.37 | -14.15 | -10.71 |
| 290 | DB00396 | -16.07 | -15.86 | -12.83 |
| 291 | DB00397 | -14.15 | -14.31 | -12.27 |
| 292 | DB00394 | -13.33 | -17.03 | -14.09 |
| 293 | DB00399 | -21.14 | -24.37 | -16.60 |
| 294 | DB00385 | -22.54 | -24.22 | -20.70 |
| 295 | DB00401 | -17.54 | -15.22 | -15.04 |
| 296 | DB00398 | -25.49 | -25.09 | -20.17 |
| 297 | DB00402 | -24.63 | -24.27 | -24.37 |
| 298 | DB00400 | -20.54 | -14.90 | -15.50 |
| 299 | DB00405 | -13.82 | -12.38 | -12.00 |
| 300 | DB00404 | -18.42 | -14.63 | -14.82 |
| 301 | DB00406 | -11.08 | -11.68 | -9.41 |
| 302 | DB00409 | -19.86 | -18.00 | -13.79 |
| 303 | DB00411 | -7.62 | -12.24 | -8.36 |
| 304 | DB00408 | -18.76 | -19.62 | -16.93 |
| 305 | DB04815 | -11.83 | -13.54 | -12.12 |
| 306 | DB00410 | -20.11 | -25.42 | -11.04 |
| 307 | DB00413 | -13.38 | -13.04 | -11.65 |
| 308 | DB00412 | -18.70 | -22.09 | -17.97 |
| 309 | DB04816 | -22.58 | -23.52 | -20.43 |
| 310 | DB00414 | -21.88 | -25.57 | -17.91 |
| 311 | DB00415 | -30.29 | -22.51 | -24.60 |
| 312 | DB00417 | -19.45 | -20.66 | -16.55 |
| 313 | DB04817 | -19.72 | -19.94 | -17.97 |
| 314 | DB00418 | -8.77 | -14.14 | -8.31 |
| 315 | DB00420 | -12.56 | -13.80 | -10.61 |
| 316 | DB00419 | -14.72 | -21.48 | -14.04 |
| 317 | DB00422 | -15.71 | -18.45 | -12.49 |
| 318 | DB04824 | -16.35 | -18.12 | -19.38 |
| 319 | DB00423 | -12.79 | -18.34 | -13.87 |
| 320 | DB00425 | -18.22 | -16.02 | -13.82 |
| 321 | DB00424 | -16.38 | -17.63 | -12.79 |
| 322 | DB00426 | -9.97 | -19.12 | -10.98 |
| 323 | DB00427 | -19.96 | -16.63 | -14.68 |
| 324 | DB00428 | -22.16 | -25.68 | -19.17 |
| 325 | DB00430 | -33.73 | -41.40 | -22.69 |
| 326 | DB00429 | -13.62 | -18.69 | -14.08 |
| 327 | DB00432 | -18.13 | -23.31 | -18.88 |
| 328 | DB12598 | -24.60 | -26.61 | -21.03 |
| 329 | DB00433 | -21.85 | -19.61 | -16.52 |
| 330 | DB04835 | -16.99 | -17.44 | -12.69 |
| 331 | DB04837 | -17.69 | -10.56 | -10.96 |
| 332 | DB00421 | -12.74 | -17.24 | -11.33 |
| 333 | DB00437 | -16.45 | -17.12 | -13.66 |
| 334 | DB04840 | -15.08 | -19.21 | -15.39 |
| 335 | DB00436 | -16.94 | -20.17 | -17.39 |
| 336 | DB04841 | -21.27 | -18.12 | -15.41 |
| 337 | DB00438 | -27.24 | -24.31 | -21.52 |
| 338 | DB04838 | -12.65 | -14.06 | -11.98 |
| 339 | DB00440 | -21.28 | -24.01 | -15.43 |
| 340 | DB04843 | -16.78 | -11.88 | -13.47 |
| 341 | DB00439 | -25.70 | -14.18 | -14.52 |
| 342 | DB04839 | -9.73 | -13.51 | -10.56 |
| 343 | DB00441 | -17.76 | -22.41 | -18.19 |
| 344 | DB04842 | -16.66 | -18.89 | -21.34 |
| 345 | DB00434 | -16.45 | -14.36 | -13.42 |
| 346 | DB04846 | -20.66 | -20.63 | -20.77 |
| 347 | DB00442 | -19.72 | -24.15 | -21.40 |
| 348 | DB00443 | -16.86 | -18.42 | -15.55 |
| 349 | DB00446 | -20.31 | -22.98 | -16.89 |
| 350 | DB04844 | -19.33 | -18.07 | -16.18 |
| 351 | DB00448 | -19.05 | -24.10 | -13.90 |
| 352 | DB12615 | -22.82 | -24.00 | -18.67 |
| 353 | DB00444 | -19.41 | -14.37 | -14.12 |
| 354 | DB00451 | -13.06 | -15.47 | -13.71 |
| 355 | DB04854 | -18.09 | -19.25 | -16.94 |
| 356 | DB00450 | -25.85 | -27.13 | -21.68 |
| 357 | DB00452 | -25.50 | -33.76 | -20.12 |
| 358 | DB00454 | -15.47 | -11.76 | -9.04 |
| 359 | DB00445 | -24.54 | -26.04 | -26.47 |
| 360 | DB04855 | -17.74 | -18.72 | -14.22 |
| 361 | DB00456 | -21.99 | -24.05 | -20.22 |
| 362 | DB00457 | -26.67 | -26.15 | -27.74 |
| 363 | DB00458 | -11.87 | -10.71 | -11.24 |
| 364 | DB00459 | -17.36 | -16.98 | -15.16 |
| 365 | DB04861 | -23.30 | -26.24 | -22.64 |
| 366 | DB00455 | -15.66 | -18.28 | -14.25 |
| 367 | DB00461 | -12.82 | -15.14 | -14.01 |
| 368 | DB04864 | -14.87 | -24.27 | -12.70 |
| 369 | DB00462 | -13.76 | -19.08 | -10.38 |
| 370 | DB04866 | -23.28 | -22.60 | -19.91 |
| 371 | DB00464 | -7.84 | -8.47 | -6.06 |
| 372 | DB04868 | -34.91 | -36.88 | -27.87 |
| 373 | DB00465 | -22.08 | -25.10 | -22.10 |
| 374 | DB04865 | -16.80 | -18.72 | -14.00 |
| 375 | DB00467 | -24.52 | -26.11 | -23.73 |
| 376 | DB04871 | -15.47 | -11.82 | -12.18 |
| 377 | DB00468 | -19.15 | -20.46 | -11.24 |
| 378 | DB00453 | -18.97 | -22.28 | -16.49 |
| 379 | DB00469 | -22.68 | -26.46 | -23.58 |
| 380 | DB00471 | -20.83 | -17.42 | -16.02 |
| 381 | DB00472 | -13.15 | -10.12 | -10.09 |
| 382 | DB04876 | -20.08 | -22.82 | -17.08 |
| 383 | DB00473 | -15.34 | -14.33 | -12.45 |
| 384 | DB00474 | -8.83 | -13.37 | -9.07 |
| 385 | DB04878 | -15.75 | -30.19 | -14.19 |
| 386 | DB00475 | -19.12 | -17.72 | -17.61 |
| 387 | DB04880 | -22.05 | -23.92 | -19.98 |
| 388 | DB00476 | -15.08 | -15.80 | -14.16 |
| 389 | DB00470 | -11.29 | -11.50 | -7.94 |
| 390 | DB00477 | -15.23 | -9.21 | -11.19 |
| 391 | DB00478 | -8.26 | -9.51 | -6.38 |
| 392 | DB00480 | -21.60 | -19.51 | -21.23 |
| 393 | DB00482 | -19.21 | -24.37 | -15.16 |
| 394 | DB00481 | -27.11 | -29.63 | -26.24 |
| 395 | DB00484 | -23.59 | -19.01 | -20.96 |
| 396 | DB00479 | -15.84 | -20.23 | -13.80 |
| 397 | DB00485 | -24.41 | -24.67 | -20.27 |
| 398 | DB04890 | -17.39 | -17.90 | -16.36 |
| 399 | DB12667 | -16.54 | -20.08 | -16.91 |
| 400 | DB00483 | 7.96 | 7.77 | 7.71 |
| 401 | DB00488 | -10.72 | -9.30 | -7.37 |
| 402 | DB00489 | -21.35 | -26.84 | -22.51 |
| 403 | DB00487 | -24.75 | -27.65 | -22.82 |
| 404 | DB00486 | -13.91 | -13.45 | -9.39 |
| 405 | DB04896 | -12.15 | -11.12 | -8.21 |
| 406 | DB00491 | -16.70 | -24.12 | -16.18 |
| 407 | DB00490 | -17.51 | -19.66 | -16.02 |
| 408 | DB00494 | -29.32 | -25.82 | -24.72 |
| 409 | DB04908 | -24.82 | -22.85 | -19.63 |
| 410 | DB00493 | -25.01 | -27.40 | -21.18 |
| 411 | DB04845 | -13.40 | -16.33 | -10.74 |
| 412 | DB00492 | -12.43 | -6.11 | -5.48 |
| 413 | DB00495 | -23.81 | -28.57 | -22.46 |
| 414 | DB04910 | -19.25 | -21.86 | -17.42 |
| 415 | DB00498 | -15.97 | -16.82 | -14.62 |
| 416 | DB04898 | -25.60 | -24.73 | -16.28 |
| 417 | DB00499 | -19.81 | -19.50 | -15.12 |
| 418 | DB00500 | -21.68 | -21.07 | -18.89 |
| 419 | DB00501 | -20.06 | -27.03 | -22.28 |
| 420 | DB04918 | -32.49 | -33.41 | -31.47 |
| 421 | DB00496 | -22.12 | -16.92 | -17.80 |
| 422 | DB04920 | -14.76 | -13.08 | -9.56 |
| 423 | DB00497 | -21.70 | -17.17 | -15.27 |
| 424 | DB00502 | -20.85 | -23.29 | -15.20 |
| 425 | DB04930 | -11.31 | -13.96 | -10.62 |
| 426 | DB00507 | -22.07 | -28.73 | -25.16 |
| 427 | DB00508 | -12.21 | -10.72 | -11.68 |
| 428 | DB00509 | -14.22 | -18.69 | -14.31 |
| 429 | DB00504 | -12.68 | -13.53 | -10.70 |
| 430 | DB00503 | -16.35 | -8.64 | -6.78 |
| 431 | DB04938 | -13.70 | -13.81 | -12.95 |
| 432 | DB00513 | -14.69 | -14.19 | -15.94 |
| 433 | DB04942 | -24.24 | -26.51 | -20.75 |
| 434 | DB04946 | -19.62 | -23.61 | -14.75 |
| 435 | DB00514 | -15.00 | -13.45 | -12.55 |
| 436 | DB00518 | -17.76 | -20.79 | -18.46 |
| 437 | DB04948 | -13.57 | -12.70 | -15.15 |
| 438 | DB04951 | -14.03 | -15.33 | -12.12 |
| 439 | DB00519 | -17.06 | -17.66 | -18.35 |
| 440 | DB00517 | -3.84 | -9.24 | -3.25 |
| 441 | DB04953 | -22.30 | -24.81 | -23.64 |
| 442 | DB00521 | -21.01 | -23.93 | -18.86 |
| 443 | DB00523 | -15.71 | -18.89 | -16.39 |
| 444 | DB00525 | -19.22 | -16.08 | -17.71 |
| 445 | DB00524 | -17.94 | -18.93 | -16.81 |
| 446 | DB12710 | -20.49 | -19.05 | -15.75 |
| 447 | DB00527 | -19.04 | -20.55 | -14.91 |
| 448 | DB04967 | -18.75 | -18.30 | -16.30 |
| 449 | DB00529 | -16.70 | -16.50 | -13.18 |
| 450 | DB00528 | -19.94 | -20.48 | -19.10 |
| 451 | DB00531 | -12.51 | -12.03 | -10.96 |
| 452 | DB00533 | -20.10 | -18.40 | -18.88 |
| 453 | DB00530 | -16.90 | -23.55 | -16.60 |
| 454 | DB00536 | -12.81 | -17.21 | -12.37 |
| 455 | DB00535 | -35.01 | -29.26 | -27.36 |
| 456 | DB00537 | -22.95 | -27.65 | -23.64 |
| 457 | DB05015 | -23.62 | -24.87 | -22.54 |
| 458 | DB00538 | -18.61 | -23.11 | -16.80 |
| 459 | DB05016 | -29.04 | -28.48 | -28.45 |
| 460 | DB00540 | -16.32 | -14.93 | -13.54 |
| 461 | DB05013 | -17.83 | -17.35 | -19.22 |
| 462 | DB12728 | -7.02 | -6.53 | -6.27 |
| 463 | DB00539 | -14.45 | -14.63 | -12.34 |
| 464 | DB05018 | -19.28 | -25.41 | -15.56 |
| 465 | DB00542 | -15.10 | -19.38 | -13.66 |
| 466 | DB00543 | -21.17 | -21.61 | -22.65 |
| 467 | DB00544 | -15.48 | -17.21 | -14.33 |
| 468 | DB00545 | -10.53 | -12.32 | -12.26 |
| 469 | DB05039 | -22.36 | -23.45 | -19.50 |
| 470 | DB00546 | -19.22 | -17.28 | -17.27 |
| 471 | DB00548 | -14.04 | -11.48 | -15.89 |
| 472 | DB00547 | -15.43 | -20.73 | -14.18 |
| 473 | DB00550 | -15.92 | -13.45 | -12.46 |
| 474 | DB00549 | -21.85 | -25.80 | -19.28 |
| 475 | DB00551 | -13.01 | -11.69 | -9.42 |
| 476 | DB00552 | -19.73 | -21.27 | -21.28 |
| 477 | DB00553 | -13.19 | -15.64 | -12.72 |
| 478 | DB00554 | -22.23 | -24.52 | -24.41 |
| 479 | DB00555 | -18.37 | -25.93 | -15.02 |
| 480 | DB00558 | -27.09 | -31.83 | -37.30 |
| 481 | DB00557 | -18.95 | -22.60 | -15.15 |
| 482 | DB00559 | -16.13 | -21.10 | -9.63 |
| 483 | DB00561 | -17.08 | -15.51 | -14.57 |
| 484 | DB00562 | -21.84 | -22.15 | -19.21 |
| 485 | DB00563 | -26.13 | -33.16 | -26.40 |
| 486 | DB00564 | -18.36 | -16.28 | -15.57 |
| 487 | DB00566 | -11.05 | -13.31 | -13.34 |
| 488 | DB00567 | -25.91 | -26.50 | -28.33 |
| 489 | DB00568 | -20.64 | -16.50 | -15.88 |
| 490 | DB00571 | -15.19 | -21.10 | -13.19 |
| 491 | DB05154 | -21.26 | -21.33 | -22.24 |
| 492 | DB12767 | -15.89 | -17.11 | -16.83 |
| 493 | DB00573 | -16.01 | -15.42 | -17.14 |
| 494 | DB00574 | -14.28 | -10.00 | -8.57 |
| 495 | DB00575 | -16.90 | -13.73 | -16.10 |
| 496 | DB00572 | -15.96 | -16.98 | -12.97 |
| 497 | DB00576 | -18.93 | -20.48 | -21.96 |
| 498 | DB00577 | -24.01 | -24.01 | -19.75 |
| 499 | DB00578 | -25.18 | -18.52 | -19.51 |
| 500 | DB05219 | -20.45 | -18.60 | -14.35 |
| 501 | DB00580 | -19.58 | -21.61 | -16.72 |
| 502 | DB00579 | -18.61 | -15.32 | -13.79 |
| 503 | DB00582 | -15.41 | -19.19 | -11.77 |
| 504 | DB00583 | -10.44 | -11.16 | -11.17 |
| 505 | DB12783 | -30.41 | -30.46 | -28.09 |
| 506 | DB00581 | -19.50 | -21.02 | -17.43 |
| 507 | DB00585 | -24.42 | -26.06 | -23.60 |
| 508 | DB00586 | -15.55 | -18.79 | -19.03 |
| 509 | DB05239 | -30.44 | -32.02 | -26.18 |
| 510 | DB00584 | -18.01 | -21.24 | -14.82 |
| 511 | DB05245 | -20.40 | -18.50 | -17.17 |
| 512 | DB00587 | -21.78 | -21.80 | -16.69 |
| 513 | DB05246 | -11.72 | -12.69 | -11.55 |
| 514 | DB12792 | -22.03 | -23.13 | -19.31 |
| 515 | DB00588 | -12.80 | -12.01 | -8.76 |
| 516 | DB00589 | -18.37 | -18.42 | -11.77 |
| 517 | DB05260 | -11.56 | -8.21 | -11.50 |
| 518 | DB00592 | -15.10 | -12.18 | -10.80 |
| 519 | DB00593 | -12.08 | -13.49 | -10.51 |
| 520 | DB05265 | -16.29 | -15.71 | -10.63 |
| 521 | DB00594 | -23.57 | -29.58 | -24.62 |
| 522 | DB05266 | -14.97 | -13.62 | -11.52 |
| 523 | DB00590 | -25.98 | -26.35 | -24.10 |
| 524 | DB05271 | -9.87 | -11.70 | -8.99 |
| 525 | DB00591 | -18.64 | -19.56 | -14.26 |
| 526 | DB05273 | -12.48 | -14.56 | -17.75 |
| 527 | DB00596 | -15.65 | -15.88 | -14.41 |
| 528 | DB00598 | -23.69 | -23.27 | -23.40 |
| 529 | DB00599 | -10.70 | -13.25 | -9.08 |
| 530 | DB00600 | -12.95 | -13.69 | -13.42 |
| 531 | DB00601 | -20.00 | -21.36 | -19.00 |
| 532 | DB05294 | -23.72 | -29.92 | -23.21 |
| 533 | DB00560 | -27.08 | -29.66 | -21.68 |
| 534 | DB00603 | -13.67 | -13.97 | -9.18 |
| 535 | DB00604 | -23.10 | -17.47 | -15.87 |
| 536 | DB00605 | -18.36 | -18.64 | -16.13 |
| 537 | DB05316 | -23.83 | -19.16 | -19.27 |
| 538 | DB00606 | -20.38 | -21.41 | -22.54 |
| 539 | DB00607 | -23.46 | -21.46 | -16.49 |
| 540 | DB00608 | -17.69 | -19.81 | -13.12 |
| 541 | DB00609 | -14.53 | -13.91 | -13.20 |
| 542 | DB00610 | -17.64 | -19.81 | -15.64 |
| 543 | DB05351 | -19.05 | -24.10 | -13.90 |
| 544 | DB00611 | -15.89 | -14.00 | -14.54 |
| 545 | DB00612 | -15.74 | -18.27 | -13.25 |
| 546 | DB00613 | -24.63 | -20.55 | -17.67 |
| 547 | DB05381 | -14.48 | -16.45 | -11.79 |
| 548 | DB00614 | -21.04 | -19.27 | -19.51 |
| 549 | DB00617 | -10.66 | -10.78 | -10.44 |
| 550 | DB00595 | -25.60 | -23.26 | -20.41 |
| 551 | DB12834 | -15.57 | -14.47 | -14.62 |
| 552 | DB00619 | -37.93 | -31.65 | -24.45 |
| 553 | DB00621 | -12.22 | -13.61 | -13.62 |
| 554 | DB00620 | -15.87 | -18.29 | -13.97 |
| 555 | DB00622 | -24.08 | -25.20 | -23.67 |
| 556 | DB12839 | -11.35 | -14.85 | -14.54 |
| 557 | DB00624 | -18.00 | -14.77 | -10.65 |
| 558 | DB00623 | -22.31 | -24.43 | -17.56 |
| 559 | DB00625 | -11.01 | -13.48 | -9.47 |
| 560 | DB00627 | -14.87 | -18.72 | -12.28 |
| 561 | DB00628 | -18.36 | -23.24 | -17.11 |
| 562 | DB00629 | -16.08 | -19.85 | -16.26 |
| 563 | DB00630 | -18.02 | -14.91 | -13.22 |
| 564 | DB00631 | -18.31 | -20.52 | -18.38 |
| 565 | DB00618 | -25.07 | -25.71 | -25.33 |
| 566 | DB00633 | -11.94 | -10.95 | -9.59 |
| 567 | DB00632 | 5.06 | -3.86 | 5.21 |
| 568 | DB00634 | -19.05 | -20.74 | -17.09 |
| 569 | DB00635 | -15.13 | -22.14 | -17.25 |
| 570 | DB00636 | -7.43 | -11.77 | -6.83 |
| 571 | DB00637 | -21.67 | -22.19 | -19.77 |
| 572 | DB00639 | -9.24 | -13.41 | -8.10 |
| 573 | DB00640 | -19.51 | -24.64 | -20.66 |
| 574 | DB00641 | -14.49 | -17.59 | -13.67 |
| 575 | DB00642 | -29.85 | -27.79 | -22.33 |
| 576 | DB00643 | -25.60 | -25.93 | -24.73 |
| 577 | DB12867 | -26.14 | -25.03 | -17.73 |
| 578 | DB00645 | -17.14 | -17.09 | -15.29 |
| 579 | DB00597 | -22.77 | -21.96 | -23.25 |
| 580 | DB00647 | -10.44 | -8.37 | -5.96 |
| 581 | DB00648 | -6.49 | -6.48 | -5.35 |
| 582 | DB00649 | -18.60 | -25.24 | -18.23 |
| 583 | DB00650 | -33.75 | -29.88 | -27.74 |
| 584 | DB00651 | -14.97 | -18.57 | -13.56 |
| 585 | DB00652 | -13.66 | -10.60 | -10.32 |
| 586 | DB00653 | -9.91 | -10.66 | -9.39 |
| 587 | DB00654 | -14.82 | -16.73 | -14.13 |
| 588 | DB00656 | -17.42 | -19.05 | -15.47 |
| 589 | DB00655 | -16.17 | -18.10 | -13.48 |
| 590 | DB00658 | -9.22 | -9.45 | -7.62 |
| 591 | DB00657 | -11.91 | -9.81 | -7.53 |
| 592 | DB00659 | -14.67 | -16.20 | -14.83 |
| 593 | DB00660 | -15.94 | -15.19 | -13.52 |
| 594 | DB00661 | -15.28 | -12.65 | -9.87 |
| 595 | DB00662 | -21.42 | -15.53 | -12.74 |
| 596 | DB00664 | -18.71 | -22.97 | -18.44 |
| 597 | DB05521 | -29.02 | -27.38 | -21.09 |
| 598 | DB00665 | -18.98 | -20.33 | -20.45 |
| 599 | DB00663 | -16.27 | -20.05 | -15.43 |
| 600 | DB00668 | -23.83 | -19.58 | -20.20 |
| 601 | DB05541 | -11.78 | -14.93 | -13.58 |
| 602 | DB00669 | -18.44 | -18.98 | -17.76 |
| 603 | DB00670 | -31.77 | -27.30 | -24.63 |
| 604 | DB00671 | -32.32 | -29.84 | -26.32 |
| 605 | DB00672 | -13.70 | -21.27 | -18.32 |
| 606 | DB00673 | -19.21 | -23.23 | -18.65 |
| 607 | DB00675 | -13.38 | -15.03 | -16.70 |
| 608 | DB00676 | -11.51 | -16.27 | -12.25 |
| 609 | DB00677 | -7.42 | -7.05 | -6.40 |
| 610 | DB00678 | -19.31 | -19.17 | -18.26 |
| 611 | DB00679 | -16.18 | -16.56 | -13.62 |
| 612 | DB00674 | -16.13 | -18.37 | -19.93 |
| 613 | DB00680 | -24.99 | -23.80 | -20.88 |
| 614 | DB00682 | -15.07 | -21.90 | -16.71 |
| 615 | DB00683 | -15.52 | -14.73 | -14.97 |
| 616 | DB12911 | -12.33 | -10.42 | -6.66 |
| 617 | DB00685 | -26.76 | -27.86 | -21.78 |
| 618 | DB00684 | -18.95 | -26.50 | -15.14 |
| 619 | DB00686 | -22.04 | -22.17 | -12.90 |
| 620 | DB05630 | -19.28 | -18.45 | -15.82 |
| 621 | DB00687 | -18.09 | -22.40 | -14.32 |
| 622 | DB00689 | -30.70 | -26.85 | -31.78 |
| 623 | DB00688 | -22.11 | -21.42 | -15.89 |
| 624 | DB12924 | -20.93 | -25.63 | -20.57 |
| 625 | DB00690 | -16.52 | -15.31 | -9.93 |
| 626 | DB00692 | -18.84 | -20.33 | -19.64 |
| 627 | DB00693 | -18.96 | -21.41 | -16.96 |
| 628 | DB00691 | -19.79 | -18.99 | -15.97 |
| 629 | DB00695 | -23.39 | -25.87 | -22.18 |
| 630 | DB00696 | -25.54 | -22.13 | -15.45 |
| 631 | DB00697 | -16.46 | -16.17 | -15.26 |
| 632 | DB00698 | -21.69 | -24.07 | -21.06 |
| 633 | DB05676 | -18.29 | -16.19 | -13.57 |
| 634 | DB00694 | -28.21 | -25.42 | -19.15 |
| 635 | DB00699 | -21.67 | -14.56 | -13.69 |
| 636 | DB00701 | -20.44 | -19.92 | -13.60 |
| 637 | DB12938 | -17.17 | -17.92 | -12.79 |
| 638 | DB00703 | -23.68 | -21.37 | -16.69 |
| 639 | DB00704 | -22.19 | -15.70 | -14.33 |
| 640 | DB00700 | -20.03 | -17.87 | -15.82 |
| 641 | DB00705 | -31.32 | -34.25 | -26.83 |
| 642 | DB00706 | -17.94 | -23.11 | -14.30 |
| 643 | DB00709 | -17.18 | -21.85 | -20.96 |
| 644 | DB00710 | -12.75 | -13.32 | -10.41 |
| 645 | DB12945 | -20.44 | -19.82 | -17.79 |
| 646 | DB00708 | -15.04 | -13.34 | -10.02 |
| 647 | DB00712 | -16.71 | -19.24 | -19.01 |
| 648 | DB00711 | -15.00 | -14.42 | -11.87 |
| 649 | DB00713 | -27.99 | -22.48 | -19.98 |
| 650 | DB00715 | -19.75 | -21.34 | -19.77 |
| 651 | DB00716 | -20.42 | -21.56 | -16.48 |
| 652 | DB12952 | -15.77 | -18.80 | -17.24 |
| 653 | DB00714 | -21.09 | -18.17 | -18.55 |
| 654 | DB12954 | -16.37 | -29.62 | -18.14 |
| 655 | DB00717 | -14.71 | -14.38 | -9.67 |
| 656 | DB00718 | -11.88 | -13.28 | -10.69 |
| 657 | DB00720 | -11.53 | -13.83 | -6.75 |
| 658 | DB00721 | -17.34 | -13.71 | -13.13 |
| 659 | DB00722 | -25.08 | -25.06 | -23.80 |
| 660 | DB00723 | -12.64 | -21.01 | -11.60 |
| 661 | DB00724 | -16.31 | -16.10 | -11.87 |
| 662 | DB00725 | -13.51 | -18.68 | -12.27 |
| 663 | DB00726 | -9.82 | -8.34 | -8.84 |
| 664 | DB00727 | -17.51 | -14.35 | -13.79 |
| 665 | DB00729 | -13.51 | -12.17 | -11.44 |
| 666 | DB05812 | -15.17 | -18.31 | -18.02 |
| 667 | DB00730 | -15.53 | -24.80 | -15.59 |
| 668 | DB00731 | -16.82 | -17.74 | -17.28 |
| 669 | DB00719 | -18.49 | -18.24 | -16.37 |
| 670 | DB00733 | -14.02 | -13.49 | -11.63 |
| 671 | DB00734 | -16.34 | -16.26 | -15.28 |
| 672 | DB00735 | -15.25 | -12.32 | -14.27 |
| 673 | DB00736 | -15.86 | -21.65 | -13.73 |
| 674 | DB00737 | -18.80 | -18.45 | -15.58 |
| 675 | DB00738 | -25.13 | -26.65 | -21.60 |
| 676 | DB00739 | -23.12 | -17.87 | -17.91 |
| 677 | DB00740 | -14.93 | -14.80 | -13.30 |
| 678 | DB00742 | -17.41 | -19.99 | -10.16 |
| 679 | DB00741 | -14.11 | -22.08 | -15.08 |
| 680 | DB00744 | -17.37 | -21.40 | -14.62 |
| 681 | DB00745 | -16.72 | -21.92 | -15.15 |
| 682 | DB00743 | -19.87 | -16.10 | -18.44 |
| 683 | DB00746 | -31.31 | -34.62 | -22.80 |
| 684 | DB00747 | -19.12 | -18.01 | -19.05 |
| 685 | DB00748 | -13.41 | -14.68 | -12.18 |
| 686 | DB00750 | -16.89 | -18.72 | -16.63 |
| 687 | DB00749 | -15.28 | -21.41 | -13.52 |
| 688 | DB00752 | -11.39 | -13.96 | -10.11 |
| 689 | DB00751 | -15.43 | -19.97 | -18.84 |
| 690 | DB00754 | -14.03 | -16.22 | -11.67 |
| 691 | DB00756 | -11.78 | -10.43 | -10.83 |
| 692 | DB00755 | -16.43 | -19.25 | -18.80 |
| 693 | DB00757 | -26.22 | -27.63 | -20.38 |
| 694 | DB00758 | -11.66 | -13.96 | -9.75 |
| 695 | DB00760 | -31.22 | -25.14 | -22.95 |
| 696 | DB00762 | -17.27 | -19.79 | -22.14 |
| 697 | DB05990 | -15.44 | -16.75 | -15.36 |
| 698 | DB00763 | -7.54 | -9.22 | -9.14 |
| 699 | DB00764 | -13.68 | -16.13 | -15.52 |
| 700 | DB00765 | -18.98 | -19.14 | -15.69 |
| 701 | DB00766 | -14.91 | -16.36 | -15.97 |
| 702 | DB00768 | -21.89 | -19.77 | -17.36 |
| 703 | DB06016 | -21.06 | -19.24 | -16.03 |
| 704 | DB00770 | -16.69 | -14.95 | -13.05 |
| 705 | DB00771 | -16.90 | -11.09 | -13.04 |
| 706 | DB00772 | -6.74 | -8.27 | -2.40 |
| 707 | DB00769 | -16.52 | -21.32 | -14.10 |
| 708 | DB00773 | -19.21 | -25.16 | -17.16 |
| 709 | DB00774 | -17.38 | -21.17 | -20.55 |
| 710 | DB00775 | -25.77 | -20.03 | -16.61 |
| 711 | DB00776 | -20.64 | -21.47 | -18.80 |
| 712 | DB00777 | -17.91 | -15.91 | -13.22 |
| 713 | DB00779 | -16.41 | -18.08 | -17.53 |
| 714 | DB00780 | -16.71 | -19.07 | -14.57 |
| 715 | DB00759 | -22.18 | -25.90 | -23.26 |
| 716 | DB00782 | -11.51 | -10.87 | -11.68 |
| 717 | DB13025 | -20.75 | -21.84 | -15.85 |
| 718 | DB00783 | -13.85 | -20.18 | -13.76 |
| 719 | DB00784 | -20.99 | -24.38 | -22.64 |
| 720 | DB00786 | -23.61 | -19.38 | -14.36 |
| 721 | DB00787 | -17.08 | -20.30 | -17.42 |
| 722 | DB00788 | -17.53 | -17.73 | -13.65 |
| 723 | DB00789 | -14.93 | -18.09 | -15.49 |
| 724 | DB06144 | -19.95 | -24.19 | -20.65 |
| 725 | DB00790 | -16.62 | -22.08 | -16.36 |
| 726 | DB00791 | -12.82 | -15.38 | -6.88 |
| 727 | DB06147 | -16.96 | -17.43 | -16.48 |
| 728 | DB00792 | -13.72 | -11.18 | -11.01 |
| 729 | DB06148 | -17.24 | -13.94 | -15.11 |
| 730 | DB00793 | -4.57 | -6.54 | -3.80 |
| 731 | DB06150 | -22.72 | -21.86 | -23.37 |
| 732 | DB00794 | -12.94 | -18.24 | -11.65 |
| 733 | DB06151 | -11.89 | -14.93 | -14.45 |
| 734 | DB00795 | -30.45 | -32.17 | -26.91 |
| 735 | DB06152 | -16.95 | -15.56 | -15.27 |
| 736 | DB00796 | -24.23 | -24.29 | -22.85 |
| 737 | DB06153 | -14.71 | -15.51 | -13.57 |
| 738 | DB00797 | -13.57 | -12.16 | -15.05 |
| 739 | DB06154 | -16.47 | -12.73 | -11.68 |
| 740 | DB00798 | -23.32 | -20.89 | -19.52 |
| 741 | DB06155 | -19.65 | -19.48 | -20.56 |
| 742 | DB00799 | -11.36 | -14.74 | -9.69 |
| 743 | DB00800 | -17.66 | -19.92 | -16.95 |
| 744 | DB00801 | -14.67 | -13.24 | -13.53 |
| 745 | DB00802 | -15.48 | -11.19 | -11.55 |
| 746 | DB00804 | -11.96 | -8.00 | -7.05 |
| 747 | DB00805 | -22.47 | -24.85 | -21.61 |
| 748 | DB00806 | -11.98 | -14.84 | -9.04 |
| 749 | DB00807 | -13.81 | -14.93 | -9.94 |
| 750 | DB06176 | -21.83 | -13.83 | -15.90 |
| 751 | DB00808 | -30.03 | -24.08 | -20.44 |
| 752 | DB00809 | -17.01 | -15.87 | -13.86 |
| 753 | DB00810 | -13.70 | -15.99 | -10.28 |
| 754 | DB00811 | -21.80 | -24.00 | -24.46 |
| 755 | DB00812 | -17.42 | -15.18 | -14.21 |
| 756 | DB00813 | -20.68 | -24.59 | -15.12 |
| 757 | DB00814 | -22.29 | -24.81 | -18.45 |
| 758 | DB00815 | -7.00 | -7.49 | -5.97 |
| 759 | DB00816 | -20.31 | -20.65 | -20.28 |
| 760 | DB00817 | -18.57 | -19.41 | -18.64 |
| 761 | DB06193 | -28.99 | -30.05 | -28.31 |
| 762 | DB13063 | -14.58 | -14.33 | -12.48 |
| 763 | DB00818 | -7.89 | -10.10 | -7.42 |
| 764 | DB00819 | -20.32 | -26.00 | -18.06 |
| 765 | DB00820 | -22.70 | -24.19 | -23.44 |
| 766 | DB00821 | -19.65 | -15.05 | -16.14 |
| 767 | DB00822 | 0.86 | 2.82 | 0.67 |
| 768 | DB00823 | -6.97 | -8.26 | -8.72 |
| 769 | DB13074 | -36.79 | -31.40 | -33.04 |
| 770 | DB00824 | -16.39 | -19.00 | -12.39 |
| 771 | DB00825 | -8.18 | -10.31 | -8.18 |
| 772 | DB06201 | -18.60 | -23.24 | -19.03 |
| 773 | DB06202 | -17.84 | -16.31 | -14.75 |
| 774 | DB00827 | -19.33 | -22.53 | -19.16 |
| 775 | DB06203 | -23.90 | -21.63 | -20.84 |
| 776 | DB00828 | -12.11 | -12.76 | -10.22 |
| 777 | DB06204 | -9.68 | -13.11 | -10.54 |
| 778 | DB00829 | -16.65 | -15.14 | -15.40 |
| 779 | DB00830 | -16.05 | -13.91 | -14.53 |
| 780 | DB06207 | -18.24 | -19.66 | -15.72 |
| 781 | DB00831 | -22.21 | -14.96 | -16.07 |
| 782 | DB06209 | -11.26 | -14.18 | -11.09 |
| 783 | DB00832 | -11.32 | -14.67 | -10.99 |
| 784 | DB06210 | -23.73 | -26.34 | -21.95 |
| 785 | DB00833 | -26.73 | -27.23 | -27.60 |
| 786 | DB06211 | -32.66 | -37.13 | -28.85 |
| 787 | DB00834 | -13.14 | -12.23 | -10.86 |
| 788 | DB06212 | -28.12 | -24.82 | -23.86 |
| 789 | DB00835 | -12.81 | -12.69 | -9.02 |
| 790 | DB06213 | -28.17 | -27.01 | -26.20 |
| 791 | DB00836 | -13.12 | -13.04 | -10.21 |
| 792 | DB00837 | -23.00 | -18.09 | -20.56 |
| 793 | DB06216 | -14.35 | -15.03 | -14.27 |
| 794 | DB00838 | -15.83 | -21.07 | -16.21 |
| 795 | DB06217 | -22.01 | -16.98 | -15.77 |
| 796 | DB00839 | -20.00 | -25.42 | -17.26 |
| 797 | DB06218 | -17.25 | -18.25 | -13.81 |
| 798 | DB00841 | -18.29 | -20.97 | -17.61 |
| 799 | DB00842 | -17.27 | -21.35 | -16.92 |
| 800 | DB06228 | -21.98 | -27.58 | -19.38 |
| 801 | DB00843 | -19.69 | -20.99 | -17.29 |
| 802 | DB06230 | -17.02 | -14.80 | -15.20 |
| 803 | DB00845 | -21.07 | -22.01 | -21.86 |
| 804 | DB00844 | -20.79 | -16.96 | -15.31 |
| 805 | DB00847 | -9.77 | -9.24 | -9.37 |
| 806 | DB00846 | -21.70 | -19.44 | -13.78 |
| 807 | DB00848 | -9.83 | -14.16 | -11.29 |
| 808 | DB00850 | -20.63 | -25.55 | -20.45 |
| 809 | DB00851 | -21.71 | -20.70 | -20.03 |
| 810 | DB00852 | -15.31 | -13.14 | -12.13 |
| 811 | DB06243 | -13.13 | -17.27 | -16.86 |
| 812 | DB00849 | -12.61 | -20.22 | -14.32 |
| 813 | DB06237 | -25.89 | -25.21 | -23.42 |
| 814 | DB00853 | -21.33 | -21.07 | -18.99 |
| 815 | DB00855 | -21.32 | -17.53 | -14.59 |
| 816 | DB00854 | -14.25 | -13.24 | -12.72 |
| 817 | DB06249 | -22.51 | -22.99 | -17.86 |
| 818 | DB13092 | -18.91 | -25.11 | -14.95 |
| 819 | DB00856 | -11.40 | -14.47 | -9.75 |
| 820 | DB00857 | -13.64 | -9.93 | -9.32 |
| 821 | DB00859 | -17.83 | -14.99 | -13.99 |
| 822 | DB00858 | -11.40 | -17.93 | -10.36 |
| 823 | DB06255 | -15.85 | -22.90 | -12.91 |
| 824 | DB00861 | -19.70 | -24.82 | -20.67 |
| 825 | DB13114 | -15.36 | -14.25 | -12.90 |
| 826 | DB00860 | -14.96 | -19.63 | -15.12 |
| 827 | DB00863 | -23.95 | -25.24 | -23.70 |
| 828 | DB06261 | -9.79 | -15.39 | -7.66 |
| 829 | DB06262 | -19.81 | -20.82 | -21.84 |
| 830 | DB00865 | -11.86 | -8.46 | -11.40 |
| 831 | DB06263 | -24.14 | -22.84 | -22.68 |
| 832 | DB00862 | -19.64 | -22.34 | -18.07 |
| 833 | DB06264 | -16.20 | -20.64 | -12.63 |
| 834 | DB00867 | -20.27 | -21.00 | -16.89 |
| 835 | DB00866 | -15.17 | -16.71 | -13.10 |
| 836 | DB06267 | -19.02 | -22.16 | -16.26 |
| 837 | DB00869 | -18.68 | -22.37 | -17.67 |
| 838 | DB06268 | -25.11 | -26.47 | -24.94 |
| 839 | DB00870 | -18.54 | -20.47 | -17.94 |
| 840 | DB06272 | -13.74 | -16.80 | -12.13 |
| 841 | DB00871 | -18.47 | -19.40 | -15.83 |
| 842 | DB06274 | -23.40 | -22.12 | -17.98 |
| 843 | DB00868 | -5.61 | -13.22 | 1.30 |
| 844 | DB00872 | -32.79 | -31.54 | -26.48 |
| 845 | DB00874 | -12.86 | -16.60 | -11.23 |
| 846 | DB00875 | -22.05 | -26.35 | -21.14 |
| 847 | DB06282 | -16.63 | -18.77 | -20.11 |
| 848 | DB00873 | -9.40 | -14.70 | -9.54 |
| 849 | DB00876 | -22.82 | -18.05 | -16.56 |
| 850 | DB06288 | -24.21 | -25.19 | -18.50 |
| 851 | DB13136 | -15.76 | -18.00 | -14.91 |
| 852 | DB00879 | -17.09 | -21.86 | -20.30 |
| 853 | DB06292 | -15.74 | -20.51 | -15.51 |
| 854 | DB00880 | -19.49 | -22.93 | -18.37 |
| 855 | DB13139 | -18.92 | -19.03 | -18.81 |
| 856 | DB00881 | -18.09 | -20.63 | -17.13 |
| 857 | DB00882 | -14.48 | -13.31 | -12.25 |
| 858 | DB00883 | -18.20 | -15.59 | -19.81 |
| 859 | DB06290 | -13.79 | -9.82 | -5.45 |
| 860 | DB00884 | -16.34 | -22.70 | -14.50 |
| 861 | DB00885 | -19.46 | -22.40 | -21.48 |
| 862 | DB13146 | -16.80 | -18.96 | -15.02 |
| 863 | DB00887 | -18.11 | -21.56 | -19.69 |
| 864 | DB13154 | -11.12 | -10.30 | -8.53 |
| 865 | DB00888 | -3.16 | -2.28 | -1.35 |
| 866 | DB13153 | -5.91 | -7.92 | -5.84 |
| 867 | DB13155 | -24.82 | -20.65 | -18.91 |
| 868 | DB00890 | -22.19 | -21.25 | -17.02 |
| 869 | DB13156 | -24.10 | -22.57 | -20.07 |
| 870 | DB00891 | -20.68 | -18.97 | -17.36 |
| 871 | DB06335 | -19.41 | -17.25 | -22.93 |
| 872 | DB13157 | -5.56 | -6.58 | -2.55 |
| 873 | DB00889 | -20.65 | -19.73 | -19.32 |
| 874 | DB00892 | -20.04 | -14.42 | -12.00 |
| 875 | DB00894 | -15.34 | -16.35 | -11.95 |
| 876 | DB13163 | -15.49 | -11.59 | -11.51 |
| 877 | DB00895 | -27.81 | -32.43 | -25.66 |
| 878 | DB13164 | -31.31 | -29.61 | -26.23 |
| 879 | DB00897 | -21.38 | -13.01 | -16.32 |
| 880 | DB13158 | -15.07 | -14.88 | -13.44 |
| 881 | DB00898 | -10.50 | -6.68 | -4.32 |
| 882 | DB00896 | -11.34 | -14.72 | -13.47 |
| 883 | DB13167 | -11.62 | -12.24 | -10.75 |
| 884 | DB00900 | -21.60 | -23.03 | -24.12 |
| 885 | DB13165 | -23.16 | -23.53 | -18.88 |
| 886 | DB00902 | -14.64 | -14.44 | -12.84 |
| 887 | DB00903 | -17.12 | -18.63 | -14.76 |
| 888 | DB00899 | -17.46 | -20.73 | -10.77 |
| 889 | DB00904 | -17.18 | -15.22 | -15.06 |
| 890 | DB13178 | -13.60 | -20.52 | -13.25 |
| 891 | DB00905 | -16.80 | -16.81 | -12.65 |
| 892 | DB06401 | -18.45 | -19.13 | -15.41 |
| 893 | DB13180 | -17.19 | -21.05 | -17.15 |
| 894 | DB00907 | -15.73 | -17.90 | -11.04 |
| 895 | DB06403 | -13.22 | -17.57 | -15.59 |
| 896 | DB00906 | -19.46 | -22.06 | -19.03 |
| 897 | DB00908 | -19.71 | -16.47 | -12.91 |
| 898 | DB06410 | -5.23 | -14.50 | -6.20 |
| 899 | DB00909 | -14.43 | -21.48 | -13.98 |
| 900 | DB06412 | -17.16 | -15.63 | -13.90 |
| 901 | DB00910 | -14.37 | -17.39 | -10.83 |
| 902 | DB06413 | -17.34 | -22.24 | -16.07 |
| 903 | DB00911 | -14.83 | -15.04 | -14.67 |
| 904 | DB06414 | -24.96 | -22.27 | -20.85 |
| 905 | DB00912 | -26.83 | -24.66 | -21.50 |
| 906 | DB13209 | -11.55 | -8.18 | -8.78 |
| 907 | DB00915 | -10.54 | -10.01 | -9.34 |
| 908 | DB00916 | -14.29 | -14.89 | -13.64 |
| 909 | DB00917 | -17.43 | -18.02 | -15.03 |
| 910 | DB00918 | -15.40 | -17.58 | -16.74 |
| 911 | DB13231 | -12.81 | -12.43 | -11.46 |
| 912 | DB00913 | -16.03 | -18.20 | -16.37 |
| 913 | DB00922 | -21.56 | -22.40 | -21.00 |
| 914 | DB06441 | -17.69 | -18.73 | -17.23 |
| 915 | DB00923 | -32.56 | -32.41 | -23.79 |
| 916 | DB00924 | -14.39 | -14.27 | -11.32 |
| 917 | DB13257 | -9.91 | -10.66 | -9.39 |
| 918 | DB00925 | -11.16 | -8.69 | -11.18 |
| 919 | DB13259 | -0.14 | 1.08 | 1.03 |
| 920 | DB00927 | -23.83 | -32.56 | -24.84 |
| 921 | DB00920 | -20.49 | -18.60 | -17.84 |
| 922 | DB13268 | -23.87 | -24.16 | -21.16 |
| 923 | DB00928 | -16.30 | -21.98 | -19.29 |
| 924 | DB13269 | -9.47 | -12.49 | -6.49 |
| 925 | DB00919 | -21.97 | -26.41 | -20.73 |
| 926 | DB00929 | -18.10 | -15.34 | -7.21 |
| 927 | DB00932 | -26.86 | -22.32 | -22.52 |
| 928 | DB00933 | -20.21 | -15.04 | -13.23 |
| 929 | DB13322 | -12.53 | -10.45 | -7.56 |
| 930 | DB00935 | -15.23 | -12.47 | -13.55 |
| 931 | DB00934 | -13.62 | -13.82 | -11.64 |
| 932 | DB13337 | -19.03 | -19.00 | -16.88 |
| 933 | DB00936 | -14.86 | -16.58 | -15.54 |
| 934 | DB13345 | -19.69 | -24.76 | -11.99 |
| 935 | DB00937 | -11.56 | -16.27 | -11.38 |
| 936 | DB13346 | -14.86 | -13.90 | -12.51 |
| 937 | DB00939 | -20.76 | -22.35 | -23.93 |
| 938 | DB00938 | -20.19 | -22.17 | -18.69 |
| 939 | DB00940 | -11.96 | -9.05 | -11.70 |
| 940 | DB00941 | -8.24 | -10.67 | -7.69 |
| 941 | DB00942 | -12.57 | -12.05 | -11.57 |
| 942 | DB00943 | -15.54 | -20.48 | -18.85 |
| 943 | DB00945 | -14.48 | -16.34 | -13.55 |
| 944 | DB06480 | -26.45 | -22.00 | -19.47 |
| 945 | DB13421 | -18.51 | -24.89 | -19.66 |
| 946 | DB00944 | -8.69 | -8.97 | 2.22 |
| 947 | DB00946 | -14.40 | -15.51 | -15.14 |
| 948 | DB13444 | -19.18 | -23.51 | -19.98 |
| 949 | DB00947 | -6.78 | -10.11 | -6.60 |
| 950 | DB00949 | -18.99 | -22.85 | -13.13 |
| 951 | DB00950 | -22.18 | -19.52 | -14.31 |
| 952 | DB00951 | -18.32 | -18.05 | -18.51 |
| 953 | DB00948 | -34.30 | -25.62 | -24.81 |
| 954 | DB00953 | -14.79 | -14.56 | -15.66 |
| 955 | DB00952 | -20.30 | -22.99 | -18.25 |
| 956 | DB00955 | -25.13 | -21.78 | -22.36 |
| 957 | DB00956 | -20.29 | -16.43 | -11.92 |
| 958 | DB00957 | -12.39 | -12.20 | -10.49 |
| 959 | DB00959 | -14.34 | -17.12 | -12.28 |
| 960 | DB00960 | -19.28 | -18.77 | -18.51 |
| 961 | DB13583 | -12.93 | -16.33 | -12.84 |
| 962 | DB00962 | -22.22 | -24.03 | -19.04 |
| 963 | DB00963 | -22.12 | -23.80 | -20.88 |
| 964 | DB13595 | -4.49 | -4.21 | -4.21 |
| 965 | DB00964 | -18.58 | -17.99 | -15.93 |
| 966 | DB00961 | -20.82 | -16.61 | -17.35 |
| 967 | DB00966 | -21.31 | -23.91 | -19.26 |
| 968 | DB00968 | -18.20 | -21.51 | -20.38 |
| 969 | DB13620 | -14.57 | -19.29 | -16.88 |
| 970 | DB00969 | -21.43 | -19.41 | -16.45 |
| 971 | DB00967 | -18.49 | -18.99 | -17.21 |
| 972 | DB00972 | -20.08 | -17.81 | -16.46 |
| 973 | DB00973 | -23.96 | -17.86 | -15.11 |
| 974 | DB00974 | -19.84 | -18.46 | -14.16 |
| 975 | DB00931 | -18.24 | -21.08 | -17.38 |
| 976 | DB00977 | -15.52 | -16.71 | -10.94 |
| 977 | DB00975 | -19.04 | -19.61 | -16.18 |
| 978 | DB13707 | -14.98 | -13.10 | -12.56 |
| 979 | DB00979 | -12.36 | -14.24 | -11.38 |
| 980 | DB13682 | -28.93 | -25.94 | -24.87 |
| 981 | DB00978 | -24.99 | -28.00 | -23.10 |
| 982 | DB00980 | -15.39 | -14.42 | -13.34 |
| 983 | DB00982 | -16.35 | -18.05 | -14.07 |
| 984 | DB13749 | -15.03 | -19.16 | -16.97 |
| 985 | DB00981 | -16.15 | -14.06 | -14.52 |
| 986 | DB06589 | -33.65 | -28.17 | -29.90 |
| 987 | DB13747 | -16.55 | -15.55 | -10.68 |
| 988 | DB00983 | -28.22 | -24.97 | -21.69 |
| 989 | DB06590 | -29.80 | -42.57 | -30.39 |
| 990 | DB00985 | -13.46 | -10.87 | -9.94 |
| 991 | DB06594 | -16.33 | -16.13 | -13.69 |
| 992 | DB00986 | -14.06 | -10.24 | -11.66 |
| 993 | DB06595 | -24.15 | -28.22 | -15.68 |
| 994 | DB00984 | -21.21 | -17.91 | -13.33 |
| 995 | DB00987 | -19.08 | -26.47 | -18.64 |
| 996 | DB13711 | -14.83 | -16.78 | -11.67 |
| 997 | DB00988 | -20.96 | -17.07 | -14.42 |
| 998 | DB00989 | -14.92 | -15.83 | -13.69 |
| 999 | DB06603 | -21.89 | -26.77 | -20.95 |
| 1000 | DB00990 | -14.74 | -14.68 | -10.64 |
| 1001 | DB06605 | -22.68 | -27.12 | -19.38 |
| 1002 | DB00992 | -14.81 | -15.78 | -11.04 |
| 1003 | DB00991 | -18.44 | -17.65 | -16.74 |
| 1004 | DB13783 | -22.91 | -19.67 | -22.65 |
| 1005 | DB00993 | -19.72 | -20.95 | -18.12 |
| 1006 | DB13800 | -12.37 | -11.10 | -11.39 |
| 1007 | DB00996 | -10.35 | -12.56 | -15.64 |
| 1008 | DB06614 | -23.21 | -21.17 | -24.32 |
| 1009 | DB00997 | -32.04 | -25.31 | -25.73 |
| 1010 | DB13805 | -13.52 | -18.12 | -10.12 |
| 1011 | DB00999 | -18.33 | -25.50 | -20.71 |
| 1012 | DB06616 | -35.06 | -26.90 | -31.78 |
| 1013 | DB00998 | -24.38 | -24.64 | -20.00 |
| 1014 | DB06623 | -21.85 | -23.23 | -21.86 |
| 1015 | DB01000 | -28.75 | -22.19 | -24.81 |
| 1016 | DB01001 | -17.73 | -18.58 | -16.87 |
| 1017 | DB01003 | -30.80 | -32.63 | -26.11 |
| 1018 | DB06626 | -24.75 | -26.78 | -22.83 |
| 1019 | DB13838 | -11.28 | -11.28 | -13.32 |
| 1020 | DB01004 | -14.91 | -22.38 | -17.06 |
| 1021 | DB01005 | -15.18 | -16.43 | -9.70 |
| 1022 | DB13842 | -15.09 | -17.65 | -12.98 |
| 1023 | DB01002 | -19.46 | -15.90 | -14.21 |
| 1024 | DB01006 | -15.94 | -17.41 | -14.24 |
| 1025 | DB01007 | -10.66 | -10.71 | -7.75 |
| 1026 | DB06637 | -12.92 | -13.35 | -13.89 |
| 1027 | DB01008 | -12.54 | -10.09 | -9.30 |
| 1028 | DB06636 | -24.47 | -20.96 | -21.34 |
| 1029 | DB13851 | -14.07 | -13.50 | -10.41 |
| 1030 | DB01010 | -10.25 | -10.49 | -7.98 |
| 1031 | DB13853 | -7.63 | -9.32 | -6.73 |
| 1032 | DB01009 | -21.46 | -22.28 | -22.98 |
| 1033 | DB13854 | -3.49 | -5.04 | -4.83 |
| 1034 | DB01011 | -19.77 | -18.65 | -13.41 |
| 1035 | DB13858 | -10.96 | -11.91 | -10.70 |
| 1036 | DB01012 | -14.46 | -15.65 | -14.16 |
| 1037 | DB01014 | -27.37 | -30.15 | -27.21 |
| 1038 | DB06654 | -22.39 | -28.22 | -18.94 |
| 1039 | DB13857 | -13.93 | -14.04 | -10.08 |
| 1040 | DB01015 | -19.47 | -19.81 | -17.49 |
| 1041 | DB01016 | -27.24 | -24.82 | -18.96 |
| 1042 | DB01013 | -11.81 | -12.26 | -9.73 |
| 1043 | DB13867 | -10.91 | -14.68 | -13.26 |
| 1044 | DB01018 | -15.87 | -17.88 | -14.18 |
| 1045 | DB01019 | -6.82 | -8.39 | -6.86 |
| 1046 | DB01020 | -15.81 | -17.75 | -16.11 |
| 1047 | DB13873 | -20.84 | -19.94 | -16.11 |
| 1048 | DB01021 | -16.65 | -22.41 | -21.34 |
| 1049 | DB13874 | -29.59 | -31.64 | -23.34 |
| 1050 | DB01022 | -12.52 | -10.91 | -7.99 |
| 1051 | DB13872 | -15.60 | -16.32 | -16.17 |
| 1052 | DB01023 | -13.54 | -13.07 | -12.63 |
| 1053 | DB01024 | -23.95 | -21.57 | -20.52 |
| 1054 | DB01025 | -25.06 | -23.80 | -20.87 |
| 1055 | DB01026 | -18.27 | -18.45 | -15.92 |
| 1056 | DB01028 | -3.89 | -4.52 | -1.26 |
| 1057 | DB13882 | -12.89 | -13.28 | -9.10 |
| 1058 | DB01029 | -19.52 | -22.26 | -18.39 |
| 1059 | DB13908 | -6.39 | -6.88 | -6.67 |
| 1060 | DB01031 | -9.67 | -13.42 | -9.36 |
| 1061 | DB01032 | -14.88 | -17.06 | -14.05 |
| 1062 | DB13910 | -6.93 | -8.79 | -7.71 |
| 1063 | DB01033 | -16.75 | -14.00 | -13.79 |
| 1064 | DB06684 | -31.62 | -33.15 | -23.90 |
| 1065 | DB13911 | -18.79 | -15.33 | -13.49 |
| 1066 | DB01034 | -15.03 | -19.65 | -14.08 |
| 1067 | DB01035 | -21.75 | -19.12 | -15.35 |
| 1068 | DB06689 | -1.73 | -5.47 | -3.09 |
| 1069 | DB01036 | -11.16 | -8.79 | -8.11 |
| 1070 | DB06691 | -13.61 | -13.55 | -9.22 |
| 1071 | DB01037 | -8.51 | -8.97 | -9.43 |
| 1072 | DB06694 | -11.38 | -8.79 | -12.50 |
| 1073 | DB01030 | -16.12 | -24.71 | -15.63 |
| 1074 | DB01039 | -16.15 | -16.49 | -14.28 |
| 1075 | DB06696 | -19.92 | -19.98 | -17.91 |
| 1076 | DB01040 | -25.94 | -26.18 | -25.76 |
| 1077 | DB06695 | -28.78 | -39.45 | -21.28 |
| 1078 | DB01041 | -19.74 | -22.49 | -21.27 |
| 1079 | DB06698 | -12.21 | -12.94 | -9.48 |
| 1080 | DB06697 | -16.62 | -14.46 | -10.58 |
| 1081 | DB01043 | -11.19 | -10.46 | -11.81 |
| 1082 | DB06700 | -14.93 | -11.39 | -10.59 |
| 1083 | DB01042 | -9.34 | -13.89 | -9.07 |
| 1084 | DB06701 | -15.05 | -14.89 | -13.66 |
| 1085 | DB01044 | -23.52 | -22.51 | -23.69 |
| 1086 | DB06702 | -7.25 | -9.34 | -5.06 |
| 1087 | DB01046 | -12.38 | -13.68 | -9.30 |
| 1088 | DB06704 | -17.54 | -21.55 | -13.81 |
| 1089 | DB13931 | -27.26 | -29.25 | -23.78 |
| 1090 | DB01048 | -21.96 | -21.42 | -16.45 |
| 1091 | DB06705 | -16.74 | -10.79 | -9.41 |
| 1092 | DB01050 | -12.05 | -14.08 | -12.36 |
| 1093 | DB06706 | -10.97 | -6.61 | -7.40 |
| 1094 | DB01047 | -13.83 | -21.08 | -11.86 |
| 1095 | DB06707 | -17.01 | -18.07 | -19.29 |
| 1096 | DB01053 | -23.59 | -19.94 | -17.48 |
| 1097 | DB06708 | -14.32 | -11.18 | -6.07 |
| 1098 | DB01017 | -20.86 | -25.92 | -24.49 |
| 1099 | DB06709 | -4.08 | -5.44 | -2.64 |
| 1100 | DB01055 | -19.71 | -19.30 | -18.61 |
| 1101 | DB06710 | -13.49 | -14.36 | -9.65 |
| 1102 | DB01054 | -21.31 | -20.40 | -20.61 |
| 1103 | DB06711 | -15.77 | -14.78 | -16.92 |
| 1104 | DB01051 | -19.61 | -23.30 | -20.56 |
| 1105 | DB13941 | -24.33 | -27.53 | -20.17 |
| 1106 | DB01056 | -18.61 | -20.44 | -14.91 |
| 1107 | DB06712 | -23.32 | -20.36 | -19.26 |
| 1108 | DB13943 | -19.38 | -15.98 | -11.94 |
| 1109 | DB01057 | -2.75 | -5.78 | -1.27 |
| 1110 | DB06714 | -11.61 | -6.08 | -8.55 |
| 1111 | DB13944 | -17.01 | -12.87 | -10.17 |
| 1112 | DB01060 | -30.86 | -26.94 | -22.57 |
| 1113 | DB13946 | -12.94 | -11.89 | -8.80 |
| 1114 | DB01059 | -22.71 | -27.35 | -23.70 |
| 1115 | DB06716 | -10.18 | -11.71 | -8.15 |
| 1116 | DB13947 | -15.46 | -16.29 | -11.15 |
| 1117 | DB01061 | -28.78 | -33.13 | -30.87 |
| 1118 | DB06717 | -19.29 | -14.73 | -22.89 |
| 1119 | DB01063 | -24.13 | -27.22 | -19.38 |
| 1120 | DB06713 | -12.56 | -15.00 | -7.64 |
| 1121 | DB01064 | -22.23 | -16.26 | -17.93 |
| 1122 | DB06718 | -14.39 | -15.73 | -13.28 |
| 1123 | DB01062 | -14.24 | -11.48 | -11.31 |
| 1124 | DB06723 | -12.77 | -8.46 | -8.81 |
| 1125 | DB01065 | -15.46 | -16.68 | -15.77 |
| 1126 | DB06724 | -12.50 | -10.46 | -7.83 |
| 1127 | DB13952 | -13.32 | -19.96 | -12.46 |
| 1128 | DB01058 | -15.29 | -22.18 | -15.17 |
| 1129 | DB13953 | -15.62 | -18.72 | -14.47 |
| 1130 | DB01066 | -30.87 | -24.48 | -23.81 |
| 1131 | DB06725 | -20.19 | -23.76 | -17.85 |
| 1132 | DB13954 | -14.71 | -16.53 | -12.47 |
| 1133 | DB01067 | -26.65 | -26.58 | -24.02 |
| 1134 | DB01068 | -22.63 | -23.18 | -20.23 |
| 1135 | DB13956 | -12.68 | -16.13 | -10.74 |
| 1136 | DB01069 | -17.27 | -10.19 | -12.44 |
| 1137 | DB01071 | -14.08 | -15.98 | -13.61 |
| 1138 | DB06729 | -23.42 | -24.29 | -21.04 |
| 1139 | DB13955 | -9.58 | -10.26 | -4.46 |
| 1140 | DB01070 | -9.74 | -9.83 | -8.17 |
| 1141 | DB13960 | -13.89 | -13.82 | -12.58 |
| 1142 | DB01073 | -17.71 | -22.62 | -18.19 |
| 1143 | DB01074 | -14.46 | -13.53 | -9.76 |
| 1144 | DB01075 | -14.53 | -12.22 | -11.83 |
| 1145 | DB06730 | -10.99 | -13.31 | -9.60 |
| 1146 | DB13962 | -16.26 | -19.01 | -16.86 |
| 1147 | DB01077 | -15.03 | -14.99 | -14.40 |
| 1148 | DB13966 | -1.34 | -2.64 | 1.95 |
| 1149 | DB01072 | -13.87 | -22.61 | -13.73 |
| 1150 | DB06736 | -19.09 | -18.71 | -16.42 |
| 1151 | DB13969 | 7.16 | 6.04 | 9.19 |
| 1152 | DB01079 | -19.77 | -21.97 | -15.77 |
| 1153 | DB06738 | -17.84 | -14.76 | -12.03 |
| 1154 | DB13970 | 5.97 | 6.02 | 8.70 |
| 1155 | DB01080 | -16.83 | -16.51 | -13.26 |
| 1156 | DB06737 | -20.23 | -20.78 | -19.26 |
| 1157 | DB06739 | -13.79 | -15.56 | -10.96 |
| 1158 | DB13972 | -13.60 | -12.80 | -12.56 |
| 1159 | DB13977 | -12.50 | -10.46 | -7.83 |
| 1160 | DB01081 | -16.53 | -13.44 | -9.71 |
| 1161 | DB13967 | -20.43 | -21.07 | -8.69 |
| 1162 | DB01082 | -27.32 | -29.10 | -24.12 |
| 1163 | DB06742 | -16.51 | -21.63 | -11.71 |
| 1164 | DB01083 | -7.93 | -8.32 | -2.96 |
| 1165 | DB01086 | -12.68 | -15.19 | -12.60 |
| 1166 | DB13981 | -13.50 | -12.23 | -11.24 |
| 1167 | DB01085 | -13.92 | -14.82 | -14.41 |
| 1168 | DB01087 | -25.94 | -23.15 | -18.16 |
| 1169 | DB01084 | -21.64 | -17.16 | -17.27 |
| 1170 | DB01090 | -2.34 | -3.89 | -0.69 |
| 1171 | DB01088 | -13.93 | -12.47 | -13.07 |
| 1172 | DB01091 | -13.78 | -10.30 | -8.72 |
| 1173 | DB01093 | -8.10 | -9.16 | -5.45 |
| 1174 | DB01094 | -21.21 | -24.42 | -22.02 |
| 1175 | DB06756 | -8.36 | -7.21 | -9.95 |
| 1176 | DB01095 | -19.57 | -21.04 | -20.48 |
| 1177 | DB06751 | -9.62 | -7.98 | -3.69 |
| 1178 | DB01089 | -15.72 | -12.76 | -11.73 |
| 1179 | DB06762 | -19.64 | -21.38 | -18.44 |
| 1180 | DB13995 | -12.98 | -17.44 | -14.52 |
| 1181 | DB01097 | -15.55 | -19.50 | -14.60 |
| 1182 | DB06764 | -13.01 | -12.68 | -18.05 |
| 1183 | DB13996 | -9.17 | -10.12 | -8.92 |
| 1184 | DB01099 | -16.41 | -16.24 | -14.50 |
| 1185 | DB13997 | -19.68 | -21.42 | -20.54 |
| 1186 | DB01096 | -22.04 | -24.88 | -19.61 |
| 1187 | DB06767 | -11.21 | -9.68 | -8.75 |
| 1188 | DB01101 | -19.37 | -19.46 | -16.09 |
| 1189 | DB14001 | -7.79 | -10.45 | -3.21 |
| 1190 | DB01102 | -18.99 | -20.95 | -19.71 |
| 1191 | DB06768 | -12.81 | -12.43 | -11.46 |
| 1192 | DB14002 | -6.23 | -9.96 | -3.57 |
| 1193 | DB01098 | -22.85 | -24.03 | -18.27 |
| 1194 | DB06770 | -10.30 | -11.11 | -8.89 |
| 1195 | DB14006 | -7.45 | -6.60 | -3.30 |
| 1196 | DB01103 | -14.11 | -14.77 | -11.26 |
| 1197 | DB06769 | -10.34 | -11.92 | -12.15 |
| 1198 | DB14007 | -14.25 | -22.02 | -16.13 |
| 1199 | DB01105 | -6.03 | -3.45 | -3.80 |
| 1200 | DB06771 | -23.27 | -27.26 | -23.04 |
| 1201 | DB01100 | -20.81 | -22.43 | -18.33 |
| 1202 | DB06774 | -16.44 | -16.47 | -12.66 |
| 1203 | DB01104 | -16.64 | -15.66 | -13.27 |
| 1204 | DB06775 | -18.61 | -19.85 | -19.02 |
| 1205 | DB14011 | -11.26 | -11.48 | -7.86 |
| 1206 | DB01107 | -9.43 | -13.58 | -8.31 |
| 1207 | DB06778 | -9.91 | -10.66 | -9.39 |
| 1208 | DB01110 | -12.72 | -12.36 | -9.36 |
| 1209 | DB06777 | -12.56 | -16.86 | -12.92 |
| 1210 | DB01108 | -15.59 | -17.56 | -14.46 |
| 1211 | DB14015 | -12.21 | -11.50 | -8.67 |
| 1212 | DB01106 | -17.82 | -18.45 | -13.93 |
| 1213 | DB06782 | -10.89 | -9.64 | -7.56 |
| 1214 | DB01113 | -15.69 | -12.82 | -14.45 |
| 1215 | DB06780 | -17.12 | -17.37 | -12.95 |
| 1216 | DB14018 | -13.93 | -16.35 | -12.10 |
| 1217 | DB01114 | -12.79 | -12.72 | -9.02 |
| 1218 | DB06784 | -13.35 | -17.47 | -13.89 |
| 1219 | DB01112 | -26.62 | -27.83 | -24.01 |
| 1220 | DB01115 | -18.18 | -16.42 | -13.74 |
| 1221 | DB06781 | -8.76 | -14.69 | -13.06 |
| 1222 | DB01117 | -17.85 | -20.37 | -18.96 |
| 1223 | DB06787 | -17.37 | -11.44 | -11.59 |
| 1224 | DB01118 | -13.27 | -10.91 | -8.91 |
| 1225 | DB06786 | -14.52 | -16.86 | -11.27 |
| 1226 | DB01119 | -16.06 | -14.82 | -14.45 |
| 1227 | DB06766 | -20.00 | -23.04 | -18.18 |
| 1228 | DB14028 | -17.49 | -18.86 | -17.11 |
| 1229 | DB01116 | -13.40 | -14.72 | -16.50 |
| 1230 | DB06789 | -8.62 | -14.29 | -6.80 |
| 1231 | DB01121 | -18.82 | -19.68 | -13.45 |
| 1232 | DB06792 | -12.51 | -10.46 | -7.82 |
| 1233 | DB01120 | -21.67 | -25.55 | -17.43 |
| 1234 | DB01123 | -18.23 | -19.86 | -19.50 |
| 1235 | DB06794 | -24.20 | -27.60 | -26.96 |
| 1236 | DB14033 | -19.21 | -20.03 | -16.38 |
| 1237 | DB01124 | -17.92 | -20.40 | -15.94 |
| 1238 | DB06795 | -19.62 | -20.54 | -17.40 |
| 1239 | DB01125 | -14.98 | -18.96 | -18.91 |
| 1240 | DB06796 | -21.66 | -22.30 | -21.40 |
| 1241 | DB01122 | -9.63 | -13.31 | -6.72 |
| 1242 | DB06797 | -9.89 | -15.84 | -10.45 |
| 1243 | DB01127 | -11.98 | -12.94 | -9.36 |
| 1244 | DB06799 | -8.55 | -10.71 | -8.85 |
| 1245 | DB01128 | -23.79 | -20.23 | -18.41 |
| 1246 | DB06800 | -15.94 | -13.42 | -16.19 |
| 1247 | DB01126 | -22.28 | -20.18 | -19.46 |
| 1248 | DB06801 | -6.69 | -10.74 | -8.82 |
| 1249 | DB01129 | -20.34 | -22.26 | -16.64 |
| 1250 | DB06802 | -26.69 | -26.05 | -20.75 |
| 1251 | DB01131 | -23.02 | -30.68 | -15.83 |
| 1252 | DB01132 | -19.11 | -24.47 | -12.90 |
| 1253 | DB06803 | -26.99 | -23.41 | -22.24 |
| 1254 | DB01133 | -13.26 | -15.10 | -8.72 |
| 1255 | DB06807 | -16.51 | -18.19 | -17.87 |
| 1256 | DB01130 | -10.97 | -17.69 | -11.66 |
| 1257 | DB06804 | -1.83 | -13.98 | 7.26 |
| 1258 | DB01134 | -17.68 | -17.08 | -11.61 |
| 1259 | DB01136 | -21.31 | -18.50 | -17.79 |
| 1260 | DB06811 | -3.43 | -6.02 | 13.30 |
| 1261 | DB01092 | -20.27 | -19.34 | -19.08 |
| 1262 | DB06813 | -27.00 | -29.48 | -25.21 |
| 1263 | DB01138 | -17.35 | -23.20 | -17.44 |
| 1264 | DB06814 | -24.50 | -26.45 | -22.62 |
| 1265 | DB01139 | -23.36 | -22.59 | -19.20 |
| 1266 | DB06815 | -12.01 | -12.95 | -11.47 |
| 1267 | DB01137 | -25.56 | -28.78 | -24.85 |
| 1268 | DB06816 | -16.79 | -11.45 | -13.33 |
| 1269 | DB14061 | -21.89 | -20.14 | -20.01 |
| 1270 | DB01140 | -30.38 | -27.44 | -25.58 |
| 1271 | DB01142 | -16.90 | -15.93 | -14.17 |
| 1272 | DB06819 | -9.33 | -17.12 | -12.79 |
| 1273 | DB01143 | -17.57 | -19.59 | -15.23 |
| 1274 | DB01144 | -22.08 | -27.56 | -13.83 |
| 1275 | DB06820 | -14.12 | -12.09 | -7.50 |
| 1276 | DB01145 | -24.79 | -25.40 | -21.15 |
| 1277 | DB06821 | -21.47 | -20.91 | -18.91 |
| 1278 | DB01146 | -15.47 | -12.31 | -11.42 |
| 1279 | DB06823 | -11.45 | -16.78 | -13.40 |
| 1280 | DB01147 | -25.60 | -20.13 | -20.02 |
| 1281 | DB06824 | -33.11 | -29.95 | -26.76 |
| 1282 | DB01148 | -22.59 | -22.67 | -21.89 |
| 1283 | DB06826 | -14.32 | -18.24 | -12.80 |
| 1284 | DB01149 | -16.40 | -12.92 | -13.04 |
| 1285 | DB01150 | -33.28 | -29.25 | -25.28 |
| 1286 | DB01151 | -14.00 | -11.13 | -10.86 |
| 1287 | DB01153 | -15.16 | -12.88 | -13.28 |
| 1288 | DB01154 | -9.28 | -11.51 | -8.58 |
| 1289 | DB01155 | -23.47 | -24.14 | -19.89 |
| 1290 | DB01157 | -20.58 | -21.20 | -17.04 |
| 1291 | DB01158 | -2.56 | -4.19 | -5.25 |
| 1292 | DB01160 | -11.49 | -16.00 | -13.03 |
| 1293 | DB14082 | -22.41 | -25.94 | -21.72 |
| 1294 | DB01161 | -17.78 | -12.25 | -14.94 |
| 1295 | DB06827 | -33.74 | -36.24 | -25.22 |
| 1296 | DB01162 | -21.92 | -24.78 | -24.98 |
| 1297 | DB01166 | -20.43 | -19.79 | -20.46 |
| 1298 | DB14086 | -22.67 | -23.29 | -22.00 |
| 1299 | DB01165 | -27.43 | -27.71 | -24.78 |
| 1300 | DB01168 | -19.18 | -19.17 | -20.46 |
| 1301 | DB01170 | -21.07 | -19.58 | -16.19 |
| 1302 | DB01167 | -16.50 | -22.41 | -8.51 |
| 1303 | DB01171 | -20.19 | -20.21 | -13.21 |
| 1304 | DB01173 | -13.70 | -12.52 | -12.01 |
| 1305 | DB01172 | -19.20 | -19.70 | -16.57 |
| 1306 | DB01174 | -13.22 | -18.04 | -14.40 |
| 1307 | DB01175 | -19.46 | -14.70 | -12.37 |
| 1308 | DB01176 | -17.10 | -18.80 | -13.87 |
| 1309 | DB14104 | -3.71 | -5.22 | -3.71 |
| 1310 | DB01178 | -12.53 | -13.44 | -11.56 |
| 1311 | DB01179 | -14.50 | -13.67 | -12.15 |
| 1312 | DB01181 | -7.75 | -5.26 | -4.58 |
| 1313 | DB01182 | -22.75 | -23.03 | -18.74 |
| 1314 | DB01183 | -21.21 | -15.59 | -14.73 |
| 1315 | DB01184 | -24.17 | -25.02 | -19.18 |
| 1316 | DB01186 | -15.00 | -14.87 | -12.50 |
| 1317 | DB01185 | -15.99 | -16.13 | -14.49 |
| 1318 | DB01187 | -3.47 | -5.74 | -5.61 |
| 1319 | DB01188 | -12.84 | -17.18 | -14.05 |
| 1320 | DB01191 | -14.28 | -10.00 | -8.57 |
| 1321 | DB01190 | -15.14 | -23.51 | -15.34 |
| 1322 | DB01193 | -20.66 | -23.17 | -18.88 |
| 1323 | DB01192 | -22.98 | -17.90 | -18.39 |
| 1324 | DB01177 | -30.23 | -23.91 | -25.63 |
| 1325 | DB01194 | -22.65 | -19.21 | -17.22 |
| 1326 | DB14132 | -14.69 | -17.94 | -12.04 |
| 1327 | DB01197 | -15.13 | -14.87 | -14.39 |
| 1328 | DB01195 | -23.97 | -23.48 | -19.10 |
| 1329 | DB01196 | -10.80 | -10.77 | -5.61 |
| 1330 | DB01198 | -24.82 | -23.75 | -24.00 |
| 1331 | DB01202 | -11.35 | -16.04 | -11.71 |
| 1332 | DB01203 | -21.32 | -19.38 | -16.82 |
| 1333 | DB01204 | -43.85 | -43.14 | -31.90 |
| 1334 | DB01205 | -16.72 | -19.65 | -15.83 |
| 1335 | DB01206 | -13.54 | -19.25 | -15.07 |
| 1336 | DB01207 | -15.60 | -27.01 | -15.48 |
| 1337 | DB01208 | -25.70 | -25.81 | -23.52 |
| 1338 | DB01209 | -14.04 | -13.68 | -10.94 |
| 1339 | DB01210 | -19.19 | -18.82 | -20.20 |
| 1340 | DB14156 | -8.44 | -11.54 | -7.29 |
| 1341 | DB01212 | -32.66 | -33.62 | -27.39 |
| 1342 | DB01213 | -14.16 | -12.68 | -8.25 |
| 1343 | DB01214 | -15.68 | -17.95 | -12.23 |
| 1344 | DB14173 | -18.68 | -18.66 | -18.37 |
| 1345 | DB01215 | -17.25 | -15.52 | -16.30 |
| 1346 | DB01216 | -17.20 | -19.20 | -16.19 |
| 1347 | DB01217 | -16.23 | -17.87 | -12.76 |
| 1348 | DB01218 | -12.47 | -7.45 | -5.66 |
| 1349 | DB01219 | -25.65 | -34.78 | -28.41 |
| 1350 | DB01221 | -15.24 | -15.38 | -14.15 |
| 1351 | DB01222 | -13.69 | -18.81 | -12.13 |
| 1352 | DB01223 | -13.72 | -16.69 | -11.67 |
| 1353 | DB01224 | -19.96 | -23.67 | -18.15 |
| 1354 | DB01227 | -2.96 | -2.64 | -6.20 |
| 1355 | DB01228 | -22.77 | -23.89 | -20.39 |
| 1356 | DB01231 | -18.02 | -8.56 | -12.89 |
| 1357 | DB14185 | -13.96 | -15.04 | -3.11 |
| 1358 | DB01232 | -27.79 | -24.45 | -22.35 |
| 1359 | DB01233 | -20.32 | -18.07 | -14.03 |
| 1360 | DB01235 | -17.79 | -21.53 | -18.25 |
| 1361 | DB01234 | -16.47 | -18.68 | -15.88 |
| 1362 | DB01237 | -11.83 | -9.88 | -10.15 |
| 1363 | DB01239 | -16.37 | -12.81 | -12.32 |
| 1364 | DB01241 | -12.28 | -13.12 | -12.49 |
| 1365 | DB01238 | -17.93 | -22.05 | -18.82 |
| 1366 | DB01243 | -12.99 | -13.61 | -11.89 |
| 1367 | DB01242 | -12.08 | -11.02 | -10.31 |
| 1368 | DB01245 | 2.75 | 3.03 | 6.27 |
| 1369 | DB01246 | -11.22 | -9.56 | -11.81 |
| 1370 | DB01244 | -10.15 | -10.49 | -12.15 |
| 1371 | DB01247 | -18.60 | -22.85 | -17.83 |
| 1372 | DB01240 | -11.50 | -20.88 | -13.23 |
| 1373 | DB01250 | -28.51 | -28.37 | -27.88 |
| 1374 | DB01251 | -20.13 | -22.52 | -17.95 |
| 1375 | DB01253 | -17.27 | -24.43 | -15.92 |
| 1376 | DB01252 | -18.56 | -20.00 | -15.90 |
| 1377 | DB01255 | -20.76 | -20.10 | -20.40 |
| 1378 | DB01254 | -30.57 | -31.75 | -28.44 |
| 1379 | DB01259 | -30.91 | -35.14 | -29.35 |
| 1380 | DB01261 | -24.75 | -21.38 | -18.89 |
| 1381 | DB01262 | -17.87 | -22.07 | -20.72 |
| 1382 | DB01260 | -17.99 | -22.61 | -13.55 |
| 1383 | DB01256 | -12.70 | -20.37 | -6.53 |
| 1384 | DB01265 | -19.10 | -24.76 | -19.82 |
| 1385 | DB01264 | -20.29 | -21.06 | -17.36 |
| 1386 | DB01268 | -18.94 | -21.46 | -23.19 |
| 1387 | DB01273 | -17.66 | -17.66 | -16.04 |
| 1388 | DB01274 | -26.02 | -24.38 | -23.87 |
| 1389 | DB01275 | -29.81 | -22.24 | -20.49 |
| 1390 | DB01280 | -20.67 | -21.92 | -20.36 |
| 1391 | DB01267 | -17.13 | -23.02 | -17.27 |
| 1392 | DB01283 | -18.27 | -21.50 | -17.08 |
| 1393 | DB01263 | -22.43 | -19.08 | -4.22 |
| 1394 | DB01288 | -21.82 | -23.54 | -21.07 |
| 1395 | DB01291 | -16.60 | -19.26 | -17.86 |
| 1396 | DB01295 | -16.98 | -20.65 | -13.59 |
| 1397 | DB01297 | -18.94 | -20.23 | -15.92 |
| 1398 | DB01298 | -21.34 | -21.04 | -22.72 |
| 1399 | DB01299 | -19.49 | -20.19 | -21.04 |
| 1400 | DB01296 | -16.97 | -17.61 | -13.90 |
| 1401 | DB01303 | -7.47 | -6.96 | -3.37 |
| 1402 | DB01319 | -18.87 | -23.68 | -15.25 |
| 1403 | DB01320 | -16.24 | -15.01 | -13.31 |
| 1404 | DB01322 | -14.33 | -18.56 | -11.29 |
| 1405 | DB01301 | -20.51 | -16.18 | -17.87 |
| 1406 | DB01326 | -26.36 | -24.40 | -26.91 |
| 1407 | DB01327 | -29.81 | -29.28 | -21.23 |
| 1408 | DB01324 | -14.15 | -17.20 | -13.76 |
| 1409 | DB01325 | -17.63 | -18.34 | -17.39 |
| 1410 | DB01328 | -29.45 | -30.02 | -24.98 |
| 1411 | DB01329 | -33.67 | -40.48 | -22.54 |
| 1412 | DB01330 | -33.82 | -36.00 | -24.46 |
| 1413 | DB01331 | -20.45 | -22.57 | -19.61 |
| 1414 | DB01332 | -29.67 | -28.40 | -21.80 |
| 1415 | DB01333 | -23.85 | -27.79 | -28.49 |
| 1416 | DB01337 | -3.32 | -0.42 | -6.69 |
| 1417 | DB01339 | -4.89 | -5.21 | -3.88 |
| 1418 | DB01340 | -17.20 | -18.69 | -15.39 |
| 1419 | DB01346 | -18.10 | -19.69 | -15.82 |
| 1420 | DB01349 | -22.93 | -23.56 | -23.04 |
| 1421 | DB01351 | -10.99 | -15.15 | -9.90 |
| 1422 | DB01352 | -13.10 | -13.91 | -9.94 |
| 1423 | DB01353 | -10.96 | -15.18 | -10.18 |
| 1424 | DB01348 | -19.37 | -20.37 | -18.22 |
| 1425 | DB01355 | -12.08 | -15.54 | -10.98 |
| 1426 | DB01354 | -13.82 | -17.74 | -10.31 |
| 1427 | DB01357 | -13.90 | -11.36 | -11.22 |
| 1428 | DB01359 | -14.13 | -17.01 | -11.06 |
| 1429 | DB01364 | -14.97 | -18.67 | -13.91 |
| 1430 | DB01365 | -12.97 | -9.51 | -8.48 |
| 1431 | DB01366 | -24.33 | -21.48 | -18.29 |
| 1432 | DB01367 | -15.35 | -15.92 | -14.21 |
| 1433 | DB01362 | -30.68 | -38.23 | -21.82 |
| 1434 | DB01382 | -16.52 | -17.79 | -13.97 |
| 1435 | DB01380 | -13.94 | -24.68 | -13.65 |
| 1436 | DB01384 | -17.82 | -17.32 | -15.71 |
| 1437 | DB01390 | -12.78 | -11.33 | -7.87 |
| 1438 | DB01393 | -25.58 | -17.22 | -18.36 |
| 1439 | DB01392 | -17.28 | -18.74 | -16.84 |
| 1440 | DB01395 | -15.49 | -20.25 | -14.99 |
| 1441 | DB01397 | -14.90 | -16.85 | -15.93 |
| 1442 | DB01400 | -9.90 | -13.54 | -11.47 |
| 1443 | DB01399 | -19.37 | -18.44 | -15.02 |
| 1444 | DB01401 | -8.96 | -7.41 | -5.24 |
| 1445 | DB01403 | -16.57 | -13.54 | -11.68 |
| 1446 | DB01407 | -16.53 | -13.06 | -12.87 |
| 1447 | DB01406 | -15.46 | -15.38 | -9.70 |
| 1448 | DB01409 | -9.99 | -10.34 | -13.28 |
| 1449 | DB01408 | -19.64 | -20.26 | -20.50 |
| 1450 | DB01412 | -14.26 | -17.82 | -14.34 |
| 1451 | DB01410 | -11.80 | -19.31 | -13.98 |
| 1452 | DB01413 | -28.88 | -25.51 | -21.95 |
| 1453 | DB01414 | -20.37 | -26.09 | -22.30 |
| 1454 | DB01416 | -24.97 | -27.97 | -21.44 |
| 1455 | DB01411 | -27.06 | -29.40 | -21.73 |
| 1456 | DB01418 | -19.34 | -21.17 | -22.42 |
| 1457 | DB01415 | -32.09 | -30.27 | -31.17 |
| 1458 | DB01421 | -24.42 | -25.12 | -19.03 |
| 1459 | DB01422 | -19.40 | -19.58 | -16.87 |
| 1460 | DB01419 | -29.58 | -27.46 | -19.73 |
| 1461 | DB01424 | -15.96 | -17.39 | -15.56 |
| 1462 | DB01420 | -15.34 | -14.96 | -11.69 |
| 1463 | DB01423 | -14.35 | -16.31 | -16.89 |
| 1464 | DB01427 | -16.91 | -20.84 | -15.50 |
| 1465 | DB01425 | -18.11 | -24.43 | -18.03 |
| 1466 | DB01428 | -18.63 | -18.93 | -18.44 |
| 1467 | DB01429 | -13.26 | -10.88 | -8.33 |
| 1468 | DB01426 | -14.97 | -16.78 | -15.44 |
| 1469 | DB01433 | -6.97 | -4.73 | -3.47 |
| 1470 | DB01431 | -7.89 | -9.42 | -6.90 |
| 1471 | DB01435 | -13.30 | -16.62 | -12.30 |
| 1472 | DB01430 | -19.58 | -21.03 | -19.81 |
| 1473 | DB01438 | -24.95 | -23.00 | -23.43 |
| 1474 | DB01437 | -14.98 | -16.24 | -12.45 |
| 1475 | DB01440 | -9.15 | -11.08 | -10.84 |
| 1476 | DB01436 | -11.38 | -12.70 | -4.24 |
| 1477 | DB01452 | -14.43 | -15.00 | -16.05 |
| 1478 | DB01463 | -14.47 | -11.68 | -10.61 |
| 1479 | DB01466 | -16.73 | -13.85 | -11.30 |
| 1480 | DB01394 | -19.29 | -14.99 | -11.53 |
| 1481 | DB01482 | -16.42 | -17.26 | -12.25 |
| 1482 | DB01489 | -15.77 | -17.85 | -16.98 |
| 1483 | DB01495 | -13.38 | -16.62 | -12.39 |
| 1484 | DB01497 | -13.56 | -15.61 | -14.18 |
| 1485 | DB01501 | -18.46 | -22.75 | -13.49 |
| 1486 | DB01511 | -17.28 | -19.31 | -15.07 |
| 1487 | DB01541 | -11.37 | -16.12 | -11.05 |
| 1488 | DB01544 | -27.24 | -23.54 | -25.67 |
| 1489 | DB01535 | -17.97 | -16.57 | -13.10 |
| 1490 | DB01545 | -15.30 | -19.59 | -16.35 |
| 1491 | DB01550 | -18.63 | -17.31 | -10.96 |
| 1492 | DB01553 | -19.58 | -19.29 | -20.09 |
| 1493 | DB01551 | -15.60 | -14.71 | -15.47 |
| 1494 | DB01558 | -15.84 | -21.73 | -16.57 |
| 1495 | DB01559 | -16.54 | -13.61 | -13.92 |
| 1496 | DB01563 | -10.22 | -9.26 | -7.78 |
| 1497 | DB01567 | -16.55 | -15.53 | -15.32 |
| 1498 | DB01575 | -8.34 | -8.40 | -5.04 |
| 1499 | DB01577 | -12.50 | -9.91 | -9.69 |
| 1500 | DB01576 | -11.50 | -11.84 | -11.41 |
| 1501 | DB01581 | -21.96 | -19.11 | -17.64 |
| 1502 | DB01580 | -15.51 | -16.83 | -14.89 |
| 1503 | DB01582 | -22.03 | -18.58 | -18.01 |
| 1504 | DB01583 | -10.51 | -17.49 | -12.71 |
| 1505 | DB01578 | -19.90 | -25.56 | -16.24 |
| 1506 | DB01579 | -16.54 | -15.15 | -15.00 |
| 1507 | DB01589 | -14.21 | -12.39 | -11.69 |
| 1508 | DB01588 | -15.61 | -14.02 | -15.60 |
| 1509 | DB01587 | -15.75 | -14.36 | -13.43 |
| 1510 | DB01591 | -20.77 | -23.72 | -19.29 |
| 1511 | DB01586 | -15.31 | -17.34 | -15.25 |
| 1512 | DB01597 | -20.15 | -25.04 | -21.36 |
| 1513 | DB01595 | -22.71 | -24.27 | -22.12 |
| 1514 | DB01598 | -22.35 | -23.81 | -21.78 |
| 1515 | DB01594 | -21.21 | -19.04 | -17.94 |
| 1516 | DB01600 | -19.68 | -20.91 | -20.67 |
| 1517 | DB01602 | -26.53 | -23.59 | -25.03 |
| 1518 | DB01603 | -22.45 | -21.07 | -15.80 |
| 1519 | DB01606 | -19.72 | -17.09 | -16.95 |
| 1520 | DB01607 | -24.05 | -19.75 | -25.59 |
| 1521 | DB01604 | -21.92 | -26.01 | -18.98 |
| 1522 | DB01609 | -28.78 | -30.64 | -28.50 |
| 1523 | DB01610 | -21.30 | -21.91 | -18.22 |
| 1524 | DB01605 | -16.10 | -13.13 | -17.31 |
| 1525 | DB01612 | -8.44 | -10.26 | -8.51 |
| 1526 | DB01611 | -17.16 | -23.19 | -15.95 |
| 1527 | DB01613 | -17.57 | -15.57 | -13.19 |
| 1528 | DB01615 | -18.95 | -13.37 | -13.66 |
| 1529 | DB01616 | -10.28 | -9.30 | -8.38 |
| 1530 | DB01608 | -20.37 | -22.81 | -16.90 |
| 1531 | DB01618 | -19.61 | -21.11 | -18.39 |
| 1532 | DB01620 | -13.41 | -11.93 | -10.52 |
| 1533 | DB01614 | -15.58 | -17.42 | -12.93 |
| 1534 | DB01619 | -17.14 | -14.80 | -13.70 |
| 1535 | DB01623 | -23.60 | -26.96 | -20.31 |
| 1536 | DB01624 | -24.84 | -29.58 | -19.10 |
| 1537 | DB01625 | -8.26 | -9.55 | -11.63 |
| 1538 | DB01626 | -8.26 | -12.96 | -7.74 |
| 1539 | DB01622 | -25.10 | -28.95 | -19.13 |
| 1540 | DB01628 | -18.38 | -17.80 | -19.31 |
| 1541 | DB01621 | -19.87 | -26.49 | -13.98 |
| 1542 | DB01627 | -16.96 | -22.29 | -18.74 |
| 1543 | DB01632 | -18.76 | -24.99 | -21.58 |
| 1544 | DB01638 | -21.13 | -19.39 | -11.18 |
| 1545 | DB01656 | -19.74 | -16.41 | -15.41 |
| 1546 | DB01685 | -25.49 | -25.71 | -23.63 |
| 1547 | DB01708 | -13.44 | -16.01 | -11.37 |
| 1548 | DB01718 | 5.67 | 4.91 | 9.30 |
| 1549 | DB01744 | -8.26 | -11.12 | -8.27 |
| 1550 | DB01783 | -14.41 | -18.96 | -19.53 |
| 1551 | DB01839 | -14.10 | -11.31 | -9.88 |
| 1552 | DB01764 | -19.01 | -13.58 | -5.34 |
| 1553 | DB01878 | -14.64 | -16.03 | -13.77 |
| 1554 | DB01914 | -16.71 | -21.19 | -14.49 |
| 1555 | DB01942 | -9.61 | -10.79 | -7.18 |
| 1556 | DB01956 | -12.84 | -18.11 | -14.89 |
| 1557 | DB01987 | -20.88 | -20.91 | -13.17 |
| 1558 | DB07615 | -22.76 | -21.42 | -20.96 |
| 1559 | DB02187 | -16.47 | -19.82 | -14.22 |
| 1560 | DB07776 | -18.07 | -19.78 | -15.31 |
| 1561 | DB02300 | -10.06 | -17.01 | -8.27 |
| 1562 | DB02325 | -10.45 | -6.99 | -4.87 |
| 1563 | DB02362 | -15.50 | -21.08 | -13.65 |
| 1564 | DB02513 | -8.50 | -10.20 | -8.05 |
| 1565 | DB02530 | -15.42 | -15.06 | -15.17 |
| 1566 | DB02546 | -18.60 | -19.46 | -15.79 |
| 1567 | DB02659 | -16.25 | -17.47 | -16.00 |
| 1568 | DB02701 | -14.59 | -16.53 | -12.70 |
| 1569 | DB02709 | -22.82 | -19.54 | -18.38 |
| 1570 | DB02703 | -11.86 | -17.03 | -9.75 |
| 1571 | DB02772 | -17.26 | -24.40 | -14.53 |
| 1572 | DB02789 | -13.46 | -13.44 | -12.65 |
| 1573 | DB08439 | -20.52 | -20.91 | -17.96 |
| 1574 | DB02893 | -14.40 | -12.41 | -11.68 |
| 1575 | DB02925 | -20.88 | -22.96 | -17.45 |
| 1576 | DB02959 | -21.54 | -18.52 | -17.34 |
| 1577 | DB03006 | -18.01 | -17.44 | -19.00 |
| 1578 | DB03017 | -4.34 | -5.18 | -3.83 |
| 1579 | DB08604 | -11.79 | -13.90 | -10.31 |
| 1580 | DB03085 | -11.81 | -11.22 | -11.85 |
| 1581 | DB03088 | -14.25 | -16.83 | -13.51 |
| 1582 | DB03128 | -4.95 | -8.75 | -2.48 |
| 1583 | DB03147 | -31.50 | -35.15 | -35.31 |
| 1584 | DB03166 | -8.36 | -9.50 | -9.26 |
| 1585 | DB03175 | -8.83 | -5.72 | -3.54 |
| 1586 | DB03193 | -1.39 | -3.61 | -0.82 |
| 1587 | DB03209 | -19.04 | -21.22 | -17.79 |
| 1588 | DB08792 | -16.33 | -16.20 | -15.41 |
| 1589 | DB08797 | -19.23 | -19.70 | -15.79 |
| 1590 | DB08795 | -27.71 | -23.29 | -20.97 |
| 1591 | DB08798 | -20.78 | -18.01 | -18.06 |
| 1592 | DB08799 | -17.09 | -13.61 | -14.39 |
| 1593 | DB08800 | -14.10 | -10.96 | -10.35 |
| 1594 | DB08801 | -11.99 | -9.80 | -10.05 |
| 1595 | DB08803 | -14.72 | -13.42 | -11.56 |
| 1596 | DB08802 | -17.86 | -10.25 | -12.43 |
| 1597 | DB08796 | -19.72 | -17.63 | -19.97 |
| 1598 | DB08806 | -19.82 | -20.23 | -16.13 |
| 1599 | DB03247 | -23.33 | -25.54 | -22.70 |
| 1600 | DB08808 | -16.92 | -18.68 | -13.45 |
| 1601 | DB08807 | -20.36 | -18.20 | -17.33 |
| 1602 | DB08809 | -6.75 | -9.23 | -8.64 |
| 1603 | DB08804 | -13.86 | -13.58 | -9.85 |
| 1604 | DB08814 | -14.70 | -15.08 | -12.04 |
| 1605 | DB08810 | -29.56 | -23.07 | -21.13 |
| 1606 | DB08811 | -13.62 | -21.23 | -15.22 |
| 1607 | DB08815 | -17.95 | -20.15 | -15.97 |
| 1608 | DB03255 | -12.43 | -10.33 | -8.29 |
| 1609 | DB08816 | -23.03 | -30.30 | -24.14 |
| 1610 | DB08820 | -21.86 | -19.37 | -16.84 |
| 1611 | DB08819 | -11.27 | -12.09 | -10.39 |
| 1612 | DB08824 | -7.82 | -7.25 | -7.47 |
| 1613 | DB08826 | -11.65 | -13.54 | -12.73 |
| 1614 | DB08822 | -26.12 | -28.63 | -27.12 |
| 1615 | DB08818 | -19.14 | -18.06 | -15.82 |
| 1616 | DB08828 | -25.25 | -25.27 | -21.57 |
| 1617 | DB08834 | -14.82 | -19.29 | -13.24 |
| 1618 | DB08835 | -18.05 | -22.01 | -26.19 |
| 1619 | DB08827 | -30.53 | -23.58 | -23.53 |
| 1620 | DB08842 | -9.82 | -8.86 | -7.59 |
| 1621 | DB08847 | -18.80 | -17.96 | -15.49 |
| 1622 | DB08860 | -21.63 | -29.31 | -22.70 |
| 1623 | DB08864 | -34.12 | -28.95 | -22.79 |
| 1624 | DB08865 | -25.00 | -31.08 | -22.89 |
| 1625 | DB08868 | -14.31 | -16.75 | -6.90 |
| 1626 | DB08867 | -17.60 | -13.55 | -11.07 |
| 1627 | DB08872 | -11.73 | -16.16 | -11.38 |
| 1628 | DB08873 | -25.19 | -29.62 | -22.86 |
| 1629 | DB08875 | -23.35 | -29.70 | -15.77 |
| 1630 | DB08877 | -19.47 | -21.40 | -18.76 |
| 1631 | DB08880 | -17.97 | -23.36 | -16.10 |
| 1632 | DB08881 | -28.37 | -33.40 | -26.56 |
| 1633 | DB08882 | -18.43 | -24.87 | -18.19 |
| 1634 | DB08883 | -27.41 | -24.43 | -24.03 |
| 1635 | DB08884 | -8.80 | -13.00 | -9.74 |
| 1636 | DB08887 | -4.40 | -4.65 | 0.15 |
| 1637 | DB08889 | -28.39 | -12.17 | -15.84 |
| 1638 | DB08893 | -25.75 | -27.76 | -19.72 |
| 1639 | DB08895 | -20.68 | -28.68 | -22.79 |
| 1640 | DB08896 | -24.00 | -28.97 | -16.47 |
| 1641 | DB08897 | -12.68 | -7.83 | -8.45 |
| 1642 | DB08899 | -25.49 | -24.42 | -21.45 |
| 1643 | DB08901 | -27.10 | -28.15 | -18.91 |
| 1644 | DB08903 | -13.41 | -10.98 | -13.20 |
| 1645 | DB08905 | -17.98 | -17.31 | -12.80 |
| 1646 | DB08906 | -14.08 | -15.75 | -7.74 |
| 1647 | DB03310 | -33.10 | -32.31 | -26.64 |
| 1648 | DB08907 | -18.44 | -22.66 | -19.09 |
| 1649 | DB08908 | -8.88 | -12.69 | -7.21 |
| 1650 | DB08909 | -9.40 | -12.14 | -7.71 |
| 1651 | DB08910 | -19.76 | -21.75 | -19.34 |
| 1652 | DB08911 | -27.57 | -27.28 | -29.26 |
| 1653 | DB08912 | -23.65 | -27.53 | -21.71 |
| 1654 | DB08916 | -34.49 | -32.82 | -29.12 |
| 1655 | DB08918 | -9.23 | -9.29 | -12.34 |
| 1656 | DB08924 | -6.44 | -7.16 | -6.51 |
| 1657 | DB08922 | -18.89 | -20.90 | -14.39 |
| 1658 | DB08928 | -21.94 | -22.23 | -17.92 |
| 1659 | DB08931 | -23.38 | -26.76 | -21.48 |
| 1660 | DB08932 | -25.41 | -29.96 | -20.68 |
| 1661 | DB08930 | -21.87 | -23.70 | -18.01 |
| 1662 | DB08933 | -15.52 | -18.54 | -14.49 |
| 1663 | DB08936 | -17.73 | -15.29 | -12.51 |
| 1664 | DB08938 | -9.91 | -10.66 | -9.39 |
| 1665 | DB08934 | -20.45 | -23.64 | -18.31 |
| 1666 | DB08941 | -18.75 | -16.92 | -14.49 |
| 1667 | DB08943 | -12.49 | -10.29 | -11.00 |
| 1668 | DB08944 | -10.93 | -10.08 | -11.00 |
| 1669 | DB08946 | -11.77 | -9.30 | -11.00 |
| 1670 | DB08945 | -20.57 | -25.48 | -13.07 |
| 1671 | DB08947 | -22.29 | -26.96 | -14.82 |
| 1672 | DB08954 | -18.49 | -16.05 | -15.12 |
| 1673 | DB08957 | -31.38 | -26.04 | -25.10 |
| 1674 | DB08958 | -13.88 | -4.39 | -7.05 |
| 1675 | DB08964 | -15.62 | -16.39 | -11.23 |
| 1676 | DB08967 | -18.14 | -12.68 | -13.33 |
| 1677 | DB08968 | -23.63 | -20.62 | -21.51 |
| 1678 | DB08966 | -24.02 | -23.30 | -19.27 |
| 1679 | DB08970 | -14.67 | -18.96 | -13.85 |
| 1680 | DB08971 | -15.23 | -20.20 | -13.90 |
| 1681 | DB08974 | -24.73 | -29.67 | -24.57 |
| 1682 | DB08976 | -24.50 | -27.06 | -18.64 |
| 1683 | DB08973 | -13.31 | -20.32 | -11.86 |
| 1684 | DB08979 | -15.06 | -15.54 | -15.57 |
| 1685 | DB08981 | -19.14 | -19.28 | -14.66 |
| 1686 | DB08982 | -16.28 | -19.53 | -14.02 |
| 1687 | DB08984 | -20.54 | -23.22 | -17.78 |
| 1688 | DB08983 | -12.62 | -12.58 | -10.92 |
| 1689 | DB08987 | -9.66 | -9.15 | -10.76 |
| 1690 | DB08990 | -21.87 | -20.55 | -16.61 |
| 1691 | DB08991 | -15.77 | -17.10 | -14.24 |
| 1692 | DB08992 | -15.32 | -19.76 | -11.05 |
| 1693 | DB08988 | -11.78 | -13.80 | -11.08 |
| 1694 | DB08994 | -20.25 | -18.61 | -18.06 |
| 1695 | DB08965 | -7.48 | -9.05 | 4.17 |
| 1696 | DB08996 | -10.17 | -9.24 | -11.72 |
| 1697 | DB08995 | -27.02 | -27.77 | -21.72 |
| 1698 | DB08998 | -20.19 | -19.58 | -15.46 |
| 1699 | DB09000 | -14.58 | -16.39 | -14.63 |
| 1700 | DB09002 | -16.92 | -12.67 | -11.85 |
| 1701 | DB09003 | -18.89 | -18.44 | -22.03 |
| 1702 | DB09008 | -24.52 | -22.75 | -22.47 |
| 1703 | DB09009 | -15.94 | -16.16 | -14.98 |
| 1704 | DB09006 | -13.24 | -11.97 | -11.07 |
| 1705 | DB09012 | -21.22 | -25.68 | -18.08 |
| 1706 | DB09014 | -7.59 | -8.53 | -7.47 |
| 1707 | DB09015 | -15.83 | -15.90 | -13.19 |
| 1708 | DB09016 | -12.83 | -13.31 | -10.50 |
| 1709 | DB09018 | -18.17 | -16.48 | -15.29 |
| 1710 | DB09019 | -11.93 | -12.28 | -10.30 |
| 1711 | DB09017 | -13.61 | -14.60 | -15.46 |
| 1712 | DB09021 | -13.57 | -13.83 | -12.44 |
| 1713 | DB09026 | -17.88 | -16.69 | -7.02 |
| 1714 | DB09020 | -17.30 | -14.65 | -12.86 |
| 1715 | DB09028 | -14.66 | -16.16 | -12.80 |
| 1716 | DB09030 | -18.15 | -25.00 | -18.71 |
| 1717 | DB09031 | -1.93 | -2.13 | 2.51 |
| 1718 | DB09038 | -22.97 | -22.04 | -16.39 |
| 1719 | DB09039 | -15.69 | -14.16 | -15.13 |
| 1720 | DB09040 | -15.74 | -17.21 | -8.15 |
| 1721 | DB09041 | -13.14 | -13.86 | -12.83 |
| 1722 | DB09034 | -24.05 | -19.80 | -20.12 |
| 1723 | DB09042 | -24.87 | -28.17 | -21.52 |
| 1724 | DB09047 | -25.31 | -33.88 | -27.02 |
| 1725 | DB09048 | -16.22 | -18.59 | -10.87 |
| 1726 | DB09050 | -39.33 | -40.62 | -26.75 |
| 1727 | DB09049 | -17.99 | -12.58 | -12.05 |
| 1728 | DB09054 | -24.31 | -24.87 | -23.10 |
| 1729 | DB09055 | -16.98 | -16.30 | -13.94 |
| 1730 | DB09053 | -20.94 | -23.40 | -19.21 |
| 1731 | DB09056 | -10.34 | -7.32 | -7.00 |
| 1732 | DB09060 | -20.31 | -21.40 | -21.37 |
| 1733 | DB09061 | -7.84 | -10.31 | -11.18 |
| 1734 | DB09062 | -29.69 | -26.95 | -26.20 |
| 1735 | DB09063 | -32.37 | -28.67 | -20.90 |
| 1736 | DB09064 | -12.60 | -11.89 | -10.89 |
| 1737 | DB09065 | -22.03 | -16.50 | -14.55 |
| 1738 | DB09068 | -18.12 | -17.80 | -16.97 |
| 1739 | DB09069 | -15.79 | -18.04 | -14.32 |
| 1740 | DB09070 | -12.55 | -12.89 | -12.06 |
| 1741 | DB09071 | -14.94 | -17.87 | -10.59 |
| 1742 | DB09072 | -9.21 | -10.88 | -10.96 |
| 1743 | DB08993 | -16.74 | -25.82 | -18.39 |
| 1744 | DB09073 | -30.98 | -31.21 | -23.13 |
| 1745 | DB09074 | -25.12 | -27.19 | -24.31 |
| 1746 | DB09076 | -14.47 | -9.48 | -10.66 |
| 1747 | DB09078 | -25.08 | -29.40 | -17.57 |
| 1748 | DB09075 | -31.62 | -38.81 | -27.17 |
| 1749 | DB09080 | -23.33 | -23.55 | -21.37 |
| 1750 | DB09081 | -9.30 | -12.30 | -6.62 |
| 1751 | DB09079 | -37.67 | -37.77 | -32.21 |
| 1752 | DB09082 | -18.09 | -19.00 | -14.64 |
| 1753 | DB09084 | -18.28 | -15.99 | -13.57 |
| 1754 | DB09085 | -13.99 | -13.78 | -11.86 |
| 1755 | DB09083 | -17.17 | -14.16 | -12.37 |
| 1756 | DB09086 | -9.88 | -12.04 | -11.06 |
| 1757 | DB09087 | -9.90 | -10.66 | -9.39 |
| 1758 | DB09088 | -10.45 | -10.29 | -11.40 |
| 1759 | DB09089 | -11.29 | -15.31 | -9.02 |
| 1760 | DB09090 | -12.71 | -14.45 | -10.01 |
| 1761 | DB09092 | -16.36 | -19.35 | -13.54 |
| 1762 | DB09091 | -13.16 | -18.21 | -17.89 |
| 1763 | DB09093 | -22.27 | -17.46 | -20.28 |
| 1764 | DB09094 | -15.03 | -16.43 | -19.17 |
| 1765 | DB09096 | -21.84 | -20.72 | -19.67 |
| 1766 | DB09095 | -15.86 | -20.94 | -13.91 |
| 1767 | DB09101 | -18.20 | -17.71 | -16.26 |
| 1768 | DB09097 | -18.76 | -20.11 | -15.85 |
| 1769 | DB09104 | -12.77 | -8.30 | -8.81 |
| 1770 | DB09110 | -9.27 | -11.95 | -8.61 |
| 1771 | DB09112 | -11.63 | -11.69 | -7.44 |
| 1772 | DB09115 | -11.76 | -13.05 | -11.09 |
| 1773 | DB09116 | -9.89 | -13.56 | -9.72 |
| 1774 | DB09117 | -8.02 | -8.45 | -7.23 |
| 1775 | DB09118 | -15.06 | -15.76 | -13.35 |
| 1776 | DB09114 | 7.51 | 4.53 | 10.67 |
| 1777 | DB09120 | -15.74 | -16.52 | -12.26 |
| 1778 | DB09119 | -18.32 | -18.04 | -15.69 |
| 1779 | DB09124 | -10.43 | -13.80 | -9.51 |
| 1780 | DB09125 | -15.15 | -15.66 | -14.85 |
| 1781 | DB09128 | -24.33 | -22.15 | -21.54 |
| 1782 | DB09123 | -16.86 | -16.40 | -13.48 |
| 1783 | DB09133 | -21.21 | -26.35 | -23.59 |
| 1784 | DB09134 | -27.54 | -35.27 | -20.18 |
| 1785 | DB09135 | -26.01 | -32.32 | -19.43 |
| 1786 | DB09136 | -21.35 | -25.38 | -17.71 |
| 1787 | DB09137 | -19.48 | -21.00 | -15.13 |
| 1788 | DB09138 | -12.42 | -12.23 | -7.58 |
| 1789 | DB09139 | -16.25 | -16.90 | -11.71 |
| 1790 | DB09143 | -22.30 | -21.26 | -14.39 |
| 1791 | DB09144 | -15.51 | -20.79 | -12.91 |
| 1792 | DB09145 | -12.98 | -7.72 | -8.83 |
| 1793 | DB09146 | -14.22 | -21.69 | -15.19 |
| 1794 | DB09148 | -16.93 | -15.98 | -9.27 |
| 1795 | DB09149 | -16.37 | -21.91 | -16.59 |
| 1796 | DB09151 | -23.43 | -21.82 | -18.24 |
| 1797 | DB09154 | -12.61 | -17.55 | -14.34 |
| 1798 | DB09156 | -24.97 | -28.81 | -18.10 |
| 1799 | DB09157 | -7.19 | -7.54 | -4.50 |
| 1800 | DB09160 | 0.79 | -2.89 | 3.24 |
| 1801 | DB09161 | -3.24 | -3.08 | -2.59 |
| 1802 | DB09164 | -21.58 | -18.53 | -11.44 |
| 1803 | DB09165 | -14.56 | -11.80 | -9.23 |
| 1804 | DB09166 | -15.30 | -13.72 | -14.62 |
| 1805 | DB09167 | -16.40 | -13.49 | -11.43 |
| 1806 | DB09183 | -23.26 | -29.13 | -23.44 |
| 1807 | DB09185 | -18.65 | -18.31 | -14.72 |
| 1808 | DB09195 | -23.04 | -17.91 | -19.07 |
| 1809 | DB09197 | -19.18 | -21.01 | -16.86 |
| 1810 | DB09198 | -21.97 | -23.63 | -16.86 |
| 1811 | DB03585 | -19.24 | -20.50 | -17.89 |
| 1812 | DB09205 | -11.51 | -11.47 | -9.92 |
| 1813 | DB09209 | -19.21 | -17.40 | -17.01 |
| 1814 | DB09210 | -14.18 | -18.14 | -15.37 |
| 1815 | DB09211 | -24.46 | -20.72 | -17.83 |
| 1816 | DB09213 | -10.42 | -12.91 | -13.23 |
| 1817 | DB09212 | -13.99 | -19.02 | -14.64 |
| 1818 | DB09214 | -24.05 | -24.38 | -22.72 |
| 1819 | DB09216 | -19.03 | -24.15 | -22.98 |
| 1820 | DB09217 | -16.20 | -13.45 | -11.35 |
| 1821 | DB09218 | -22.26 | -23.73 | -22.50 |
| 1822 | DB09219 | -20.42 | -19.66 | -16.91 |
| 1823 | DB09220 | -17.47 | -17.61 | -22.12 |
| 1824 | DB09221 | -27.80 | -23.47 | -21.19 |
| 1825 | DB09224 | -16.42 | -18.85 | -13.54 |
| 1826 | DB09225 | -16.08 | -12.97 | -12.59 |
| 1827 | DB09223 | -17.82 | -18.64 | -16.39 |
| 1828 | DB09229 | -20.53 | -17.03 | -14.94 |
| 1829 | DB09227 | -29.75 | -26.59 | -23.14 |
| 1830 | DB09231 | -26.67 | -24.64 | -22.90 |
| 1831 | DB03615 | -19.74 | -30.41 | -18.02 |
| 1832 | DB09230 | -26.68 | -25.78 | -20.70 |
| 1833 | DB09232 | -26.15 | -21.74 | -21.41 |
| 1834 | DB03619 | -18.58 | -20.18 | -17.89 |
| 1835 | DB09235 | -25.99 | -23.30 | -23.83 |
| 1836 | DB09237 | -21.44 | -19.94 | -15.71 |
| 1837 | DB09236 | -15.96 | -16.49 | -12.25 |
| 1838 | DB09238 | -26.92 | -26.60 | -19.56 |
| 1839 | DB09241 | -12.53 | -13.58 | -10.56 |
| 1840 | DB09242 | -18.44 | -19.55 | -16.92 |
| 1841 | DB09245 | -19.03 | -17.60 | -18.41 |
| 1842 | DB09244 | -17.45 | -13.56 | -14.77 |
| 1843 | DB09132 | -22.50 | -19.95 | -18.08 |
| 1844 | DB09256 | -16.81 | -22.17 | -16.01 |
| 1845 | DB09257 | -13.22 | -17.10 | -13.44 |
| 1846 | DB09262 | -14.21 | -12.03 | -15.13 |
| 1847 | DB09267 | -22.51 | -18.68 | -19.64 |
| 1848 | DB09269 | -12.30 | -14.95 | -13.18 |
| 1849 | DB09268 | -26.32 | -17.94 | -16.65 |
| 1850 | DB09272 | -24.83 | -32.11 | -26.51 |
| 1851 | DB09273 | -14.33 | -15.20 | -10.77 |
| 1852 | DB03651 | -22.27 | -21.67 | -22.77 |
| 1853 | DB09274 | -26.08 | -21.26 | -16.08 |
| 1854 | DB09275 | -13.46 | -15.91 | -13.77 |
| 1855 | DB09277 | -5.52 | -7.44 | -5.58 |
| 1856 | DB09270 | 1.33 | -4.33 | 9.39 |
| 1857 | DB09279 | -20.61 | -21.00 | -18.22 |
| 1858 | DB09280 | -34.40 | -30.32 | -27.18 |
| 1859 | DB09283 | -11.27 | -11.26 | -10.20 |
| 1860 | DB09282 | -19.03 | -24.61 | -15.05 |
| 1861 | DB09285 | -22.72 | -27.44 | -19.38 |
| 1862 | DB09288 | -14.30 | -14.29 | -10.51 |
| 1863 | DB09286 | -20.08 | -23.34 | -17.59 |
| 1864 | DB09290 | -21.00 | -20.49 | -18.52 |
| 1865 | DB09291 | -20.87 | -23.80 | -18.14 |
| 1866 | DB09292 | -13.92 | -18.36 | -15.77 |
| 1867 | DB09289 | -18.53 | -11.76 | -20.05 |
| 1868 | DB09295 | -21.44 | -24.62 | -21.79 |
| 1869 | DB09299 | -16.98 | -19.18 | -11.01 |
| 1870 | DB09300 | -13.72 | -13.42 | -8.23 |
| 1871 | DB09304 | -17.97 | -16.52 | -15.68 |
| 1872 | DB09313 | -33.42 | -31.67 | -18.97 |
| 1873 | DB09314 | -12.98 | -7.72 | -8.83 |
| 1874 | DB09319 | -18.72 | -19.50 | -16.47 |
| 1875 | DB09320 | -22.20 | -21.38 | -17.94 |
| 1876 | DB09322 | -9.92 | -10.66 | -9.39 |
| 1877 | DB09323 | -22.14 | -18.68 | -19.41 |
| 1878 | DB09324 | -21.34 | -15.23 | -17.24 |
| 1879 | DB09326 | -10.54 | -8.90 | -8.43 |
| 1880 | DB09327 | -16.83 | -22.17 | -16.01 |
| 1881 | DB09330 | -35.26 | -27.71 | -21.07 |
| 1882 | DB09332 | -19.83 | -24.00 | -18.75 |
| 1883 | DB09333 | -13.36 | -13.17 | -14.16 |
| 1884 | DB09340 | -16.52 | -15.35 | -14.47 |
| 1885 | DB09342 | -17.85 | -15.09 | -13.24 |
| 1886 | DB09343 | -21.62 | -20.36 | -15.64 |
| 1887 | DB09345 | -10.10 | -16.75 | -10.93 |
| 1888 | DB09346 | -19.75 | -23.14 | -19.01 |
| 1889 | DB09348 | -9.95 | -10.10 | -7.28 |
| 1890 | DB09350 | -7.50 | -15.00 | -8.83 |
| 1891 | DB09351 | -14.40 | -15.92 | -11.45 |
| 1892 | DB09352 | -14.55 | -19.62 | -15.73 |
| 1893 | DB09355 | -21.28 | -24.67 | -20.93 |
| 1894 | DB09357 | -15.50 | -23.52 | -13.19 |
| 1895 | DB09364 | -16.77 | -20.99 | -18.39 |
| 1896 | DB09366 | -7.07 | -9.85 | -6.59 |
| 1897 | DB09371 | -17.05 | -16.10 | -10.85 |
| 1898 | DB09376 | -9.16 | -10.46 | -4.66 |
| 1899 | DB09378 | -14.29 | -19.14 | -15.59 |
| 1900 | DB09394 | -12.03 | -12.71 | -9.53 |
| 1901 | DB09383 | -15.29 | -20.88 | -16.07 |
| 1902 | DB09395 | -9.17 | -10.12 | -9.17 |
| 1903 | DB09403 | -17.90 | -22.51 | -19.65 |
| 1904 | DB09401 | -15.46 | -17.77 | -10.87 |
| 1905 | DB09409 | -9.17 | -10.13 | -9.17 |
| 1906 | DB09414 | -10.37 | -12.48 | -9.36 |
| 1907 | DB09413 | -11.93 | -11.59 | -9.42 |
| 1908 | DB09418 | -8.36 | -6.01 | -1.81 |
| 1909 | DB09419 | -13.80 | -20.18 | -13.58 |
| 1910 | DB09425 | -17.93 | -16.07 | -14.30 |
| 1911 | DB09449 | -11.93 | -11.59 | -9.42 |
| 1912 | DB09421 | -27.38 | -31.16 | -26.69 |
| 1913 | DB09460 | -12.50 | -10.46 | -7.83 |
| 1914 | DB09459 | -15.06 | -15.14 | -12.15 |
| 1915 | DB09472 | -9.91 | -10.66 | -9.39 |
| 1916 | DB09462 | -14.69 | -15.14 | -10.36 |
| 1917 | DB09473 | -14.45 | -13.31 | -12.39 |
| 1918 | DB09477 | -20.36 | -19.99 | -25.11 |
| 1919 | DB09481 | -12.50 | -10.46 | -7.83 |
| 1920 | DB03754 | -19.92 | -19.34 | -12.57 |
| 1921 | DB09488 | -27.59 | -27.94 | -29.46 |
| 1922 | DB09494 | 0.11 | -5.03 | 3.30 |
| 1923 | DB09389 | -13.90 | -15.02 | -8.57 |
| 1924 | DB09495 | -16.95 | -21.96 | -15.58 |
| 1925 | DB03756 | -5.34 | -8.90 | -6.16 |
| 1926 | DB09496 | -12.40 | -14.68 | -9.09 |
| 1927 | DB09499 | -11.45 | -13.08 | -9.55 |
| 1928 | DB09502 | -12.29 | -16.71 | -9.49 |
| 1929 | DB09510 | -11.58 | -13.05 | -11.85 |
| 1930 | DB09513 | -13.56 | -12.99 | -11.96 |
| 1931 | DB09512 | -3.75 | -5.00 | -3.74 |
| 1932 | DB03766 | -8.49 | -8.58 | -9.24 |
| 1933 | DB09516 | -11.16 | -11.09 | -9.34 |
| 1934 | DB09526 | -13.48 | -9.57 | -10.78 |
| 1935 | DB09534 | -17.17 | -19.63 | -16.59 |
| 1936 | DB09543 | -12.55 | -16.20 | -13.62 |
| 1937 | DB09546 | -15.75 | -18.97 | -17.24 |
| 1938 | DB09555 | -13.65 | -12.42 | -12.08 |
| 1939 | DB09561 | -10.65 | -14.47 | -13.13 |
| 1940 | DB09517 | -17.42 | -20.24 | -18.36 |
| 1941 | DB09563 | -5.14 | -7.38 | -5.61 |
| 1942 | DB09570 | -18.41 | -23.21 | -14.78 |
| 1943 | DB09571 | -12.29 | -9.88 | -8.57 |
| 1944 | DB09552 | -2.50 | -6.10 | 3.72 |
| 1945 | DB11068 | -12.69 | -9.55 | -7.17 |
| 1946 | DB11071 | -13.44 | -15.33 | -13.59 |
| 1947 | DB11079 | -12.24 | -17.69 | -10.00 |
| 1948 | DB11073 | 2.85 | -0.24 | 2.38 |
| 1949 | DB11091 | -4.65 | -5.03 | -4.05 |
| 1950 | DB11090 | -11.57 | -8.17 | -11.06 |
| 1951 | DB11085 | -14.53 | -11.69 | -11.17 |
| 1952 | DB03793 | -12.85 | -13.31 | -13.40 |
| 1953 | DB11098 | -12.78 | -11.33 | -7.87 |
| 1954 | DB11093 | -13.35 | -17.47 | -13.88 |
| 1955 | DB11102 | -17.66 | -16.77 | -19.79 |
| 1956 | DB11100 | -21.63 | -22.98 | -19.78 |
| 1957 | DB03796 | -2.13 | -5.51 | -2.50 |
| 1958 | DB11096 | -16.45 | -17.11 | -14.21 |
| 1959 | DB11110 | -13.35 | -17.47 | -13.65 |
| 1960 | DB11115 | -23.97 | -21.92 | -20.48 |
| 1961 | DB11121 | -8.63 | -9.61 | -8.43 |
| 1962 | DB11126 | -15.26 | -18.97 | -12.83 |
| 1963 | DB11125 | -5.21 | -6.76 | -3.56 |
| 1964 | DB11127 | -12.81 | -12.63 | -7.12 |
| 1965 | DB11129 | -11.68 | -13.01 | -11.98 |
| 1966 | DB11128 | -12.12 | -9.68 | -8.75 |
| 1967 | DB11141 | -15.15 | -19.93 | -14.52 |
| 1968 | DB11145 | -13.42 | -13.31 | -12.83 |
| 1969 | DB11150 | -9.91 | -10.66 | -9.39 |
| 1970 | DB11142 | -17.29 | -13.10 | -11.36 |
| 1971 | DB11148 | -14.45 | -14.90 | -11.59 |
| 1972 | DB11153 | -12.77 | -8.30 | -8.81 |
| 1973 | DB11151 | -12.77 | -8.30 | -8.81 |
| 1974 | DB11155 | -19.25 | -23.87 | -14.11 |
| 1975 | DB11154 | -14.84 | -17.02 | -13.67 |
| 1976 | DB11156 | -13.53 | -17.05 | -13.47 |
| 1977 | DB11160 | -15.24 | -16.14 | -10.62 |
| 1978 | DB11168 | -13.84 | -16.26 | -15.91 |
| 1979 | DB11164 | -15.31 | -15.35 | -10.63 |
| 1980 | DB11171 | -9.91 | -10.66 | -9.39 |
| 1981 | DB11175 | -15.75 | -14.85 | -13.84 |
| 1982 | DB11157 | -16.63 | -23.10 | -16.24 |
| 1983 | DB11180 | 0.03 | -3.96 | 2.50 |
| 1984 | DB11181 | -15.50 | -16.69 | -15.69 |
| 1985 | DB11185 | -28.30 | -23.62 | -21.13 |
| 1986 | DB11186 | -10.63 | -10.69 | -9.31 |
| 1987 | DB11189 | -13.10 | -13.87 | -12.01 |
| 1988 | DB11184 | -23.55 | -20.50 | -21.03 |
| 1989 | DB11183 | -27.86 | -32.33 | -17.16 |
| 1990 | DB11197 | -18.69 | -16.10 | -14.71 |
| 1991 | DB11200 | -13.10 | -13.88 | -12.01 |
| 1992 | DB11201 | -15.99 | -14.04 | -14.12 |
| 1993 | DB11190 | -24.03 | -30.58 | -16.26 |
| 1994 | DB03852 | -5.97 | -5.25 | -4.64 |
| 1995 | DB11205 | -20.77 | -22.15 | -18.92 |
| 1996 | DB11207 | -13.36 | -16.23 | -9.87 |
| 1997 | DB11210 | -13.10 | -13.87 | -12.01 |
| 1998 | DB11217 | -17.24 | -17.76 | -13.46 |
| 1999 | DB11219 | -14.64 | -15.87 | -10.49 |
| 2000 | DB11221 | -21.81 | -22.13 | -20.49 |
| 2001 | DB11226 | -15.06 | -17.82 | -14.63 |
| 2002 | DB11239 | -9.89 | -10.66 | -9.39 |
| 2003 | DB11235 | -12.98 | -16.60 | -10.43 |
| 2004 | DB11246 | -14.69 | -19.35 | -12.82 |
| 2005 | DB11248 | -15.26 | -18.97 | -12.83 |
| 2006 | DB11254 | -10.46 | -10.58 | -9.08 |
| 2007 | DB11256 | -26.21 | -30.28 | -27.50 |
| 2008 | DB11260 | -9.84 | -7.12 | -5.52 |
| 2009 | DB11263 | -17.20 | -21.54 | -19.05 |
| 2010 | DB11264 | -10.90 | -13.60 | -13.86 |
| 2011 | DB11268 | -16.80 | -17.89 | -16.53 |
| 2012 | DB11269 | -19.44 | -19.71 | -13.85 |
| 2013 | DB11272 | -6.23 | -8.50 | -2.78 |
| 2014 | DB11273 | -23.44 | -17.30 | -12.38 |
| 2015 | DB11274 | -22.60 | -15.08 | -11.63 |
| 2016 | DB11278 | -13.98 | -18.54 | -10.29 |
| 2017 | DB11244 | -6.83 | -6.91 | -3.55 |
| 2018 | DB11282 | -12.28 | -15.05 | -11.27 |
| 2019 | DB11279 | -11.43 | -8.28 | -9.04 |
| 2020 | DB11283 | 9.29 | 4.85 | 7.37 |
| 2021 | DB11296 | -29.84 | -28.16 | -28.64 |
| 2022 | DB11299 | -13.81 | -15.33 | -13.47 |
| 2023 | DB11284 | 4.23 | 0.30 | 8.94 |
| 2024 | DB11304 | -10.13 | -14.92 | -9.25 |
| 2025 | DB11315 | -13.78 | -16.31 | -10.43 |
| 2026 | DB11323 | -14.42 | -18.25 | -14.10 |
| 2027 | DB11327 | -15.55 | -16.32 | -11.64 |
| 2028 | DB11328 | -6.25 | -6.17 | -5.24 |
| 2029 | DB11336 | -19.20 | -24.09 | -20.87 |
| 2030 | DB03904 | -13.56 | -12.99 | -11.96 |
| 2031 | DB11348 | -11.36 | -11.32 | -9.29 |
| 2032 | DB11359 | -11.33 | -11.58 | -11.28 |
| 2033 | DB11362 | -11.46 | -24.64 | -17.77 |
| 2034 | DB11364 | -22.55 | -20.57 | -19.34 |
| 2035 | DB11363 | -18.59 | -20.60 | -22.49 |
| 2036 | DB11376 | -17.81 | -22.59 | -17.60 |
| 2037 | DB11386 | -4.68 | -4.21 | -3.66 |
| 2038 | DB11398 | -18.72 | -23.09 | -16.01 |
| 2039 | DB11371 | -17.54 | -16.39 | -14.30 |
| 2040 | DB11411 | -16.81 | -14.20 | -11.89 |
| 2041 | DB11421 | -17.11 | -16.28 | -18.88 |
| 2042 | DB11446 | -21.92 | -27.38 | -16.66 |
| 2043 | DB03929 | -16.93 | -17.98 | -15.28 |
| 2044 | DB11451 | -8.07 | -9.40 | -4.02 |
| 2045 | DB11454 | -11.25 | -15.78 | -10.23 |
| 2046 | DB11455 | -19.92 | -21.77 | -17.77 |
| 2047 | DB11460 | 1.95 | 0.42 | 4.98 |
| 2048 | DB11457 | -16.86 | -16.54 | -16.41 |
| 2049 | DB11477 | -16.88 | -16.52 | -15.98 |
| 2050 | DB11481 | -12.06 | -12.47 | -11.97 |
| 2051 | DB11490 | -16.89 | -14.80 | -15.42 |
| 2052 | DB11491 | -26.24 | -29.53 | -25.53 |
| 2053 | DB11512 | -27.55 | -24.18 | -29.80 |
| 2054 | DB11558 | -6.06 | -6.29 | -6.12 |
| 2055 | DB11560 | -20.78 | -19.14 | -18.50 |
| 2056 | DB11570 | -8.20 | -12.10 | -5.28 |
| 2057 | DB11573 | -12.98 | -8.57 | -8.83 |
| 2058 | DB11577 | -24.64 | -30.58 | -20.79 |
| 2059 | DB11582 | -20.55 | -19.39 | -16.62 |
| 2060 | DB11583 | 3.97 | 1.50 | 2.07 |
| 2061 | DB11584 | -17.18 | -14.30 | -14.33 |
| 2062 | DB11585 | -6.66 | -5.24 | -4.08 |
| 2063 | DB11586 | -15.13 | -0.32 | -6.03 |
| 2064 | DB11587 | -13.60 | -18.50 | -8.16 |
| 2065 | DB11591 | -17.07 | -18.79 | -19.18 |
| 2066 | DB11594 | -0.86 | -2.87 | 1.41 |
| 2067 | DB11596 | -35.44 | -34.92 | -28.26 |
| 2068 | DB11609 | -12.37 | -10.16 | -9.01 |
| 2069 | DB11610 | -15.27 | -16.56 | -16.36 |
| 2070 | DB11611 | -30.02 | -34.13 | -23.89 |
| 2071 | DB11619 | -13.04 | -12.01 | -10.85 |
| 2072 | DB11614 | -21.34 | -18.45 | -15.93 |
| 2073 | DB11622 | -21.55 | -21.26 | -20.58 |
| 2074 | DB11629 | -15.19 | -14.82 | -12.32 |
| 2075 | DB11632 | -28.99 | -30.11 | -28.04 |
| 2076 | DB11633 | -19.32 | -20.08 | -20.31 |
| 2077 | DB11636 | -13.29 | -15.80 | -14.31 |
| 2078 | DB11637 | -21.63 | -17.94 | -19.88 |
| 2079 | DB11638 | -15.01 | -16.28 | -15.77 |
| 2080 | DB11640 | -19.08 | -21.79 | -18.70 |
| 2081 | DB11642 | -12.88 | -15.90 | -8.55 |
| 2082 | DB11644 | -24.96 | -20.48 | -23.27 |
| 2083 | DB11660 | -18.68 | -21.62 | -12.63 |
| 2084 | DB11672 | -26.09 | -28.19 | -23.85 |
| 2085 | DB11691 | -16.37 | -23.61 | -16.66 |
| 2086 | DB04038 | -10.88 | -5.25 | -5.27 |
| 2087 | DB11699 | -21.79 | -16.45 | -16.23 |
| 2088 | DB11703 | -30.39 | -24.56 | -19.44 |
| 2089 | DB11705 | -24.29 | -27.74 | -19.07 |
| 2090 | DB11712 | -20.78 | -19.44 | -12.99 |
| 2091 | DB11718 | -26.72 | -33.83 | -26.08 |
| 2092 | DB11730 | -28.07 | -29.37 | -28.80 |
| 2093 | DB11735 | -14.97 | -21.86 | -12.70 |
| 2094 | DB11738 | -10.40 | -15.23 | -11.64 |
| 2095 | DB11742 | -20.15 | -23.25 | -8.16 |
| 2096 | DB11748 | -26.72 | -23.51 | -20.36 |
| 2097 | DB11750 | -14.18 | -14.58 | -13.85 |
| 2098 | DB11768 | -9.64 | -9.72 | -8.25 |
| 2099 | DB11737 | -21.25 | -21.29 | -23.11 |
| 2100 | DB04115 | -15.10 | -17.86 | -12.21 |
| 2101 | DB11791 | -24.01 | -28.89 | -23.69 |
| 2102 | DB11793 | -25.38 | -27.61 | -23.32 |
| 2103 | DB11799 | -22.66 | -19.92 | -17.52 |
| 2104 | DB11815 | -18.76 | -18.68 | -18.11 |
| 2105 | DB11817 | -23.62 | -23.08 | -21.20 |
| 2106 | DB11823 | -15.18 | -14.74 | -14.45 |
| 2107 | DB11827 | -17.40 | -21.59 | -17.15 |
| 2108 | DB11828 | -24.23 | -23.41 | -21.80 |
| 2109 | DB04160 | -14.18 | -11.72 | -9.93 |
| 2110 | DB04173 | -16.60 | -20.41 | -15.66 |
| 2111 | DB04209 | -13.36 | -11.15 | -4.83 |
| 2112 | DB04221 | 7.10 | 8.45 | 10.58 |
| 2113 | DB11901 | -25.32 | -25.94 | -19.14 |
| 2114 | DB04224 | -4.54 | -4.95 | -3.09 |
| 2115 | DB11915 | -16.12 | -15.86 | -10.45 |
| 2116 | DB11921 | -21.43 | -20.00 | -10.81 |
| 2117 | DB11943 | -26.23 | -29.29 | -23.52 |
| 2118 | DB04272 | -14.58 | -17.73 | -14.77 |
| 2119 | DB11967 | -23.81 | -31.28 | -24.73 |
| 2120 | DB11989 | -22.09 | -19.33 | -20.61 |
| 2121 | DB11994 | -20.76 | -19.40 | -17.43 |
| 2122 | DB12001 | -32.43 | -31.39 | -23.59 |
| 2123 | DB12007 | -15.56 | -20.49 | -16.12 |
| 2124 | DB12010 | -28.38 | -32.25 | -22.43 |
| 2125 | DB04335 | -21.50 | -26.16 | -21.55 |
| 2126 | DB04339 | -17.36 | -18.58 | -17.42 |
| 2127 | DB12070 | -22.59 | -20.08 | -14.85 |
| 2128 | DB04398 | -11.58 | -12.50 | -10.03 |
| 2129 | DB12095 | -23.51 | -23.06 | -17.15 |
| 2130 | DB12097 | -19.20 | -20.74 | -18.10 |
| 2131 | DB12107 | -19.99 | -23.49 | -16.90 |
| 2132 | DB12127 | -30.29 | -26.49 | -28.44 |
| 2133 | DB12161 | -18.84 | -14.65 | -13.15 |
| 2134 | DB04465 | -16.27 | -20.93 | -16.03 |
| 2135 | DB11526 | -35.68 | -27.81 | -37.04 |
