# Supplementary material for: Identification of repurposing therapeutics toward SARS-CoV-2 main protease by virtual screening
Source: PLoS One. 2022 Jun 30;17(6):e0269563. doi: 10.1371/journal.pone.0269563 (PMC9246117; doi:10.1371/journal.pone.0269563)
Supplement: S2 Table — (DOCX) [file pone.0269563.s006.docx]

S2 Table. Physicochemical property and drug-likeness predictions of lapatinib and its designed analogs.

| **Physicochemical Properties** | | | |
| --- | --- | --- | --- |
|  | **Lapatinib** | **Compound E** | **Compound F** |
| Formula | C_29_H_26_ClFN_4_O_4_S | C_28_H_22_ClF_2_N_3_O_2_ | C_27_H_20_ClF_2_N_3_O_2_ |
| Molecular weight | 581.06 g/mol | 505.94 | 491.92 |
| No. heavy atoms | 40 | 36 | 35 |
| No. aromatics/heavy atoms | 27 | 27 | 27 |
| No. rotatable bonds | 11 | 7 | 7 |
| No. H-bond acceptors (HBA) | 8 | 6 | 6 |
| No. H-bond donors (HBD) | 2 | 1 | 1 |
| No. rings | 5 | 5 | 5 |
| Molar Refractivity (MR) | 153.88 | 137.27 | 132.46 |
| Topological Polar Surface Area (TPSA) | 114.73 Å² | 60.18 Å² | 60.18 Å² |
| Log P_o/w_ (WLog P) | 7.34 | 8.96 | 8.39 |
| **Drug-likeness** | | | |
| Lipinski rule (77)  (MW ≤500 Da, HBD ≤5, HBA ≤10, Rotatable bond ≤10, PSA ≤140 Å and Log P ≤5) | No | No | No |
| Ghose rule (78)  (160 ≤ MW ≤ 480, -0.4 ≤ WLog P ≤ 5.6, 40 ≤ MR ≤130 and 20 ≤ atoms ≤ 70) | No | No | No |
| Veber rule (80)  (Rotatable bond ≤10 and PSA ≤ 140 Å) | No | Yes | Yes |
| MDDR Like rule (79)  (No. rings ≥ 3, No. rigid bonds ≥ 18 and Rotatable bond ≥ 6) | Yes | No | No |
| PAINS (81) | No | No | No |

Log P; Partition Coefficient
